# WERF Endometriosis Phenome and Biobanking Harmonisation Project for Experimental Models in Endometriosis Research (EPHect-EM-Pain): methods to assess pain behaviour in rodent models of endometriosis

**DOI:** 10.1093/molehr/gaaf023

**Published:** 2025-07-09

**Authors:** Kelsi N Dodds, Victor Fattori, Nick A Andrews, Caroline B Appleyard, Julie A Christianson, Raul Gomez, Stacy L McAllister, Stacey A Missmer, Jens Nagel, Paulina Nunez-Badinez, Michael S Rogers, Philippa T K Saunders, Miguel A Tejada, Katy Vincent, Lone Hummelshoj, Kaylon L Bruner-Tran, Erin Greaves, for the EPHect Experimental Models Working Group, Nick A Andrews, Michael S Anglesio, Caroline B Appleyard, Joe Arosh, Christian M Becker, Kaylon L Bruner-Tran, Katherine A Burns, Ronald L Chandler, Julie A Christianson, Fiona L Cousins, Kelsi N Dodds, Victor Fattori, Asgi Fazleabas, Caroline Gargett, Juan S Gnecco, Raul Gomez, Martin Götte, Erin Greaves, Linda G Griffith, Patrick G Groothuis, Ruth Grümmer, Sun-Wei Guo, Shannon M Hawkins, M Louise Hull, Lone Hummelshoj, Mark Hutchinson, Mohamed Gamal Ibrahim, Elizabeth E Marr, Stacy L McAllister, Stacey A Missmer, Jeffrey Mogill, Jens Nagel, Warren B Nothnick, Paulina Nunez-Badinez, Kevin G Osteen, Daniëlle Peterse, Michael S Rogers, Andrea Romano, Philippa T K Saunders, Miguel Ángel Tejada, Kathy L Sharpe-Timms, Waldiceu A Verri, Paola Viganó, Katy Vincent

**Affiliations:** College of Medicine and Public Health and Flinders Health and Medical Research Institute, Flinders University, Bedford Park, SA, Australia; School of Biomedicine and Institute for Photonics and Advanced Sensing, University of Adelaide, Adelaide, SA, Australia; Vascular Biology Program, Boston Children’s Hospital/Department of Surgery, Harvard Medical School, Boston, MA, USA; In Vivo Scientific Services, Salk Institute for Biological Studies, La Jolla, CA, USA; Department of Basic Sciences, Ponce Health Sciences University-Ponce Research Institute, Ponce, PR, USA; Department of Cell Biology and Physiology, University of Kansas Medical Center, Kansas City, KS, USA; Kansas Center for Metabolism and Obesity Research, Kansas City, KS, USA; Research Unit on Women’s Health-INCLIVA, Institute of Health Research, Valencia, Spain; Department of Pathology, University of Valencia, Valencia, Spain; Department of Gynecology and Obstetrics, Emory University School of Medicine, Atlanta, GA, USA; Department of Epidemiology, Harvard T.H. Chan School of Public Health, Boston, MA, USA; Department of Obstetrics and Gynecology, University of Michigan, Ann Arbour, MI, USA; World Endometriosis Research Foundation, London, UK; Exploratory Pathobiology, Research and Early Development, Bayer AG, Wuppertal, Germany; Non-clinical Sciences and Operations, Merz Therapeutics, Frankfurt, Germany; Exploratory Pathobiology, Research and Early Development, Bayer AG, Wuppertal, Germany; Vascular Biology Program, Boston Children’s Hospital/Department of Surgery, Harvard Medical School, Boston, MA, USA; Boston Center for Endometriosis, Boston, MA, USA; Centre for Inflammation Research, Institute for Regeneration and Repair, University of Edinburgh, Edinburgh, UK; Department of Pharmacology, Faculty of Medicine, University of Granada, Granada, Spain; Institute of Neuroscience, Biomedical Research Center, University of Granada, Armilla, Granada, Spain; Biosanitary Research Institute ibs.GRANADA, Granada, Spain; Nuffield Department of Women’s and Reproductive Health, University of Oxford, Oxford, UK; World Endometriosis Research Foundation, London, UK; World Endometriosis Research Foundation, London, UK; Department of Obstetrics and Gynecology, Vanderbilt University Medical Center, Nashville, TN, USA; World Endometriosis Research Foundation, London, UK; Division of Biomedical Sciences, Warwick Medical School, University of Warwick, Coventry, UK

**Keywords:** endometriosis, experimental models, rodents, pain, research, collaboration

## Abstract

Pain is a debilitating symptom of endometriosis, and its mechanisms are often explored using rodent models. However, a lack of harmonization amongst models and behavioural measures, in addition to inconsistent reporting, might limit the overall clinical relevance and hinder translation of findings. An additional challenge is accurately linking rodent behaviour to human experiences of endometriosis. This study aimed to: (i) review current measures of pain-associated behaviours used in endometriosis studies; (ii) recommend best practices for each method and their suitability to study endometriosis-associated pain; and (iii) develop internationally agreed-upon standard operating procedures (‘EPHect-EM-Pain SOPs’). The World Endometriosis Research Foundation (WERF) assembled an international working group, from which a ‘pain behaviour working group’ consisting of experts in the field was established. The group used additional consultation from experimental pain model scientists in the broader field. Stimulus-evoked (reflexive) and stimulus-independent (spontaneous) measures are currently used to assess pain-associated behaviours in rodents with experimental endometriosis. All existing methods offer advantages and limitations regarding ethological relevance, output quality, and equipment/training requisites. Internationally standardized pain SOPs as well as summary documentation outlining the minimum and standard requirements for several behavioural measures were developed, as well as consensus recommendations on experimental designs and documentation. To more closely reflect the lived experiences of those with endometriosis, the consortium recommends that, following validation, multiple types of pain-related and/or parallel rodent behaviours (e.g. anxiety) should be quantified as surrogate outcome measures for endometriosis-associated pain. These harmonized methods and documentation for endometriosis research will facilitate essential comparisons among studies, improve translational applicability, and provide a superior holistic view of animal (and thus human) wellbeing.

## Introduction

Endometriosis, which affects an estimated 190 million women worldwide, has a significant personal and societal burden due to its painful symptoms and fertility-related implications (Nnoaham *et al.*, 2011; Zondervan *et al.*, 2020). There is currently no known cure, although treatments are associated with low long-term success rates but have significant side effects (Johnson and Hummelshoj, 2013).

Although endometriosis is defined as endometrial-like tissue outside the uterus, this definition does not encompass the complex symptomatic, pathobiological, and multisystemic nature of the disease (Zondervan *et al.*, 2020). The high prevalence of endometriosis and its unknown aetiology highlight the need for a better understanding of the basic biology of endometriosis with regard to its development and persistence to enable subsequent identification of targets for more effective therapies.

Endometriosis lesions are heterogeneous, multicellular tissue deposits that typically contain endometrial-like stromal cells and epithelial glands resembling those of the endometrium, alongside extracellular matrix components such as fibrosis, scarring, and, in some cases, evidence of haemorrhage (Clement, 2007; Viganò *et al.*, 2018). The lesions are highly vascularized and innervated, with significant infiltration of immune cells, generating a profoundly inflammatory microenvironment (Greaves *et al.*, 2017a; Forster *et al.*, 2019; Hogg *et al.*, 2020, 2021; Panir *et al.*, 2022). Oestrogen plays a pivotal role in shaping the cellular composition and activity within lesions by influencing the endometrial-like cells (Saunders and Horne, 2021) as well as local neuroangiogenesis and neuroinflammation (Greaves *et al.*, 2014, 2015). Histologically, the morphology of lesions varies considerably, with differing degrees of fibrosis and extracellular matrix deposition (Viganò *et al.*, 2018), varying proportions of endometrial-like cells (Clement, 2007), and shifts in their synchronicity with the menstrual cycle (Colgrave *et al.*, 2020). The extent of immune and nerve infiltration also varies across lesions (Tran *et al.*, 2009). Given this diversity in lesion composition and microenvironment, it is likely that underlying disease mechanisms differ between lesions, which may explain the broad spectrum of clinical presentations and treatment responses as well as the dynamic nature of lesion progression. This variability is further reflected in the distinct appearances of lesions, often marked by deposits of different colours, which suggests a life cycle of lesion development and regression (Zondervan *et al.*, 2020).

### Endometriosis-associated pain: a complex challenge

Pelvic pain is the most common clinical symptom experienced by those with endometriosis, with 71–87% reporting chronic pelvic pain (Mao and Anastasi, 2010; Shafrir *et al.*, 2018). The pain experience during endometriosis is extremely variable, with endometriosis-associated pain (EAP) syndrome, including dysmenorrhoea (pain during menstruation), dyspareunia (pain during intercourse), dyschezia (pain during defecation), and dysuria (pain during urination), as well as ovulation pain and non-cyclic pelvic pain (Giudice, 2010). Symptoms can occur in isolation or in any combination, and it is widely reported that there is no direct correlation between disease presentation (based on the revised American Fertility Society [rAFS] staging) and the severity of pain experienced (Vercellini *et al.*, 2007). Endometriosis is also associated with several painful co-morbidities, including bladder pain syndrome, irritable bowel syndrome, abdominopelvic myofascial pain syndrome, vulval pain syndrome, fatigue, infertility, and migraine (As-Sanie *et al.*, 2019).

Pain is an unpleasant sensory and emotional experience, which occurs in response to actual or potential tissue damage. Pain in the absence of tissue damage (e.g. when putting a hand on a very hot surface) acts as an important early warning mechanism, protecting an individual from actual injuries or environmental danger. This type of pain generates adaptive learning intimately linked to negative emotions, reducing the potential of future injury (Woolf *et al.*, 1997; Verri *et al.*, 2006; Woolf and Ma, 2007; Fattori *et al.*, 2021). The sensation of pain is triggered by the activation of high-threshold primary sensory neurones, called nociceptors (Woolf and Ma, 2007). Inflammatory mediators commonly associated with pain can themselves activate and/or sensitize nociceptors such that a lower-intensity stimulus is required to generate an action potential. This might lead to allodynia (pain due to a stimulus that does not normally provoke pain) and/or hyperalgesia (increased pain from a stimulus that normally provokes pain). Sensitization resulting from a painful stimulus can occur at the peripheral site of the noxious stimulus or more remotely in the central nervous system (CNS). Both peripheral and central sensitization processes are normal mechanisms by which the organism understands the need to guard the injured area to prevent further damage and facilitate healing (Latremoliere and Woolf, 2009). Chronic pain, defined as persisting for at least 3 months, was previously thought to be maladaptive. However, emerging evidence suggests that it also serves an adaptive value, to produce hypervigilance to predation (Lister *et al.*, 2020).

In those with endometriosis, chronic pelvic pain represents a major clinical problem with significantly lower quality of life and mental health (Facchin *et al.*, 2015). It has been estimated that patients lose an average of 11 hours of work each week, which is mainly related to reduced effectiveness during working time due to pain (presenteeism) (Nnoaham *et al.*, 2011; Soliman *et al.*, 2017). This debilitating chronic pelvic pain can also contribute to the higher rates of depressive symptoms (Gambadauro *et al.*, 2019), and in adolescent patients, it is associated with higher rates of anxiety, depression, and severe impacts on educational and professional achievements and wider social health (Gallagher *et al.*, 2018; Missmer *et al.*, 2022; Sasamoto *et al.*, 2023).

### Mechanisms underlying endometriosis-associated pain

There are many excellent reviews that describe what we currently understand about pain mechanisms in endometriosis (Coxon *et al.*, 2018; Maddern *et al.*, 2020; McNamara *et al.*, 2021). In brief, the presence of ectopic endometrial-like tissue can act as one of the triggers for pain. Lesions and/or adjacent tissues become innervated such that communication between the ectopic tissue, immune cells, and lesion-innervating nociceptors causes pain and inflammation (Stratton and Berkley, 2011). Nociceptors can be activated by the inflammatory milieu present in the lesion and peritoneal cavity. As a result of this prolonged and sustained stimulation, associated maladaptive changes both in the peripheral nervous system and CNS occur (Coxon *et al.*, 2018). Several other local factors likely contribute to pain diversity, including the lesion subtypes present, differences in lesion composition and location, and the presence of adhesions, extra-uterine bleeding, and neuropathic pain (Greaves *et al.*, 2020). Given that most patients will have to undergo surgery at least once to make a definitive diagnosis of endometriosis, and potentially several times as a treatment, there is also likely to be a component of post-surgical pain for many (Schug *et al.*, 2019). In addition to these factors, peripheral cross-organ sensitization also contributes to the complex pain syndromes and co-morbidities in those with endometriosis.

### Evaluating pain in experimental models of endometriosis

Rodent models of experimental endometriosis have been used to map mechanisms and pathways that contribute to pain generation arising from the presence of ectopic endometrial-like tissue. These include nerve cell growth, inflammation, neuroangiogenesis, and changes in CNS activation (Laux-Biehlmann *et al.*, 2015; McKinnon *et al.*, 2015). However, the challenge of linking these mechanisms to behavioural endpoints as a surrogate for pain experience remains unresolved. Moreover, a set of robust outcome measures to be used in preclinical studies that interrogate the potential of new therapies has yet to be established. To add to the challenge, there are many variations of rodent models of experimental endometriosis being used in the field, all using different measures of pain and wellbeing (Nunez-Badinez *et al.*, 2021).

Direct measures of pain behaviour can be grouped into two categories: stimulus-evoked (reflexive) and stimulus-independent (spontaneous) (Nunez-Badinez *et al.*, 2021; Tejada *et al.*, 2023). In brief, stimulus-evoked measures reported in endometriosis studies have measured mechanical (von Frey filaments), thermal (Hargreaves or hot plate tests), and visceral hypersensitivity (visceral motor reflex or vaginal escape responses). Stimulus-independent, or non-evoked, measurements have included three main behaviours: abdominal squashing, contortions, or licking. Additionally, measurements of animal wellbeing, which closely mirror ethological behaviour, such as nest building, burrowing, locomotion, and thigmotaxis, are increasingly being pursued in the wider pain field (Stevens Negus, 2019; Eisenach and Rice, 2022; Sadler *et al.*, 2022). These non-evoked measures may be considered more reflective of intrinsic pain and therefore relevant for the chronic pain symptoms experienced by individuals with endometriosis. Home cage analysis (HCA), which has also been applied to infer endometriosis-related discomfort (Tejada *et al.*, 2022), can measure a range of different ethological behaviours via a microchip and is completely independent of an observer in both light and dark phases.

### Pain and related behavioural tests in future endometriosis research: a unified approach

In recognizing the lack of harmonization of rodent models of experimental endometriosis and behavioural endpoints, as well as the issues regarding reproducibility that this implies, the World Endometriosis Research Foundation (WERF) introduced an Experimental Models (EM) section to the Endometriosis Phenome and Biobanking Harmonisation Project (EPHect) (‘EPHect-EM’). The aim of the initiative was to develop internationally agreed-upon standard operating procedures (SOPs) for the implementation of experimental endometriosis and recommendations for model design and to set reporting standards in homologous, heterologous, and emerging models (Burns *et al.*, 2025; Hull *et al.*, 2025; Marr *et al.*, 2025). The aims of the ‘pain behaviour working group’ (see Methods section) were to review current tests of pain-associated behaviours in rodent models of endometriosis and evaluate their suitability and reproducibility. Here, we describe measures for assessing pain-associated behaviour and wellbeing in mice and rats, including stimulus-evoked and stimulus-independent tests, as well as those related to wellbeing (e.g. measures of anxiety, distress, and depression). Subsequently, recommendations of best practice for rodent behavioural measures of EAP were developed (including robust endpoints for preclinical studies), in addition to harmonized documentation and EPHect-EM-Pain SOPs.

Recommendations and SOPs were also developed for heterologous rodent models, homologous rodent models, and endometriosis organoids, and the results are presented in the associated EPHect companion papers (Hull *et al.*, 2025; Burns *et al.*, 2025; and Marr *et al.*, 2025, respectively).

## Methods

The World Endometriosis Research Foundation (WERF) Endometriosis Phenome and Biobanking Harmonisation Project (EPHect) previously established a standard recommended and minimum requirements for collecting clinical and surgical data from those with endometriosis (Becker *et al.*, 2014; Vitonis *et al.*, 2014), as well as a recent standardized physical examination assessment for endometriosis (Lin *et al.*, 2024). WERF also developed internationally agreed-upon SOPs for collecting, processing, and storing tissue and fluid biospecimens (Fassbender *et al.*, 2014; Rahmioglu *et al.*, 2014). Through this process, WERF identified the need to also develop standards of practice in experimental models of endometriosis.

Thus, in 2021, WERF initiated the EPHect Experimental Models (EPHect-EM) project with the aim of analysing and assessing experimental models for their ability to demonstrate the key hallmarks of endometriosis disease and to provide outcomes that include reliable visualization of lesions and measurements of pain and/or fertility.

Leading researchers specializing in experimental models of endometriosis were invited to join the EPHect-EM working group. Two workshops were held in May and June 2021 to discuss variations in animal models, evaluating their advantages and limitations. Sub-groups were then formed based on specific areas of expertise. Selection criteria for working group members included pioneering contributions to rodent models of endometriosis and a track record of published research demonstrating: (i) comprehensive histological analysis, (ii) strong experimental design, and (iii) at least three studies using the same model. For emerging models, such as genetic models and organoids, eligibility was based on a single comprehensive publication and a strong reputation in a related field. A total of 44 experts were invited, with 39 accepting the invitation. Additionally, six observers (students or early-career researchers) were included. Among the original 39 members, 27 and 29 attended the respective online workshops in June 2021. Subsequently, eight new members joined, while three withdrew, resulting in a final group of 44 contributors.

The aims of the workshops were to reappraise current and emerging experimental models of endometriosis, assess endpoint measurements, and develop criteria (i.e., SOPs) that meet a minimum set of standards based on the study objective(s). Following the workshops, working group members were assigned to one or more working groups based on their affinity (Supplementary Fig. S1). The four groups were: (i) heterologous models; (ii) homologous models; (iii) pain behaviour; and (iv) emerging (*in vitro*) models. For each group, a discussion forum was used to submit SOPs for circulation and standardization and to enable a platform for further discussion. Additional meetings were scheduled where required to continue the dialogue and reach consensus on areas for unification. For the pain behaviour working group, such meetings were organized to allow for consultation with pain researchers working outside the field of endometriosis.

There were 28 protocols received from members of the working group, representing the full spectrum of pain and wellbeing measures used in the endometriosis field, as well as other protocols co-opted from the wider pain community. Comprehensive EPHect-EM-Pain SOPs (Supplementary File S1) were assembled to incorporate sufficient detail to replicate protocols with ease and sufficient knowledge of the habituation practices required.

## Results

Effectively measuring pain-associated behaviours in animals is perhaps the most difficult challenge in the field of pain and is most frequently based on assessment of allodynia or hyperalgesia. Given the visceral nature of endometriosis, many available measures of pain-associated behaviours do not directly measure this type of pain, but rather the more somatic and referred components of the EAP syndrome. Below, we summarize key considerations for the choice of measures used in the assessment of pain in rodents (categorized into stimulus-evoked, stimulus-independent, and ethological behaviours); crucial aspects of experimental design for behavioural studies; and the major strengths and weaknesses of each different test (stimulus-evoked pain: Table 1; stimulus-independent pain: Table 2; ethological behaviours: Table 3). We also provide internationally agreed-upon EPHect-EM-Pain SOPs, including harmonized documentation outlining minimum and standard requirements for several methods, with the aim to improve reproducibility between investigators and studies (Supplementary File S1).

**Table 1.** Summary of stimulus-evoked pain behavioural tests in rodent models of endometriosis.

Behavioural test	Pros	Cons	Species	References
**von Frey filament** *EPHect-EM-Pain SOP 2*	Rat and mouse models Evident in both surgical and non-surgical models of endometriosis Filaments can be applied to the paw and abdominal regions Remains the gold standard test for mechanical hyperalgesia	Can be time consuming Does not reflect the main symptomatic feature of patients with endometriosis Requires extensive training	Mouse	(Greaves *et al.*, 2017b; Forster *et al.*, 2019; Fattori *et al.*, 2020; Dorning *et al.*, 2021; Escudero-Lara *et al.*, 2021; McAllister *et al.*, 2021)
			Rat	(Liu *et al.*, 2019)
**Electronic version of the von Frey filaments** *EPHect-EM-Pain SOP 3A*	Less time consuming than the von Frey filaments Filaments can be applied to the paw and abdominal regions	Does not reflect the main symptomatic feature of patients with endometriosis Mechanical pressure detected is significantly different from the von Frey filaments Has not been shown in endometriosis models Requires extensive training	Mouse	(Colombo *et al.*, 2018)
**Electronic von Frey** *EPHect-EM-Pain SOP 3B*	Less time consuming than the von Frey filaments Force application is automated	Does not reflect the main symptomatic feature of patients with endometriosis Evident in surgical rat models of endometriosis only Filaments can be only applied to the paw Mechanical pressure detected is significantly different from the von Frey filaments Requires extensive training	Rat	(Vivancos *et al.*, 2004; Martinov *et al.*, 2013; Liu *et al.*, 2018; Ge *et al.*, 2019)
**Visceromotor reflex (VMR) to vaginal distension** *EPHect-EM-Pain SOP 4*	Rat and mouse models Measures vaginal hyperalgesia and allodynia Provides closest measurement for human visceral pain	Requires extensive training Requires surgery to implant the balloon	Mouse	(Alali *et al.*, 2020; Castro *et al.*, 2021; Maddern *et al.*, 2022)
			Rat	(Nagabukuro and Berkley, 2007; Dmitrieva *et al.*, 2012; Ge *et al.*, 2019)
**Escape response to vaginal distension** *EPHect-EM-Pain SOP 5*	Measures vaginal hyperalgesia and allodynia Does not require surgery to implant the balloon	Rat models only Evident in surgical models of endometriosis Requires extensive training Can be time consuming	Rat	(Berkley *et al.*, 2001; McAllister *et al.*, 2009, 2012, 2016)
**Hargreaves** *Pain-SOP 6*	Relatively easy to perform	Sensitivity to heat stimuli rarely reported by individuals with endometriosis Differences in heat sensitivity between sham and endometriosis mice are evident only in surgical mouse models of endometriosis	Mouse	(Yan *et al.*, 2019; Fattori *et al.*, 2020; McAllister *et al.*, 2021)
**Hot plate** *EPHect-EM-Pain SOP 7*	Rat and mouse models Relatively easy to perform	Sensitivity to heat stimuli rarely reported by individuals with endometriosis. Presents a learning component inherent to the test	Mouse	(Lu *et al.*, 2010; Du *et al.*, 2017; Cao *et al.*, 2019; Zheng *et al.*, 2023)
			Rat	(Hernandez *et al.*, 2015, 2017; Cordaro *et al.*, 2021)

**Table 2.** Summary of stimulus-independent pain behavioural outcomes in rodent models of endometriosis.

Behavioural outcome	Pros	Cons	Species	References
**Abdominal squashing** *EPHect-EM-Pain SOP 8*	Easy to score Rat and mouse models It is only present during endometriosis pain (sham/naïve animals do not display this behaviour)	Reported only in the non-surgical mouse model If a suitable recording device is not available, analysis needs to be done live Bottom-up video recording can facilitate visualization scoring	MouseRat	(Fattori *et al.*, 2020)(Lopopolo *et al.*, 2014; Di Paola *et al.*, 2016; Iuvone *et al.*, 2016; Mvondo *et al.*, 2017; Pereira *et al.*, 2019)
	•	•		
**Abdominal contortions** *EPHect-EM-Pain SOP 9*	Easy to score Hard to mislabel, reducing false positives Present only during endometriosis pain (sham/naïve animals do not display this behaviour).	Inconsistencies for rat models—studies score this behaviour in the presence of a second stimulus, e.g. acetic acid If a proper recording device is not available, analysis needs to be done live Bottom-up video recording can facilitate visualization scoring	MouseRat	(Fattori *et al.*, 2020)(Lopopolo *et al.*, 2014; Di Paola *et al.*, 2016; Iuvone *et al.*, 2016; Mvondo *et al.*, 2017; Pereira *et al.*, 2019)
	•	•		
**Abdominal licking** *EPHect-EM-Pain SOP 10*	Relatively easy to score (with practice and good visual devices) Evident in both surgical and non-surgical models of endometriosis.	It can be easily mislabelled by non-trained investigators Levels can be high due to non-pain-related causes, e.g. dermatitis, dirty cage leading to dirty fur, urination in the test cage leading to dirty fur Depends on good visual devices to capture/record it, Scoring live may compromize accurate analysis	MouseRat	(Greaves *et al.*, 2017b; Forster *et al.*, 2019; Fattori *et al.*, 2020; McAllister *et al.*, 2021)(Lopopolo *et al.*, 2014; Di Paola *et al.*, 2016; Iuvone *et al.*, 2016)

**Table 3.** Summary of ethological behavioural tests in rodent models of endometriosis.

Behavioural test	Pros	Cons	Species	References
**Thermal gradient** *EPHect-EM-Pain SOP 11*	Provides analysis of freely moving animals without human presence Does not require extensive training Relatively easy to perform	Evident only in non-surgical mouse model Time consuming (both assay and data analysis)	Mouse	(Fattori *et al.*, 2020)
**Nesting** *EPHect-EM-Pain SOP 12*	Provides analysis of freely moving animals Does not require extensive training Relatively easy to perform	Evident only in surgical mouse models of endometriosis		(Tejada *et al.*, 2022)
**Burrowing** *EPHect-EM-Pain SOP 13*		Evident in only surgical mouse model Behavioural change is time-dependent (occurs after 15 dpi) Animals need to be tested for a baseline burrowing ability. Animal pair swapping might be required Test is time-sensitive		(Bashir *et al.*, 2023)
**Home cage analysis** *EPHect-EM-Pain SOP 14*	Provides analysis of freely moving animals without human presence Enables animal monitoring over extensive period of time Does not require extensive training	Mouse models only Evident in surgical models of endometriosis only Implantation place is critical since it can produce unreliable readings in case of chip misplacement Excessive bedding (must be kept to a maximum of 0.5 cm) also interferes with microchip detection		(Tejada *et al.*, 2022)
**Open field** *EPHect-EM-Pain SOP 15*	Provides analysis of freely moving animals Does not require extensive training Relatively easy to perform	Evident only in surgical mouse models of endometriosis Only mild differences in anxiety are reported by mice with endometriosis		(Cordaro *et al.*, 2021; McAllister *et al.*, 2021)
**Exploratory behaviour** *EPHect-EM-Pain SOP 16*				(Greaves *et al.*, 2017b; McAllister *et al.*, 2021)
**Elevated plus maze** *EPHect-EM-Pain SOP 17*		Evident only in surgical mouse models of endometriosis Only mild differences in anxiety are reported by mice with endometriosis		(Escudero-Lara *et al.*, 2021; Nunez-Badinez *et al.*, 2023)
**Elevated zero maze** *EPHect-EM-Pain SOP 18*	Provides analysis of freely moving animals Does not require extensive training Relatively easy to perform Do not have a centre zone to eliminate any ambiguity this might cause	Evident only in surgical rat models of endometriosis Only mild differences in anxiety are reported by rats with endometriosis	Rat	(Torres-Reverón *et al.*, 2018a,b)

### Experimental design

#### Habituation, animal handling, and training

Environmental stressors within the laboratory are a well-recognized source of unexplained background variation that influences behavioural testing (Sorge *et al.*, 2014; Gouveia and Hurst, 2017). These factors may include the presence and sex of an experimenter, housing conditions (e.g. light cycle), the behavioural testing space (e.g. noise levels, scents including cleaning agents), and the time-of-day when tests are performed. When placed in a novel environment, rodents tend to perform exploratory behaviours that can be reduced over time. This process is known as habituation, which may be induced after a single or, most often, multiple habituation sessions to a new environment (Bolivar, 2009). Habituation is key for behavioural studies, as the rodent must be as comfortable as possible with the presence of a human investigator, especially when the investigator needs to be present in the room to apply a stimulus (e.g. von Frey filament or Hargreaves tests). This is particularly pertinent given that ‘prey’ species, such as rodents, may mask signs of pain in the presence of a perceived ‘predator’ (Carbone, 2020).

Stress or anxiety during testing is another factor that might influence animal behaviour. For example, animal handling can induce stress that impairs test performance by suppressing exploratory behaviour and may lead to stress-induced analgesia (Gouveia and Hurst, 2017). Specifically, the use of tail handling creates a substantial interference with test responses. Tail-handled animals fail to perform free exploration of the test arena or pay sufficient attention to urine test stimuli. By contrast, non-aversive handling methods (e.g. tunnel handling or cupping mice without restraint on the open hand) rescue normal performance, indicating that animal handling has a direct impact on behaviour (Gouveia and Hurst, 2017). Handling-induced stress has also been identified as one of the causes of failure in replicating phenotypes within and between experiments (Crabbe *et al.*, 1999; Mandillo *et al.*, 2008). Therefore, aversive handling methods should be avoided to reduce confounding anxiety-related behaviours. Another factor that can induce stress to influence behavioural testing is experimenter sex (Sorge *et al.*, 2014). Male-related stimuli cause a robust physiological stress response that results in stress-induced analgesia in rodents. Thus, exposure of rats and mice to male, but not female experimenters, produces pain inhibition.

Finally, training by an experienced investigator is necessary to acquire consistent data (Bespalov and Steckler, 2018). For instance, in the mouse grimace scale, experienced investigators produce scores of higher consistencies with an accuracy of >97% compared to 72% of inexperienced investigators (Langford *et al.*, 2010). Inexperienced investigators, moreover, produce higher scores of mouse images than those originally predicted (Hohlbaum *et al.*, 2020; Whittaker *et al.*, 2021). Therefore, new investigators must be trained by experienced personnel to be considered apt and competent in performing the behavioural assays described here.

#### Deidentification, randomization, and avoidance of bias

In adequately designed animal studies, any sources of difference except for the treatment or intervention should be minimized. Randomization of animals is essential to ensure that any remaining differences (e.g. any variable that contributes to data ‘noise’ such as food, water, body size, environment, cage effects, and social hierarchy) are spread among all groups with equal probability, as well as minimizing any potential bias. Thus, when possible, ‘control’ groups should not be separated from the experiment, and ‘historical controls’ are not recommended, as each individual experiment will have its own sources of variation (Bespalov *et al.*, 2021). Investigators should be blinded to the allocated treatment or condition of the experimental group(s) being examined by deidentifying and randomizing each animal (Festing *et al.*, 2016). This extends from the behavioural testing itself throughout data analysis, as there will inevitably be outlier responses (as universally observed in biological studies). Further mitigation of bias should also be considered by using concealed allocation where, for example, the surgeon does not choose which animal to operate on, but the choice is randomly assigned prior to surgery. There is substantial evidence demonstrating that failure to blind investigators can introduce significant bias to the study by differentially testing and/or scoring behaviour based on animal grouping and their view of the experimental hypotheses. This, in turn, reduces reproducibility between studies and can increase the number of animals required to obtain statistically valid results (Bespalov *et al.*, 2020).

Where surgical methods are used to induce endometriosis-like lesions, it is also important to include sham animals to aid with blinding and mitigate any inherent nerve pathway changes associated with injury. For example, incision in the skin and deeper tissue can cause an acute increase in some pain-associated animal behaviours as well as spontaneous activity in peripheral and central neurones. These observations suggest that surgical procedures might influence pain and neuronal activity (Xu and Brennan, 2009, 2010). However, in other animal studies, surgery does not impact pain-associated behaviours; for example, ovariectomy induces a chronic increase in pain-related measures, whereas sham (ovary ligated but not removed) and intact rats do not (Li *et al.*, 2014). Further, in rats, endometriosis induces a chronic increase in pain-associated behaviours that is not seen in sham or intact animals (McAllister *et al.* 2009; Alvarez *et al.*, 2014). However, sham surgery after endometriosis, when its associated pain is established and stabilized, can exacerbate pain-associated behaviour (McAllister *et al.* 2012). Thus, when feasible, including sham animals as well as intact (naïve) animals to control for the effects of surgery and anaesthesia is recommended (McAllister *et al.*, 2009). For further recommendations for minimizing bias in preclinical studies of pain, see the findings of the PPRECISE pain consortium (Andrews *et al.*, 2016).

#### Strain of experimental animals

Most studies of EAP are conducted in either rats or mice. Among mice, C57BL/6 is the most common strain used. An analysis of mouse strain and pain studies published from 1980 to 2020 revealed that more than half of the published studies were in C57BL/6 mice (Sadler *et al.*, 2022). While C57BL/6 mice remain the top choice for studies of pain-associated behaviour, particularly because most genetically modified lines are bred on this background, FVB/N and BALB/c mice are also frequently used. It has been suggested that the use of outbred strains (such as CD1 and Swiss mice) might increase the translatability of preclinical pain experiments by better reflecting the heterogeneity of patients with chronic pain (Tuttle *et al.*, 2020). Nonetheless, given that most genetically modified lines are bred on the C57BL/6 background, this mouse strain should be preferred when studying mechanisms related to EAP.

Mouse strain choice might also impact observable behaviours in models of endometriosis. For instance, changes in paw weight distribution using the dynamic weight-bearing instrument have been reported for BALB/c (Tapmeier *et al.*, 2021), while for C57BL/6 mice, no changes were observed (Fattori *et al.*, 2020). Mouse strain may also impact normal locomotive behavioural activity, which may make them more/less suitable for evaluation in non-evoked tests. For example, a recent study reported that mice from different genetic background strains, including C57BL/6 and BALB/c, exhibited varying behavioural patterns when assessed for sociability/novelty, memory function, and negative behaviours like despair and stress calls (Sultana *et al.*, 2019); all of which may be relevant to studies of EAP. They noted the widely used C57BL/6J mice tended to have different characteristics from other strains. Nonetheless, changes in normal exploratory and locomotor activities can be reduced with proper habituation (see section above) and, if possible, by comparing behaviour from the same mouse before and after the intervention.

In rats, most endometriosis studies have used the outbred albino Sprague-Dawley strain due to their docility and ease of handling. Additionally, these rats have predictable, easily maintained, and monitored oestrous cycles; there is considerable background information about autonomic and hormonal control of their pelvic viscera and reproductive behaviour; and much is known about the constituent differences in reproductive endocrinology between the rat and human. While less common, Wistar rats (also outbred) are also used. Less frequently, inbred strains such as the WAG/Rih are used. It is notable, however, that there are differences in visceral pain between laboratory rat strains, which can be relevant for the study of associated intestinal co-morbidities, including irritable bowel (O’Malley *et al.*, 2014). Further, of relevance to EAP, the efficacy of analgesics may differ between rat strains, emphasizing the importance of using multiple strains to enhance translatability to patients (Hestehave *et al.*, 2019).

#### Hormonal status of experimental animals

The reproductive (oestrous) cycle in female rodents represents a series of biological timepoints defined by circulating levels of the sex hormones oestrogen and progesterone. In adult, non-pregnant rodents, a complete oestrous cycle typically lasts 4–5 days and is comprised of four stages: proestrus (pre-ovulation), oestrus (ovulation), metestrus (± embryo implantation), and dioestrus (reset of the uterine lining). Since steroid hormone receptors are expressed throughout peripheral and central nerve pathways relevant to behaviour, it is necessary to consider if and when oestrous cycle should be factored into a behavioural testing repertoire.

Throughout the literature, there is confounding evidence on whether oestrous cycle stage can influence rodent behavioural outcomes. Such measures have included emotion-related (e.g. anxiety), social, locomotor, reward-driven, and cognitive (e.g. memory) behaviours (Rocks and Kundakovic, 2023), but not those involving novel objects (e.g. open field, elevated plus maze) (Chari *et al.*, 2020). In studies comparing sex differences in behavioural responses between male and female mice, the oestrous cycle does not necessarily impose additional variability onto experiments (Mogil and Chanda, 2005). Similarly, a recent study demonstrated that each female mouse exhibits a characteristic pattern of exploration that uniquely identifies it as an individual across many experimental sessions, independent of the oestrous state (Levy *et al.*, 2023). In addition, rodent oestrous cycles differ from human menstrual cycles, which can complicate the extrapolation of sex hormone-mediated effects between these species (Fillingim and Ness, 2000). Nevertheless, endometriosis is well-established as an oestrogen-dependent condition and, in experimental models of endometriosis, oestrous cycle stage can impact endometriosis-like lesion characteristics, contents (e.g. NGF and VEGF levels), and nerve density (Zhang *et al.*, 2008; Dodds *et al.*, 2017). Moreover, cyclical fluctuations in pain-associated behaviours have been demonstrated in mouse and rat models of endometriosis (Cason *et al.*, 2003; Nagabukuro and Berkley, 2007; McAllister *et al.* 2009; Dmitrieva *et al.*, 2012; Escudero-Lara *et al.*, 2021). Furthermore, interruption of the oestrous cycle, by reproductive senescence or surgical ovariectomy, impacts EAP behaviours and lesion characteristics in rodent models of endometriosis (Rajkumar *et al.*, 1990; Berkley *et al.*, 2007). While lesion size may not necessarily correlate with pain intensity or duration, the condition under investigation is fundamentally hormonally regulated and warrants the rodent oestrous cycle to be an early and careful consideration during experimental design.

Vaginal cytology is one of the most common and reliable ways to determine oestrous cycle stage. It requires rodents to be handled and vaginal lavage acquired daily for at least 1 week depending on the experimental endpoints to be assessed according to the EPHect-EM-Homologous SOP 7 (Burns *et al.*, 2025). This procedure may induce stress, as vaginal smears collected twice daily for 10 days induced behavioural despair and anhedonia in Swiss albino mice (Varol *et al.*, 2022). Therefore, animals should be acclimated to the procedure, and vaginal lavage should be performed no more than once per day. Samples should always be collected at the same time each day to control for the various durations of the stages. If vaginal smears are collected alongside behavioural measures, samples should be obtained after testing to not impact the behaviour(s) being measured. Each study should also explicitly describe how oestrous cycle stages were defined. Whether oestrous cycle inclusion is appropriate for a given study will also depend on the types of behaviours to be examined, as some pain-associated behaviours are influenced by oestrous stage while others are not (Lovick and Zangrossi, 2021). Power calculations for sample size are particularly important, to adequately support studies examining rodents in specific phases of oestrous and/or to mitigate any potential variability attributed to testing animals in different cycle stages.

#### Ethical considerations for method selection

Behavioural tests are experimental measures used to quantify pain and other subjective experiences in rodents. Their purpose is not to inflict pain or discomfort on the subject but rather to determine how their responses to sensory stimuli change under specific circumstances (e.g. experimental endometriosis). Since a key clinical feature of endometriosis is persistent, debilitating pelvic pain, the mechanisms underlying the development of such pain are currently a major line of inquiry. Hence, ethical clearance to test pain-associated and other ethologically relevant behaviours is critical to advance knowledge in the field.

All animal use in research must be carried out in strict accordance with international guidelines, such as those recommended by the International Association for the Study of Pain (IASP), as well as individual institutional animal welfare requirements. For both the induction of experimental endometriosis and to perform behavioural testing in rodents, a compelling case for their use must be made, such as that described above. Contingency plans must also be in place to minimize, as much as practicable, any unexpected adverse impacts on animal wellbeing, without compromising the experimental endpoints to be assessed. Use of non-steroidal anti-inflammatory drugs (NSAIDs) for analgesia is strongly discouraged, as inflammatory processes are central to the pathogenesis of endometriosis. If analgesia must be provided, the partial opioid receptor agonist, buprenorphine, is a suggested alternative that is less likely to interfere with lesion development. Tramadol applied by drinking water might be another option for post-surgical analgesia, but this treatment regime needs close control of animal drinking behaviour to avoid under-dosing (Evangelista-Vaz *et al.*, 2018).

Additional ethical considerations include the age of animals to be used (which should align with the clinical time-course of endometriosis; see Flurkey *et al.* (2007) for approximate lifespan equivalency between rodents and humans), the degree of animal habituation and handling (see section above), and the minimization of animal use. Power calculations during experimental design are again important in this context, to reduce unnecessarily large sample sizes or, conversely, animal wastage resulting from experiments that cannot be statistically valid (Schwab *et al.*, 2022).

#### Timeline of behavioural testing

There is significant variation in the literature regarding timing of behavioural testing following induction of experimental endometriosis. For example, compared to baseline (pre-induction), von Frey filament testing in mice has been performed from as early as 5–7 days post-induction (Fattori *et al.*, 2020; McAllister *et al.*, 2021), with other studies ranging between 21 and 40 days post-induction (Greaves *et al.*, 2017b; Forster *et al.*, 2019; Dorning *et al.*, 2021; Escudero-Lara *et al.*, 2021). An appropriate timeline for assessing behaviour in experimental models of endometriosis is thus another key factor to be considered depending on the study hypothesis. For investigations focussed on pain development in parallel to lesion formation, early behavioural testing post-induction could be justified. Alternatively, studies on persistent EAP (such as those evaluating novel therapeutics) might prefer to assess behaviour when lesions are known to be well established from around 2–4 weeks post-induction (subject to the model used) (Burns *et al.* 2025). The type(s) of behaviour under investigation is also important in terms of timing, with some presenting earlier post-induction (e.g. mechanical allodynia) than others (e.g. abdominal licking) in the same model (Fattori *et al.*, 2020).

#### Collection and analysis of nervous tissue

When studying mechanisms of EAP, the anatomical and molecular characteristics of associated nerve pathways may be examined in addition to behaviour. The neural mechanisms by which endometriosis may generate pain have been previously described (Coxon *et al.*, 2018; Maddern *et al.*, 2020; McNamara *et al.*, 2021). Typically, tissues studied include peripheral nerve fibres innervating endometriosis-like lesions (± adjacent structures) as well as their respective dorsal root ganglia (DRG), neuronal circuits in the spinal cord, and/or brain regions relevant to the experience of pain. Assessment of endometriosis-associated adaptations in neuronal signalling can therefore be highly varied in the region of interest, as well as in the distinct genes/proteins under investigation and the experimental tools used for their evaluation.

Lesion innervation is most often determined using immunohistochemical techniques on fixed specimens. Total nerve density can be examined using pan-neuronal markers, including protein gene product 9.5 (PGP9.5) and β-III tubulin (TUJ3). Identification of neuronal subpopulations, such as sensory and autonomic (sympathetic and parasympathetic) neurones, may be further visualized and quantified using cell type-specific markers. For sensory neurones, these include isolectin B4 (IB4) for non-peptidergic afferent fibres and calcitonin gene-related peptide (CGRP) or substance P (SP) for peptidergic afferent fibres. Markers for sensory receptors known to encode nociceptive information, such as transient receptor potential (TRP)V1 and TRPA1, can also be used, although clearer signals for these proteins may be obtained within neuronal cell bodies (see below). Sympathetic neurones are generally identified with markers for tyrosine hydroxylase, dopamine β-hydroxylase (DβH), and vesicular monoamine transporter 2 (VMAT2), whereas parasympathetic neurones are identified via the expression of choline acetyltransferase (ChAT), neuronal nitric oxide synthase (nNOS), and vasoactive intestinal peptide (VIP).

Spinal afferent (e.g. nociceptor) cell bodies within DRG can be both molecularly and functionally characterized. Techniques for the former may include immunohistochemistry or western blot, and the latter might require, for example, DRG dissociation with ratiometric calcium imaging or electrophysiological recordings. Since endometriosis-like lesions may form in varied locations throughout the abdomen (particularly when utilizing peritoneal injection of endometrial tissue for lesion induction (Burns *et al.*, 2025), selecting the correct DRG levels in models of EAP is of critical importance. Where lesions are found in the upper abdomen (e.g. on the diaphragm or stomach), DRG spinal levels up to approximately thoracic T7 may be examined, whereas for those in the lower abdomen (e.g. lesions on the distal colon or bladder), this may include DRG down to approximately sacral S2 (Dmitrieva *et al.*, 2010; Dodds *et al.*, 2019). In all cases, both banks of DRG on either side of the spinal cord should be taken for analysis. In addition to spinal afferents, coeliac ganglia (McAllister *et al.*, 2012; Berkley and Dmitrieva, 2013) and vagal afferent cell bodies in nodose ganglia (Hao *et al.*, 2021, 2022) may be considered, given recent evidence suggesting involvement of the autonomic nervous system in endometriosis-like lesion development. Non-neuronal cells of the peripheral nervous system, such as satellite glial cells and Schwann cells, can also contribute to pain (Ji *et al.*, 2016).

In the spinal cord, spinal afferents synapse with projection neurones in the dorsal horn, which convey incoming sensory information to the brain. The spinal dorsal horn is organized into different layers (laminae), and, typically, the superficial lamina I–II regions are where nociceptive nerve fibres terminate (D'Mello and Dickenson, 2008). Projection neurones from lamina I generally innervate the thalamus, periaqueductal grey (PAG), and parabrachial (PB) areas of the brain, and the rostral ventromedial medulla (RVM) of the brainstem (Todd, 2002). The deeper dorsal horn layers, laminae III–V, carry other sensory information related to pain and predominantly project to the thalamus. Sensory signals are then carried on to various cortical regions, including the somatosensory, insular, anterior cingulate, and prefrontal cortices, that integrate to form the pain experience (D'Mello and Dickenson, 2008). In addition to these key areas, projection neurone activity in the spinal cord can be modulated by descending (monoaminergic) signals from the brainstem and by excitatory (glutamatergic) and inhibitory (GABAergic) spinal interneurones. Moreover, non-neuronal cells of the CNS, such as glia (astrocytes and microglia), are well known to influence nociceptive transmission and the development of chronic pain. Recently, altered glial cell reactivity in the spinal cord and brain has been postulated to contribute to EAP (Dodds *et al.*, 2016, 2019; Bashir *et al.*, 2023; Castro *et al.*, 2024). Collectively, these neuronal (and associated) circuits may be interrogated by a diverse range of experimental techniques to deepen our understanding of pain mechanisms in endometriosis.

### Stimulus-evoked (reflexive) methods to assess pain

#### von Frey filaments

The von Frey test is the most used measure for mechanical allodynia (Minett *et al.*, 2011) and has been used in several studies evaluating EAP (Arosh *et al.*, 2015; Greaves *et al.*, 2017b; Forster *et al.*, 2019; Fattori *et al.*, 2020; McAllister *et al.*, 2021; Tejada *et al.*, 2022). In rodents, it consistently demonstrates that the presence of ectopic endometrial tissue produces mechanical allodynia both at the hind paw and abdomen. For manually applied filaments, rodents are placed in individual transparent boxes (but are unable to observe one another) on a raised mesh grid. The test consists of thin, calibrated, nylon filaments of different gauges/stiffness applied to the plantar surface of the hind paw or abdomen until there is a slight bend to determine the threshold that elicits a withdrawal response. This delivers a constant, pre-determined force to the surface of application. When the filaments are applied to the hind paw, a withdrawal response (i.e. a positive response to a filament) is considered as a sharp paw withdrawal, flick, lick, or shake during the stimulus or immediately after the filament is removed. For the abdomen, a positive response is considered a jump away from the filament or a retraction of the abdomen (e.g. arching towards the spine and away from the filament). In studies of EAP, application to the hind paw is considered a measure of ‘referred’ pain and reflective of cross-sensitization, whilst abdominal application is more representative of a local pain response. For abdominal von Frey, the area of application is key, and must not be close to any surgical incision site. A diagrammatic representation of the recommended area is provided in EPHect-EM-Pain SOP 2 (Supplementary File S1). Several different von Frey methods have been described based on the order of filament application, including the ‘up-down’, a simplified up-down method (SUDO), ‘ascending stimulus’, and ‘percent response’. All methods are reviewed in Deuis *et al.* (2017) and detailed in EPHect-EM-Pain SOP 2 (Supplementary File S1). In addition to the SOPs referenced throughout this manuscript, please also refer to and read in conjunction with the ‘Minimum Standard Documentation for Behavioural Testing’ (Supplementary Table S1), which highlights recommended training, habituation, and intervals between stimuli and tests, as well as the method of analysis for each behaviour.

Key considerations for the von Frey filament test include habituation, training of the investigator, avoiding confounding factors and bias, and species choice. Whilst the von Frey test allows recording of mechanical threshold in unrestrained animals, removing some handling-induced stress, rodents must be acclimatized to the investigator and new environment and to the procedure (Minett *et al.*, 2011). For habituation, it is advised that experiments are performed in an isolated room used exclusively for this behavioural test and away from direct light. If the room is too noisy or the rodents are directly illuminated, they will require an extended period to relax and become habituated to their new environment. Following acclimatization, rodents must be habituated to the test via exposure to non-noxious filaments. A detailed description of the habituation procedure is available in EPHect-EM-Pain SOP 1 (Supplementary File S1). Training and experience are essential to be able to discern ‘painful’ from ‘non-painful’ responses following application of the filament. Appropriate training requires mentoring over a variable timeframe (5–12 months) until the trainee can consistently replicate results from the mentor.

The von Frey filament test remains common amongst researchers in the broader pain field due to the quantifiable nature of data that are produced, as well as the ability to compare results across groups and studies. The test can be used on both mice and rats, although the filament sizes used for each species will differ. While less time-consuming, recordings made from both the electronic von Frey (automated probe) and electronic version of the filaments (human-applied probe) vary significantly from manually applied von Frey (Deuis *et al.*, 2017). This discrepancy could be a result of activation of different subsets of sensory neurones (Deuis *et al.*, 2017); however, proof-of-concept experiments to confirm this hypothesis are still lacking. It is important to highlight that although mechanical pressure recordings (obtained using electronic methods) are different from the manually applied von Frey filaments, both the electronic von Frey and electronic versions of the filaments show consistent measurements within experiments (Cunha *et al.*, 2004; Zylka *et al.*, 2008; Pan *et al.*, 2018) (EPHect-EM-Pain SOP 3; Supplementary File S1). The consensus is that von Frey filament testing remains the gold standard for measuring mechanical threshold.

#### Thermal hyperalgesia (Hargreaves and hot plate tests)

The hot plate test is based on the principle that rodents will produce behaviours such as paw licking, flinching, or jumping when placed onto a hot surface (Le Bars *et al.*, 2001; Deuis *et al.*, 2017) (EPHect-EM-Pain SOP 7; Supplementary File S1). The surface temperature for this test varies from 50 to 55°C. Prior to any treatment or manipulation, rodents typically exhibit a 10–25 s reaction time (latency) to display one of the behaviours during baseline measurements (latency will vary according to the surface temperature) (Le Bars *et al.*, 2001; Deuis *et al.*, 2017). A reduction in the latency time for producing such behaviours correlates with pain intensity (Le Bars *et al.*, 2001; Deuis *et al.*, 2017). A major drawback of the hot plate test is the learning component, where repeatedly tested naïve rats show a reduced latency to produce one of the pain-associated behaviours (Le Bars *et al.*, 2001). This may be overcome by performing single measurements or increasing the time between measurements.

The Hargreaves test also measures responses to noxious heat (EPHect-EM-Pain SOP 6; Supplementary File S1). It differs from the hot plate test (where heat contacts all four paws) in that the heat stimulus is directed at a single area of the body (e.g. the plantar surface of a paw). In this method, the time from onset of a thermal stimulus to paw withdrawal (in seconds) is measured by applying a radiant or infrared heat stimulus directed to the hind paw (Le Bars *et al.*, 2001; Deuis *et al.*, 2017). A heated glass surface is often used to minimize errors arising from heat sink effects, where the thermal stimulus is applied to the plantar surface of the paw through the glass plate, and the paw withdrawal latency is determined (Deuis *et al.*, 2017). Most commonly, the produced pain-associated behaviours are paw licking and flinching, and, similar to the hot plate test, rodents display one of these behaviours over a delayed period during baseline measurements. A reduction in the latency time for producing such behaviours correlates with pain intensity (Le Bars *et al.*, 2001; Deuis *et al.*, 2017). The Hargreaves test also requires animals to be acclimatized to the apparatus (typically 2–3 sessions of up to 30 min each over consecutive days), and habituation is recommended to reduce exploratory movement for an accurate determination of paw withdrawal latency.

Both the hot plate and Hargreaves tests have been used for EAP research in rats and mice (Hernandez *et al.*, 2015; Du *et al.*, 2017; Hernandez *et al.*, 2017; Fattori *et al.*, 2020; Cordaro *et al.*, 2021; McAllister *et al.*, 2021). In surgical models of endometriosis, response latency is decreased relative to respective baseline measurements and compared to sham animals when assessed by hot plate and Hargreaves tests (Castro *et al.*, 2021; McAllister *et al.*, 2021). In non-surgical mouse models of endometriosis, response latency is decreased compared to sham when assessed by hot plate (Cao *et al.*, 2019; Zheng *et al.*, 2023) but similar to sham when assessed by Hargreaves (Fattori *et al.*, 2020). This indicates that thermal hypersensitivity might be produced in a subset of endometriosis cases. While evoked responses to thermal stimuli are frequently used behavioural tests in rodent models of endometriosis, sensitivity to heat is rarely reported by individuals with endometriosis, whereas mechanical hyperalgesia is more common (Coxon *et al.*, 2021).

#### Thermal preference

The thermal gradient assay can be used to determine general discomfort in mice (Touska *et al.*, 2016; Alexandre *et al.*, 2017; Fattori *et al.*, 2020) (EPHect-EM-Pain SOP 11; Supplementary File S1). For this test, a continuous temperature gradient (7–50°C) is established along a metallic base plate where the mice are placed. After an exploration period (30 min), individual mice show a distinct preference for a given temperature, as observed by a higher time spent in that zone. This is interpreted as the most comfortable temperature range. This assay uses video recordings to measure behaviour in freely moving mice without the presence of a human investigator (Alexandre *et al.*, 2017; Fattori *et al.*, 2020). Pain-free (sham/naïve) mice often spend more time in temperatures around 27–36°C with a stronger preference for 34°C (Fattori *et al.*, 2020). However, mice with EAP exhibit a more dispersed pattern that ranges from 21 to 36°C with no single preferred temperature (Fattori *et al.*, 2020). Interestingly, this change in normal thermal selection is alleviated by clinically active drugs, such as letrozole and danazol, with a return to strong preferences for 34–36°C (Fattori *et al.*, 2020). Changes in thermal selection have also been demonstrated in other contexts and correlate with ‘pain’ (Alexandre *et al.*, 2017). This indicates that pain, including EAP, interferes with normal thermal selection and may be rescued by clinically active drugs.

An advantage of this assay is that the mouse is unrestrained and able to determine their own preference for temperature (most time spent in the comfortable temperature zone) in the absence of a human investigator. This partly fulfils the criteria for spontaneous pain measurements in rodents. Specific equipment is required for such testing, and minor changes to lighting in the room or noise might produce significant variability in the obtained measurements. Therefore, a cost–benefit analysis should be considered prior to using the thermal gradient assay in endometriosis research.

#### Visceromotor reflex

Balloon distention of hollow organs has been routinely performed to experimentally measure visceral sensitivity in laboratory animals since the 1980s. This procedure is based on observations that controlled organ distention in human subjects recapitulates the intensity and referral pattern of visceral pain (Ness and Gebhart, 1988), including vaginal distention (Farmer *et al.*, 2013). Balloon distention was originally developed to measure physiological responses to stimulation of gastrointestinal organs, including the colorectum (Ness and Gebhart, 1988), oesophagus (Qin *et al.*, 2004), stomach (Kozakai *et al.*, 2019), and, later, the urinary bladder (Ness *et al.*, 2001), albeit through direct distention without a balloon. While several measures are affected by increased organ distention, including heart rate, blood pressure, and respiration (Ness and Gebhart, 1988), the most frequently reported is the visceromotor reflex (VMR), or response, from the abdominal musculature. The VMR is typically measured through electromyographic (EMG) electrodes implanted either chronically or acutely in the abdominal musculature (Christianson and Gebhart, 2007). Upon distention, the muscles contract in an intensity-dependent manner that is temporally linked to the onset and offset of the stimulus. Plotting VMR against the applied pressure produces a stimulus–response function that is responsive to experimental manipulations that either decrease or increase noxious input from the distended organ.

Several groups have adapted VMR to measure vaginal sensitivity in both mice (Pierce *et al.*, 2014; Castro *et al.*, 2021) and rats (Nagabukuro and Berkley, 2007; Dmitrieva *et al.*, 2012) (EPHect-EM-Pain SOP 4; Supplementary File S1). It should be noted that pressures applied to the vagina are generally much higher than those applied to either the gastrointestinal organs or urinary bladder. This is due to differences in both the anatomy and physiology of these organ systems. Experimentally induced endometriosis increases VMR to vaginal distention, including surgical induction in rats (Nagabukuro and Berkley, 2007; Ge *et al.*, 2019; Davenport *et al.*, 2021), syngeneic induction in mice (Alali *et al.*, 2020), and autologous induction in mice (Castro *et al.*, 2021). While VMR under anaesthesia has been reported (Nagabukuro and Berkley, 2007), it is typically carried out in conscious animals. If light anaesthesia must be used, the presence of escape reflexes should be maintained; otherwise, the lack of VMR may be due to the depth of anaesthesia rather than a true diminished response. Conscious mice, and often rats, are kept restrained during the VMR recordings. This can cause a significant stress response that has been shown to augment later VMR recordings in a stress-induced vaginal hypersensitivity model (Pierce *et al.*, 2014). Habituation to restraint may be performed for several days prior to recording to diminish the stress response. It is important to report whether habituation was performed for proper interpretation and reproducibility by other researchers.

#### Escape response to vaginal distension

Escape response to vaginal distention (EPHect-EM-Pain SOP 5; Supplementary File S1) was designed to assess dyspareunia and vaginal hyperalgesia commonly associated with endometriosis (Stratton and Berkley, 2011). In this model, vaginal nociception is quantified by measuring the probability of an escape response to vaginal distention in conscious rats. Vaginal distention is produced by volumes of water delivered to the vaginal canal by an inflatable latex balloon (Bradshaw and Berkley, 2002; McAllister *et al.* 2009). In this model, from the training and testing chamber extends a hollow tube with light-emitting diodes and a photosensor. To perform an escape response, the rat is trained to extend her nose into the tube, which breaks the light beam to terminate a noxious stimulus (balloon deflates). Once trained, testing sessions consist of seven different distention volumes, and one sham volume delivered three times each in random order at ∼60-s intervals, to which the experimenter is blinded. The percent escape response is plotted as a function of balloon distention volume. This stimulus–response function, like VMR, is responsive to experimental manipulations that decrease or increase noxious input from the distended organ.

Experimentally induced endometriosis increases escape responses to vaginal distention, whereas a ‘sham’ procedure or long-term vaginal nociceptive assessment in naïve animals does not alter escape responses to vaginal distention (Cason *et al.*, 2003; McAllister *et al.*, 2009). One advantage of this model is that the development of endometriosis-associated vaginal hyperalgesia has been behaviourally characterized relative to the cysts and their sensory and sympathetic innervation (Berkley *et al.*, 2004; McAllister *et al.*, 2009, 2012, 2016). Additionally, the relationship between endometriosis-associated vaginal hyperalgesia and hormones, prostaglandins, growth factors, and cytokines, in this model, has been described (Cason *et al.*, 2003; Berkley *et al.*, 2007; Zhang *et al.*, 2008; McAllister *et al.*, 2016). This model has been used extensively to reveal several peripheral and central neural mechanisms by which endometriosis may cause pain, particularly the potential contribution of ectopic cyst innervation in pain (Berkley *et al.*, 2004; Stratton and Berkley, 2011).

The primary limitation of using the vaginal escape response to assess vaginal hyperalgesia is that it is labour-intensive and requires animal training. Additionally, specialized training of staff and customized equipment are needed. Endometriosis-associated vaginal hyperalgesia fluctuates significantly over the oestrous cycle, so it also is important to consider oestrous stage when designing experiments and reporting findings in this model (Cason *et al.*, 2003). Furthermore, rats used in these studies must have regular oestrous cycles, because irregular cycling and/or reproductive senescence influences oestradiol levels and therefore vaginal hyperalgesia (Berkley *et al.*, 2007).

#### Overall recommendation

As outlined in Table 1, assessment of mechanical hyperalgesia using von Frey filaments is the most economical and robust evoked measure of EAP-like behaviour in rodent models. Studies have consistently reported reduced mechanical withdrawal thresholds in mice with ectopic endometrial tissue. Training to use von Frey is arguably the most accessible, given that many groups investigating pain mechanisms in endometriosis have used this test. However, von Frey measurement as part of the reflex-based, evoked pain testing was broadly employed in the neuropathic pain field and revealed a high risk for lack of translation to the clinic by producing false positive results (Percie Du Sert and Rice, 2014; Mouraux *et al.*, 2021). There is contradictory evidence regarding thermal hyperalgesia in models of induced endometriosis and its relevance to patients. VMR and escape response tests are the most physiologically relevant evoked measures, as they more closely reflect visceral pain. However, their set-ups can be invasive and labour-intensive, and the tests require specialist equipment and expertise.

### Stimulus-independent (spontaneous) methods to assess pain

Abdominal contortions, squashing, or licking are classical behaviours that are often used to interrogate the analgesic effect of drugs for abdominal pain. These behaviours are rarely reported in disease models; rather, injection of different chemicals is required to produce them. Specifically for abdominal pain, intracolonic or intraperitoneal injection of acetic acid, capsaicin, allyl isothiocyanate (AITC), and others are usually required to induce abdominal contortions, squashing, or licking in rodents (Verri *et al.*, 2006; Tappe-Theodor and Kuner, 2014). Table 4 summarizes the most common stimuli used to produce spontaneous abdominal pain as well as their potential pain mechanism. While useful for understanding how a compound might potentially inhibit pain, such models are generally acute and do not adequately reproduce what is clinically observed in most patients with chronic abdominal pain (Tappe-Theodor and Kuner, 2014). In this section, we describe the three main spontaneous behaviours that have been identified in both surgical and non-surgical models of endometriosis in mice and rats. Pros and cons for each behaviour are discussed (and summarized in Table 2) as well as measurements that should be prioritized in endometriosis research when studying analgesic effects of compounds.

**Table 4.** Summary of common chemical agents used to induce spontaneous pain behaviours of the abdomen.

Behavioural outcome	Chemical agent	Potential mechanism	References
**Abdominal contortions**	Acetic acid	Partially by activation of TRPA1	(Vale *et al.*, 2004; Verri *et al.*, 2008; Magro *et al.*, 2013)
	Allyl isothiocyanate (AITC)	Activation of TRPA1	(Laird *et al.*, 2001, 2002; Gonzalez-Cano *et al.*, 2021)
	Capsaicin	Activation of TRPV1	(Laird *et al.*, 2001, 2002)
	Phenyl-p-benzoquinone (PBQ)	Unknown	(Verri *et al.*, 2008; Magro *et al.*, 2013)
	Potassium superoxide (KO_2_)	Induction of oxidative stress by increasing superoxide anion	(Fattori *et al.*, 2015; Maioli *et al.*, 2015; Yamacita-Borin *et al.*, 2015; Bernardy *et al.*, 2017)
	Zymosan	Partially by activation of TLR2	(Vale *et al.*, 2003, 2004)
**Abdominal squashing**	AITC	Activation of TRPA1	(Laird *et al.*, 2001, 2002; Gonzalez-Cano *et al.*, 2021)
**Abdominal licking**	Capsaicin	Activation of TRPV1	(Laird *et al.*, 2001, 2002)

#### Abdominal squashing, contortions, and licking

Abdominal squashing is defined as the number of times the animal presses the lower abdominal region against the floor (EPHect-EM-Pain SOP 10; Supplementary File S1). Abdominal contortions are also referred to as abdominal writhing and are the most commonly used behaviour to study abdominal pain in elicited models. This measure is classically characterized as a contraction of the abdominal muscle together with stretching of hind limbs. However, it can also be characterized as a contraction of the oblique musculature with inward movement of the appropriate ipsilateral hindlimb (EPHect-EM-Pain SOP 9; Supplementary File S1). Both squashing and contortions are quantified as bouts in a defined period. Finally, abdominal licking is defined as a grooming behaviour performed with the mouth that is confined to the abdominal region, i.e. the animal does not groom any other region before or after the behaviour. It can be quantified as bouts or time spent licking (EPHect-EM-Pain SOP 8; Supplementary File S1).

Abdominal squashing and contortions are observed in both rat (Lopopolo *et al.*, 2014; Di Paola *et al.*, 2016; Iuvone *et al.*, 2016) and mouse (Fattori *et al.*, 2020) models of experimental endometriosis. In rat models, similar to abdominal licking, scoring of these behaviours was part of a global score along with other measurements. Therefore, based on the studies cited here, it is hard to determine the extent to which each contributes to the overall score and whether sham/naïve rats would also display these behaviours. For contortions, upon intraperitoneal acetic acid injection, rats with endometriosis display more than double the number of abdominal contortions when compared to the sham-operated and non-endometriosis rats (no contortions observed in either control group) injected with acetic acid (Mvondo *et al.*, 2017; Pereira *et al.*, 2019). This indicates that endometriosis might sensitize animals to display more pain-associated behaviours following noxious irritants such as acetic acid. However, because abdominal contortions have been described in the absence of a second stimulus for rat models, we do not recommend using acetic acid to further increase the number of abdominal contortions. In the mouse model, while endometriosis mice show a time-dependent increase in abdominal squashing and contortions, sham mice do not display this behaviour (Fattori *et al.*, 2020), indicating that abdominal squashing was observed as a pain behaviour associated with endometriosis.

Abdominal licking is observed in surgical and non-surgical models of endometriosis, indicating that it is a conserved and likely relevant behaviour (Lopopolo *et al.*, 2014; Di Paola *et al.*, 2016; Iuvone *et al.*, 2016; Greaves *et al.*, 2017b; Forster *et al.*, 2019; Fattori *et al.*, 2020; McAllister *et al.*, 2021). In rat models, abdominal licking was reported as part of a global score along with other spontaneous behaviours such as ‘humpbacked’ position, stretching of the body with raised abdomen, and others (Lopopolo *et al.*, 2014; Di Paola *et al.*, 2016; Iuvone *et al.*, 2016). An arbitrary scale was attributed to each of these behaviours, and the sum of the duration of all behaviours was plotted. Therefore, while observed in rats, based on the studies cited here, it is hard to determine the extent to which it contributes to the overall score. Abdominal licking is a relatively simple behaviour to score, although it can be confused with normal grooming and be easily mislabelled if done by a non-trained investigator. This could, therefore, result in false-positive scorings. Moreover, this behaviour is also observed in sham/naïve mice, which indicates that it is not exclusively observed during pain. And, depending on the instrument/device used, it might be challenging to observe the abdominal licking.

Recent papers demonstrate that some behaviours, such as squashing, contortions, and licking of different body parts, are easily visualized if recorded using a camera that looks up through a transparent platform or floor, known as ‘bottom-up video’ (Bohnslav *et al.*, 2021; Zhang *et al.*, 2022). While such devices might facilitate its quantification, we recognize that these might not be available at all institutions, and analysis of abdominal licking in particular may need to be performed live. This may also increase the likelihood of less accurate scoring due to the inclusion of false positives or missed bouts, or because the animal suppresses abdominal licking due to the presence of the observer.

#### Locomotor/gait activity (CatWalk/DigiGait) and dynamic weight bearing

Gait or relieving posture analysis (dynamic weight bearing) has been used to interrogate pain or discomfort in different pain models, including peritonitis (Gruen *et al.*, 2014; Quadros *et al.*, 2015; Laux-Biehlmann *et al.*, 2016) and endometriosis (Tapmeier *et al.*, 2021). Commercially available instruments such as the DigiGait**™** (Mouse Specifics) or CatWalk**™** (Noldus Information Technology) use visible light to illuminate the paws for gait analysis in rodents. Some motion-related read-outs, such as coordination (that involves the swing phase of gait), paw angle, or weight distribution, may provide important information during rheumatic or neuropathic pain models (Quadros *et al.*, 2015; Xu *et al.*, 2019; Abeyratne *et al.*, 2021). These instruments, however, require animals to perform a forced or trained walking task and, similar to the Dynamic Weight Bearing device (Bioseb), they rely heavily on manual identification of paws. While these systems provide detailed quantification of certain gait features, they only capture brief snapshots of behaviour immediately after human handling (Dorman *et al.*, 2014; Deuis *et al.*, 2017; Xu *et al.*, 2019). Specifically for abdominal pain, the Dynamic Weight Bearing device detects weight distribution towards the front paw. However, to achieve this shift, injection of lipopolysaccharide (Gruen *et al.*, 2014) or zymosan (Laux-Biehlmann *et al.*, 2016) was required.

In endometriosis models, changes in weight distribution have been only reported in BALB/c mice (Tapmeier *et al.*, 2021), while for C57BL/6 mice, no changes were observed (Fattori *et al.*, 2020). Even though the cited systems capture a rich repertoire of motion-related behavioural readouts, it is not clear whether the outcome measures are relevant to endometriosis-related pain compared with grooming/licking, standing/rearing, or squashing of the abdomen, for example. Recent papers describe devices such as Blackbox or Inferred Behavior Observation Box (iBob) that apply bottom-up video recording in the dark (with a near-infrared camera) for long periods (Bohnslav *et al.*, 2021; Zhang *et al.*, 2022). These devices allow a better identification and visualization of different behaviours such as grooming and paw licking in freely moving animals. They also allow a combination of customized/open-source machine learning analytics to interrogate rodent behaviours (Bohnslav *et al.*, 2021; Zhang *et al.*, 2022). Altogether, the application of machine learning approaches with bottom-up recording of a freely moving mouse in the dark may result in improved performance for the analysis of important pain-associated behaviours for endometriosis research. However, the cost–benefit ratio must be considered for these devices as well.

#### Overall recommendation

Spontaneous pain behaviours such as abdominal contortions, squashing, and licking are present in both rat and mouse endometriosis models. While bottom-up video recording may facilitate their quantification, this is not required. Abdominal licking is present in both endometriosis and naïve/sham animals, and scoring is more difficult to perform without extensive training and use of appropriate ‘bottom-up’ video systems. Live scoring of abdominal licking is not recommended, as it may result in false-positive scoring. On the other hand, abdominal contortions and squashing are easily distinguished from any other behaviour in rodents and they are not observed in sham/naïve animals. We therefore recommend quantification of abdominal contortions and squashing without additional triggers (e.g. injection of acetic acid) to be among the top priorities for behavioural assessment of pain in models of endometriosis, as both spontaneous pain behaviours are expressed in rat and mouse models.

Current instruments to measure gait analysis often rely on heavy manual identification of paws with detailed analysis of gait features immediately after human handling. Moreover, changes in weight distribution during endometriosis appear to be strain-specific (with BALB/c but not C57BL/6 mice demonstrating this behaviour in association with the presence of endometriosis). Importantly, differently from models of rheumatic diseases or neuropathic pain, it is not clear the extent to which gait changes are relevant to EAP. Therefore, the cost–benefit ratio and disease relevance must be considered for these assays.

### Ethological behaviour assessment

Non-essential or elective behaviours are promising behavioural measures of pain that can be objectively quantified under ethologically relevant conditions. These natural, spontaneous, and often complex home cage behaviours include grooming, socializing, and nest building (Jirkof, 2014). Data are increasingly demonstrating that elective behaviours in rodents are indicators of wellbeing, are frequently impacted in poor health and painful conditions, and may be a more clinically relevant measure of pain (Tappe-Theodor *et al.*, 2019; Zhang *et al.*, 2021). The behavioural tests discussed below are summarized in Table 3.

#### Facial grimace

The facial grimace scale is a coding system consisting of five facial features perceived by human facial pain expression experts as potentially reliable indices of pain (Langford *et al.*, 2010). These facial features (or action units) are orbital tightening, nose bulge, cheek bulge, ear position, and whisker change and are scored as 0–2 (not present, moderately visible, or severe, respectively).

The mouse grimace scale (MGS) has been used to assess at least 14 commonly used preclinical pain models, which have revealed significant changes from baseline in some assays but not others. Specifically, the assays that involve injection of noxious stimuli (e.g. acetic acid, formalin, zymosan) produced a severe response on the three-point scale, as did acute hind paw incision and laparotomy. Noxious stimuli of moderate duration (10 min–4 h) were most likely to be associated with grimace features and significant response. However, the neuropathic models, chronic constriction injury, and spared nerve injury (measured at Days 1, 7, and 14 post-operation) were not associated with severe grimace. The authors suggest that the specificity of the ‘pain face’ to noxious stimuli of moderate duration compared to neuropathic models may reflect differences in the affective component of prolonged visceral neuropathic pain (Langford *et al.*, 2010). More recently, however, the MGS has been used to detect pain of a longer duration (up to a month post initial insult) in several other models (Whittaker *et al.*, 2021). Of particular importance is the use of a high-quality camera to obtain clear recordings of the mouse grimace, and the original authors have developed an automated system using artificial intelligence for rats and, more recently, mice (McCoy *et al.*, 2024). Thus far, the facial grimace scale has not been used as an outcome measure for experimentally induced endometriosis, but given the chronic and visceral nature of EAP, this assessment may not be particularly useful. Additionally, the specific training and specialized equipment required to produce consistent results are another potential disadvantage.

#### Cage lid hanging

Studies using automated video tracking to assess a range of ethological behaviours have indicated that cage-lid hanging is reliably impacted by a range of different pain conditions of different durations. Cage-lid hanging is defined as ‘a mouse climbing onto the metal lid of their home cage and suspending themselves, upside down, off the floor’ (Zhang *et al.*, 2021). The authors who initially characterized cage-lid hanging have developed several methods to assess the behaviour, from a simple camcorder set up, through to an automated detection of cage-lid interaction that measures the capacitance of the cage lid (Zhang *et al.*, 2021). HCA systems (discussed below) can automatically detect rearing and climbing behaviours and have the potential to identify and quantify cage-lid hanging. Moreover, lid-hanging is decreased in mice with experimentally induced endometriosis (Tejada *et al.*, 2022), indicating that it can provide useful information for endometriosis research.

#### Assays related to thigmotaxis

##### Open field activity

The open field activity test measures exploratory and locomotor activity of a rodent in an open, well-lit arena and is widely used as an anxiety test by measuring the natural avoidance of rodents entering the centre quadrant of an open field. The test measures distance travelled (indicative of general locomotion) and time spent in the centre of an arena versus the periphery. Animal behaviour is recorded using a video camera in a darkened room, and measures include total distance moved, time spent moving, total number of rears, time in centre, and time in the periphery near walls. An increased amount of time spent in the centre indicates less anxiety. (EPHect-EM-Pain SOP 15; exploratory behaviour EPHect-EM-Pain SOP 16; Supplementary File S1).

##### Elevated plus/zero maze

The elevated plus maze is validated to assess anxiety-related behaviours and define the brain regions involved in generating this behaviour (Pellow *et al.*, 1985; Walf and Frye, 2007). The testing apparatus consists of four arms (50 × 10 cm and elevated 40 cm from the floor): two are open without walls and two are enclosed by 40-cm high walls. Rodents avoid open spaces/heights due to the anxiety or fear it provokes and thus spending more time in the closed arms (EPHect-EM-Pain SOP 17; Supplementary File S1). The rodent is placed in the middle of the maze, facing a closed arm. Behaviour in the maze is videotaped; time spent in the open/closed arms and the number of entries made by the rodent (all four paws) onto the open/closed arms are assessed. Increased anxiety of endometriosis C57BL/6 mice in comparison to sham animals was observed in an elevated plus maze test (Nunez-Badinez *et al.*, 2023). The distance travelled in the closed arms, or the number of closed arm entries, is the most accurate measure of locomotor activity in this test. The elevated plus maze has been tested in a preclinical model of endometriosis, which showed that mice with endometriosis spent more time in the closed arms, and consequently less time in the open arms of the maze compared to sham mice, without displaying any differences in the total number of entries into the open arms. Overall, this result indicates the presence of anxiety-related behaviours in this endometriosis model (Nunez-Badinez *et al.*, 2023).

The elevated zero maze is similar to the plus maze but does not have a centre zone and so eliminates any ambiguity this might cause (Shepherd *et al.*, 1994). The test measures unconditioned anxiety due to elevation and rodent aversion to open spaces (EPHect-EM-Pain SOP 18; Supplementary File S1). Again, the behaviours are recorded, and outcomes that are quantified include time spent in open/closed arms and number of entries into open/closed arms. An increased amount of time in open arms indicates less anxiety.

#### Burrowing

The burrowing test was initially studied and validated for the effect of nerve injury and paw inflammation in rats (Andrews *et al.*, 2012), and the number of pain models has expanded quite considerably since the original publication. Several classes of analgesics have been shown to reverse pain-model-induced burrowing deficits. There are some publications using burrowing in mouse models of pain, but overall, it appears to be less sensitive than for rats. A refined protocol has been published for rats by the European Union pain consortium, Europain (Wodarski *et al.*, 2016) (EPHect-EM-Pain SOP 13; Supplementary File S1). The outcome measure is the amount of gravel removed from the tube, and animals with inflammatory pain or nerve injury show reduced levels of burrowing, which are attenuated by analgesics. The equipment is cheap, as it consists of just one plastic tube per rat, big enough to hold 2.5 kg of gravel (5 mm in diameter). Training and testing take place in a home cage over a period of 1 week. Rats must be individually housed for the test but can be returned to their home cage colony between training/test sessions. Typically, one or two rats out of 20 will not burrow sufficiently enough prior to induction of pain and will need to be excluded from the testing.

#### Nest building

Several different methods of nest building assays using a variety of materials exist. However, cotton squares are the most frequently used (Aulehner *et al.*, 2022). The type of assessment and parameters varies and include time to integrate to nest test (TINT), nest consolidation, nest complexity, and percent material integrated, as well as duration of nest building activity. In post-surgical studies of pain, nest building was reported as a useful measure to assess the impact of analgesic drugs, with several studies indicating an impact of the drug on nest building (Aulehner *et al.*, 2022). Final evaluation of results must be done with animals still in their testing cage. Any attempt to remove animals from the testing cage to take photos free of ‘interfering’ mice or to better evaluate the number and quality of nests will modify the ‘empty space’ results, as animals will move cottons balls whilst trying to escape from being captured by the operator (EPHect-EM-Pain SOP 12; Supplementary File S1).

#### Home cage analysis

In-cage analysis systems are gaining in popularity because they allow longitudinal monitoring of groups of rodents over several days and detection of changes in disease symptoms. One system that has been used for behavioural studies in endometriosis models is the HCA system available from Actual Analytics [https://www.actualanalytics.com/products] (EPHect-EM-Pain SOP 14; Supplementary File S1). This system allows rodents to remain in social groups and collects data on natural behaviours from each individual during day and night. Furthermore, data analysis allows comparisons between pre- and post-model induction of locomotion, temperature, social interaction, and behaviours such as drinking and climbing, as well as circadian rhythm. Other advantages of the system are that no animal handling and no special training are required.

For successful HCA analysis, appropriate anatomical chip insertion is critical. Each individual rodent is implanted subcutaneously with a tracker chip, which causes momentary discomfort as it is ‘injected’ under the skin and allows monitoring both by video and by a detection system in the base of the cage (Redfern *et al.*, 2017). The implantation site is critical since it can produce unreliable readings in case of chip misplacement. Another important aspect is the amount of bedding in the cage. Excessive use of bedding interferes with chip detection by antennas, and HCA signals have an inverse correlation with bedding amount. Bedding depth must be kept to a maximum of 0.5 cm, as long as it does not compromize rodent comfort. Other aspects to consider for the use of this methodology are to use hardware equipment with sufficient computing and data storage capacities for the large amounts of raw data that these systems produce, as well as a software tool for automated data wrangling and analysis.

There have been several studies using the equipment which have demonstrated both the parameters it can measure over a long period of housing (Yip *et al.*, 2019) and its use for drug testing (Mitchell *et al.*, 2020). A recent paper reported the use of this method to evaluate a heterologous mouse model of endometriosis versus sham animals, providing a comparison to results of evoked (von Frey) tests (Tejada *et al.*, 2022). Significant differences between endometriosis and control mice were observed with von Frey filaments, spontaneous climbing, and drinking times.

In summary, longitudinal monitoring of rodent behaviours can be regarded as a new, improved method for the assessment of spontaneous behaviours in preclinical models of disease. Compared to the classic evoked pain methods, continuous monitoring provides several advantages, including minimum animal handling, recording of several behaviours at the same time, and allows for comparisons between pre- and post-model induction. The HCA systems can additionally monitor these behaviours separately for each individual within a cage, providing a further advantage against other commercially available systems. Despite these clear advantages, critical aspects to consider are to ensure correct insertion of microchip ID tags and to prepare an appropriate data management plan beforehand as well as automated tools for data analysis. The HCA systems have been tested in a preclinical model of endometriosis and have revealed insights on long-lasting changes in spontaneous behaviours such as the amount of time spent climbing and drinking in endometriosis-induced compared to sham mice, and these changes correlated with evoked mechanical pain in the abdominal area.

## Discussion

Here, we present recommendations for the design and execution of studies that evaluate the behaviour of rodents with experimental endometriosis as a surrogate outcome measure of EAP. We have described the different behavioural tests used in preclinical studies and investigations of pain mechanisms in endometriosis and the wider pain field, and produced consensus SOPs for the different tests. These resources were collected, developed, and agreed on by an internationally convened working group of experts in the field of experimental models and pain. We believe this resource will enable improved reproducibility in the EAP field, enhance the execution of cross-centre collaborations for preclinical testing, and, ultimately, improve the collection of robust data, with better translation of potential therapies through to the preclinical-to-clinical pipeline. Below, we discuss the suitability of different behavioural tests as relevant outcome measures for the evaluation of visceral pain and make overall recommendations for the selection of tests.

### The challenge of assessing pain in rodents

A solid definition of a clinically relevant pain model is one that phenocopies persistent pathological pain that is experienced by humans and that can also measure endpoints that can be seen in humans (Tappe-Theodor and Kuner, 2014; Deuis *et al.*, 2017). For example, even though heat hyperalgesia is rarely reported by patients with endometriosis (Coxon *et al.*, 2021), assays such as the Hargreaves and hot plate are often used as pain readouts in endometriosis models. In addition, while there is increasing evidence of widespread pain in endometriosis (Phan *et al.*, 2021; Till *et al.*, 2023), measurement of mechanical threshold in the paw might not be as relevant as in the abdomen, where most lesions reside. Because only a small fraction of individuals with endometriosis report pain distant from the main foci (abdominal region), measurement of mechanical threshold in the paw might not be as relevant as in the abdominal region. Therefore, improved attention to measuring more clinically relevant pain outcomes, alongside the discovery of disease biomarkers and molecular targets to aid patient stratification, may improve success rates of clinical trials for analgesic drugs directed at chronic pain (Fattori *et al.*, 2017; Yekkirala *et al.*, 2017; Tracey *et al.*, 2019).

Historically, measurement of spontaneous abdominal pain in rodents relied on the administration of different chemicals into the peritoneal cavity. These are associated with a wide range of time-limited modifications to behaviour that coincide with the anticipated painful experience (Sadler *et al.*, 2022). Such models, however, are generally acute and do not adequately reproduce what is clinically observed in most patients with chronic abdominal pain (Tappe-Theodor and Kuner, 2014). Spontaneous abdominal pain is the main complaint of those with endometriosis, yet this is rarely reported/measured in animal models of endometriosis. This might be because several of the spontaneous measurements can be either challenging for researchers to score or may be undetectable by the investigator. The latter is particularly common in cases where testing is being performed by an inexperienced investigator. We envisage that the resources developed for this project will enable more researchers to implement robust testing of spontaneous behaviours in experimental endometriosis. We acknowledge that some methods, such as home cage analysis, require specific equipment used for long recording periods, while others, such as abdominal licking, require proper instruments for investigator-independent scoring. In addition, in contrast to the von Frey or Hargreaves tests (which measure specific aspects of pain, such as mechanical and heat sensitivity, respectively), the modality related to spontaneous pain might lack mechanistic specificity. Unlike tests that measure sensory hypersensitivity, measurements of spontaneous pain, such as paw flinching, are difficult to correlate to a specific modality of pain (such as allodynia, movement-evoked pain, or the affective and motivational components of pain). This is because spontaneous pain behaviours might be a result of conjunction of actions, which limit mechanistic investigations. This may hinder data interpretation when investigating the mechanism of action for drugs. Therefore, the information provided by measurements of spontaneous pain is qualitatively different from that obtained by evoked measurements, especially in disease models. These factors might contribute to several investigators choosing evoked measurements of pain over spontaneous measurements.

Evaluation of more natural rodent behaviours during different chronic pain models is gaining increased traction in the field. Several ethological behaviours, such as burrowing, nest building, and cage lid hanging, are reduced during pain (Mogil, 2009; Sadler *et al.*, 2022). However, it is also important to mention that changes in these behaviours are not observed across all modalities of pain and, similarly, their reduction does not necessarily correlate to analgesia (Mogil, 2009; Sadler *et al.*, 2022). Although no direct correlation between pain and alterations in ethological behaviours can be drawn, they are useful for understanding overall animal wellbeing, and improvements in these measures following treatment with potential therapeutics may be more clinically relevant.

### Clinical relevance of rodent behavioural tests to the lived experiences of those with endometriosis

Since pain is both a sensory and emotional experience, assessment of only evoked responses to stimuli as a measure for pain may limit interpretation of results. Moreover, the pain experienced by endometriosis patients is highly variable with a wide spectrum of presentations. It is likely that the mechanisms generating these heterogeneous phenotypes of pain differ, and, therefore, a single preclinical model or measure may not reflect the full spectrum of the human presentation.

Most questionnaire assessments for patients with endometriosis describe the experience of pain or other relevant covariates, such as emotional wellbeing, cognitive processes (e.g. pain catastrophizing) or behavioural impacts (e.g. pain interference). In general, they do not allow inferences to be made about mechanisms underlying the pain except for a few specific tools, such as those assessing for a potential neuropathic component (e.g. painDETECT, DN4, SLANS) or suggestions of central sensitization (e.g. the Central Sensitisation Inventory). The use of psychophysical tests allows a more objective assessment of pain processing by combining specific noxious stimuli (e.g. thermal, mechanical) with participant reports of perceived sensations (e.g. pain intensity, unpleasantness). This method of assessing pain correlates well with some of the evoked tests used in preclinical models of endometriosis, as discussed above. As mentioned, the bodily location chosen for these evoked measures is important. Altered sensation in an area where clinical pain is perceived (e.g. abdomen) may reflect peripheral or central processes, whilst alterations at a more distal site (e.g. hand or foot) suggest widespread sensory involvement and thus central processes (Bajaj *et al.*, 2003; He *et al.*, 2010; Stratton *et al.*, 2015; Grundström *et al.*, 2019).

Normal daily life activities are also known to be dysfunctional in people with chronic pain, either as a cause or a consequence of their pain. For example, those with endometriosis often report depression and anxiety in addition to insomnia or poor sleep quality (Leone Roberti Maggiore *et al.*, 2017; Estes *et al.*, 2021). Therefore, measuring the impact of endometriosis on ethological rodent behaviours may be used to parallel the impact of pain on wellbeing, as well as tests that represent proxy measures of anxiety or other rodent activities (e.g. thigmotaxis assays, nesting, HCA, etc.). Such measures of natural rodent behaviour may provide more valuable clinical translatability for EAP, as the main types of pain symptoms reported by patients with endometriosis occur without external stimuli (Giudice, 2010; As-Sanie *et al.*, 2019; Maddern *et al.*, 2020).

### Summary and recommendations

In rodents, current methodologies to assess pain mostly focus on detecting stimulus-evoked responses from the animal. Stimulus-evoked responses correlate with some aspects of pain, such as withdrawal from a stimulus or learnt behavioural avoidance from a potentially painful situation. This type of response is only related to one symptomatic feature (allodynia) found in chronic pain patients who more often present with spontaneous, ongoing, or intermittent pain. Measurements of only evoked pain in preclinical models may not be effective when translating successes into new pain medications; thus, the consortium recommends that, following validation, multiple types of pain-related and/or parallel rodent behaviours that accompany pain (e.g. anxiety) should be quantified as surrogate outcome measures for EAP. Given the complexity and diversity of pain, a multidimensional approach to behavioural assessment may more closely reflect the lived experiences of those with endometriosis. Harmonized methods and documentation that have been compiled during this project will facilitate essential comparisons among studies, improve translational applicability, and provide a superior holistic view of animal (and thus human) wellbeing.

## Supplementary Material

gaaf023_Supplementary_Data

## Data Availability

No new data were generated or analysed in support of this research.

## Appendix: EPHect Experimental Models Working Group

Nick A. Andrews: Salk Institute for Biological Sciences, San Diego, CA, USA

Michael S. Anglesio: University of British Columbia, Vancouver, BC, Canada

Caroline B. Appleyard: Ponce Health Sciences University-Ponce Research Institute, Ponce, PR, USA

Joe Arosh: Texas A&M University, College Station, TX, USA

Christian M. Becker: University of Oxford, Oxford, UK

Kaylon L. Bruner-Tran: Vanderbilt University Medical Center, Nashville, TN, USA; World Endometriosis Research Foundation, London, UK

Katherine A. Burns: University of Cincinnati College of Medicine, Cincinnati, OH, USA

Ronald L. Chandler: Michigan State University College of Human Medicine, Grand Rapids, MI, USA

Julie A. Christianson: University of Kansas Medical Center, Kansas City, MO, USA

Fiona L. Cousins: Hudson Institute, Clayton, VIC, Australia; Obstetrics and Gynaecology, Monash University, Clayton, VIC, Australia

Kelsi N. Dodds: Flinders University, Adelaide, SA, Australia; University of Adelaide, Adelaide, SA, Australia

Victor Fattori: Harvard University, Cambridge, MA, USA

Asgi Fazleabas: Michigan State University, Grand Rapids, MI, USA

Caroline Gargett: Hudson Institute of Medical Research, Clayton, VIC, Australia

Juan S. Gnecco: Tufts University, Medford, MA, USA

Raul Gomez: INCLIVA Instituto de Investigación Sanitaria, Valencia, Spain; University of Valencia, Valencia, Spain

Martin Götte: University of Münster, Münster, Germany

Erin Greaves: University of Warwick, Warwick, UK; World Endometriosis Research Foundation, London, UK

Linda G. Griffith: Massachusetts Institute of Technology, Cambridge, MA, USA

Patrick G. Groothuis: Byondis BV, Nijmegen, The Netherlands

Ruth Grümmer: University of Duisburg-Essen, Essen, Germany

Sun-Wei Guo: Fudan University, Shanghai, China

Shannon M. Hawkins: Indiana University School of Medicine, Indianapolis, IN, USA

M. Louise Hull: The Robinson Research Institute, University of Adelaide, Adelaide, SA, Australia

Lone Hummelshoj: World Endometriosis Research Foundation, London, UK

Mark R. Hutchinson: University of Adelaide, Adelaide, SA, Australia

Mohamed Gamal Ibrahim: Team Kinderwunsch Oldenburg GbR MVZ, Oldenburg, Germany

Elizabeth E. Marr: Tufts University, Medford, MA, USA

Stacy L. McAllister: Emory University School of Medicine, Atlanta, GA, USA

Stacey A. Missmer: Harvard TH Chan School of Public Health, Boston, MA, USA; University of Michigan, Ann Arbour, MI, USA; World Endometriosis Research Foundation, London, UK

Jeffrey S. Mogil: McGill University, Montreal, QC, Canada

Jens Nagel: Merz Therapeutics, Frankfurt, Germany

Warren B. Nothnick: Kansas University Medical Center, Kansas City, MO, USA

Paulina Nunez-Badinez: Bayer AG, Wuppertal, Germany (until 23 March 2023)

Kevin G. Osteen: Vanderbilty University Medical Center, Nashville, TN, USA

Daniëlle Peterse: Boston Childrens Hospital, Harvard Medical School, Boston, MA, USA

Michael S. Rogers: Boston Childrens Hospital, Harvard Medical School, Boston, MA, USA

Andrea Romano: Maastricht University, Maastricht, The Netherlands

Philippa T.K. Saunders: University of Edinburgh, Edinburgh, UK

Miguel Ángel Tejada: University of Granada, Granada, Spain

Kathy L. Sharpe-Timms: University of Missouri School of Medicine, Columbia, MO, USA

Waldiceu A. Verri: State University of Londrina, Londrina, Brazil

Paola Viganó: Fondazione IRCCS Ca’ Granda Ospedale Maggiore Policlinico, Milano, Italy

Katy Vincent: University of Oxford, Oxford, UK
