# Supplementary material for: WERF Endometriosis Phenome and Biobanking Harmonisation Project for Experimental Models in Endometriosis Research (EPHect-EM-Pain): methods to assess pain behaviour in rodent models of endometriosis
Source: Mol Hum Reprod. 2025 Jul 9;31(3):gaaf023. doi: 10.1093/molehr/gaaf023 (PMC12237517; doi:10.1093/molehr/gaaf023)
Supplement: gaaf023_Supplementary_Data [file gaaf023_supplementary_data.zip › MHR-24-0372.R1 Supplementary info.pdf]

Supplementary Information

World Endometriosis Research Foundation EPHect Experimental Models for Endometriosis Research (EPHect-EM-Pain): methods to assess pain behaviour in rodent models of endometriosis

Kelsi N. Dodds, Victor Fattori, Nick A. Andrews, Caroline B. Appleyard, Julie A. Christianson, Raul Gomez, Stacy L. McAllister, Stacey A. Missmer, Jens Nagel, Paulina Nunez-Badinez, Michael S. Rogers, Philippa T.K. Saunders, Miguel A. Tejada, Katy Vincent, Lone Hummelshoj, Kaylon L. Bruner-Tran, Erin Greaves for the EPHect Experimental Models Working Group

Supplementary Figure S1: Timeline and framework of EPHect initiative

Supplementary Table S1. EPHect-EM-Pain Standard Operating Procedures (EPHect-EM-Pain SOPs): Minimum Standard Documentation for Behavioural Testing

Supplementary File S1: EPHect Standard Operating Procedure Experimental models: pain (provided as a separate file)

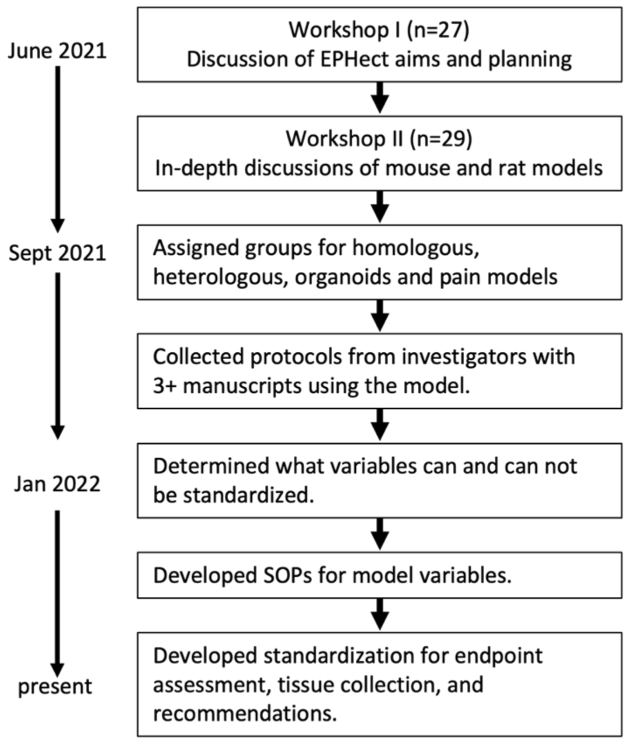

Supplementary Figure S1: Timeline and framework of EPHect initiative.

Supplementary Table S1. EPHect-EM-Pain Standard Operating Procedures (EPHect-EM-Pain SOPs): Minimum Standard Documentation for Behavioural Testing

**Behavioural test**	**Initial training / habituation**	**Habituation prior to each test**	**Intervals (between stimuli)**	**Intervals (between tests)**	**Method of analysis**
**von Frey filament**<br>*EPHect-EM-Pain SOP 2*	Minimum: 3 consecutive days, 120-minute sessions, stimulate with non-noxious stimuli<br>Standard: 4 consecutive days, 120-minute sessions, stimulate with non-noxious stimuli	Minimum: 45-60 minutes or until activity level is low<br>Standard: 120 minutes	Minimum:<br>Mice: 2 minutes between different filaments<br>Rats: 30 seconds between each filament, alternating left and right paws<br>Standard:<br>Mice: 2 minutes between different filaments<br>Rats: 60 seconds between each filament, alternating left and right paws	Minimum:<br>Mice: 1 day, do not test more than 1 body part on the same day<br>Rats: 1 day, measurements of different body parts can be done in the same day<br>Standard:<br>Mice: 7 days, do not test more than 1 body part on the same day<br>Rats: 5-7 days, measurements of different body parts can be done in the same day	Standard: Up-down method<br>Alternative: SUDO; percent response; ascending stimulus
**Electronic version of the von Frey filaments**<br>*EPHect-EM-Pain SOP 3A*	Minimum: 3 consecutive days, 60-minute sessions<br>Standard: 4 consecutive days, 60-minute sessions	Minimum: 30 minutes or until activity level is low<br>Standard: 45 minutes	Standard:<br>15 seconds between each application	Minimum: 30 minutes (acute models)<br>Standard: 2 hours for a maximum of 7 hours in one day (acute models) or daily (chronic models)	Standard: Expressed by delta (Δ) withdrawal threshold in grams, calculated by subtracting the mean measurements post-stimulus from baseline measurements

**Electronic von Frey**<br>*EPHect-EM-Pain SOP 3B*	Not required for rats	Minimum: 15 minutes<br>Standard: 30 minutes or until activity level is low	Standard:<br>Rats: 15 seconds between each stimulus, alternating left and right paws	Minimum: 1.5 hours<br>Standard: 1 day to several days	Standard: Expressed in grams
**Hargreaves**<br>*EPHect-EM-Pain SOP 6*	Minimum: 3 consecutive days,120-minute sessions<br>Standard: 4 consecutive days, 120-minute sessions	Minimum: 60 minutes or until activity level is low<br>Standard: 120 minutes	Standard: 2 minutes	Minimum: 2 hours, do not test more than 1 body part on the same day<br>Standard: 7 days, do not test more than 1 body part on the same day	Standard: Withdrawal latency in seconds<br>Alternative: Δ withdrawal threshold in seconds, calculated by subtracting the mean measurements post-stimulus from baseline measurements
**Hot plate**<br>*EPHect-EM-Pain SOP 7*	Not required but can be done for 10 minutes maximum on the day prior to testing or the day before testing	Not required but habituation to room recommended for 30 minutes prior to testing	N/A (single stimulus)	Minimum: 30-60 minutes (acute models) or 1 day (chronic models)<br>Standard: Every other day (chronic models)	Standard: Withdrawal latency in seconds<br>Alternative: Δ withdrawal threshold in seconds, calculated by subtracting the mean measurements post-stimulus from baseline measurements
**Thermal gradient**<br>*EPHect-EM-Pain SOP 11*	Not required	Standard: 30 minutes (during trial)	N/A	Minimum: Once a month<br>Standard: Last time point of behavioural measurements	Standard: Time spent in each temperature zone

**VMR to vaginal distension**<br>*EPHect-EM-Pain SOP 4*	Not required	Not required	Standard: 4 minutes	Minimum: 3 times per week<br>Standard: Every other day or 3 times per week for 6 months	Standard: Distension pressure in mmHg
**Escape response to vaginal distension**<br>*EPHect-EM-Pain SOP 5*	Minimum:<br>(4 weeks)<br>Box training: 3 days, 10-minute sessions<br>Tail pinch training: 4 sessions with 10 tail pinches<br>Vaginal training: 3 sessions of 10 large distention volumes<br>Standard:<br>(6 weeks)<br>Box training: 6 day, 10-minute sessions<br>Tail pinch training: 4 sessions with 10 tail pinches<br>Vaginal training: 4 sessions of 10 large distention volumes	Not required	Standard:<br>Tail pinch: 1-minute interval between tail pinches<br>Vaginal training: 1-minute interval between distention volumes with a maximum latency of 15 seconds<br>Testing: 1-minute interval (range 50-70 seconds) between distention volumes with a maximum latency of 15 seconds (one testing session is 1-hour duration)	Minimum: 3 times per week<br>Standard: Every other day or 3 times per week for 6 months	Standard: Percent escape response as a function of distention volume (mL)
**Abdominal squashing**<br>*EPHect-EM-Pain SOP 10*	Minimum: 3 consecutive days, 120-minute sessions<br>Standard: 4 consecutive days, 120-minute sessions	Minimum: 60 minutes or until activity level is low<br>Standard: 120 minutes	N/A	Minimum: 7 days<br>Standard: Last time point of behavioural measurements	Standard: Bouts per time<br>Alternative: Sum of all spontaneous behaviours

**Abdominal contortions**<br>*EPHect-EM-Pain SOP 9*	Minimum: 3 consecutive days, 120-minute sessions<br>Standard: 4 consecutive days, 120-minute sessions	Minimum: 30 minutes or until activity level is low<br>Standard: 60 minutes	N/A	Minimum: 7 days<br>Standard: Last time point of behavioural measurements	Standard: Bouts per time<br>Alternative: Sum of all spontaneous behaviours
**Abdominal licking**<br>*EPHect-EM-Pain SOP 8*	Minimum: 3 consecutive days, 30-minute sessions<br>Standard: 4 consecutive days, 60-minute sessions	Minimum: 30 minutes or until activity is low<br>Standard: 60 minutes	N/A	Minimum: 7 days<br>Standard: Last time point of behavioural measurements	Standard: Bouts per time<br>Alternative: Time spent licking the abdomen in seconds or sum of all spontaneous behaviours
**Open field**<br>*EPHect-EM-Pain SOP 15*	Not required	Minimum:<br>30 minutes on day before testing to the room<br>Standard:<br>30 minutes to the room	N/A (single stimulus)	At least 1 day between measurements	Standard: Total distance travelled, time spent in the centre of arena, time spent near walls of arena (periphery), number of rearings, number of entries to centre of arena, number of entries to periphery of arena, mean speed
**Exploratory behaviour**<br>*EPHect-EM-Pain SOP 16*	Not required	Standard: 30 minutes in test cage	N/A	Minimum:<br>2 consecutive days<br>Standard:<br>3 consecutive days	N/A

**EPM**<br>*EPHect-EM-Pain SOP 17*	Not required	Standard: 30 minutes to the room	N/A (single stimulus)	At least 1 day between measurements	Standard: Time spent and number of entries into both the open and closed arms
**EZM**<br>*EPHect-EM-Pain SOP 18*	Not required	Standard: 30 minutes to the room	N/A (single stimulus)	At least 1 day between measurements	Standard: Total distance travelled, time spent and number of entries into both the open and closed sections
**Burrowing**<br>*EPHect-EM-Pain SOP 13*	Mice: Can place empty tube in home cage overnight<br>Rats: 1 hour on Day 1 of training in pairs in test cage with an empty tube	Standard:<br>Mice: Not required<br>Rats: 60 minutes (30 minutes in home cage + 30 minutes in empty test cage)	N/A	At least 1 day between measurements	Tubes weighed before and after the burrowing test
**Nesting**<br>*EPHect-EM-Pain SOP 12*	Not required	Standard: 3 hours	N/A	At least 1 day between measurements	Standard: Nest quality score based on number of empty areas and quality of nest
**HCA**<br>*EPHect-EM-Pain SOP 14*	Not required	Not required	N/A	N/A	Bespoke software (e.g., Actual HCA Analyser™ software)

EPM: elevated plus maze; EZM: elevated zero maze; HCA: Home-cage analysis; N/A: not applicable; SUDO: simplified up and down; VMR: visceromotor reflex
