# Supplementary material for: WERF Endometriosis Phenome and Biobanking Harmonisation Project for Experimental Models in Endometriosis Research (EPHect-EM-Pain): methods to assess pain behaviour in rodent models of endometriosis
Source: Mol Hum Reprod. 2025 Jul 9;31(3):gaaf023. doi: 10.1093/molehr/gaaf023 (PMC12237517; doi:10.1093/molehr/gaaf023)
Supplement: gaaf023_Supplementary_Data [file gaaf023_supplementary_data.zip › MHR-24-0372-R1-SuppFileS1.pdf]

Kelsi N. Dodds, Victor Fattori, Nick A. Andrews, Caroline B. Appleyard, Julie A. Christianson, Raul Gomez, Stacy L. McAllister, Stacey A. Missmer, Jens Nagel, Paulina Nunez-Badinez, Michael S. Rogers, Philippa T.K. Saunders, Miguel A. Tejada, Katy Vincent, Lone Hummelshoj, Kaylon L. Bruner-Tran, and Erin Greaves for the EPHect Experimental Models Working Group.

TABLE of CONTENTS

Standardisation of pain-associated behavioural measurements in rodent models of endometriosis 2
SOP 1: General setup, habituation, and post-test procedures 4
SOP 2: von Frey filament test (abdomen and hind paw) 5
SOP 3a: Electronic version of the von Frey filaments (abdomen) 11
SOP 3b: Electronic von Frey test (hind paw; non-bending filament) 13
SOP 4: Visceromotor response (VMR) to vaginal distension 15
SOP 5: Escape response to vaginal distension 21
SOP 6: Hargreaves test (hind paw) 26
SOP 7: Hot plate test (hind paw) 29
SOP 8: Direct abdominal licking 31
SOP 9: Abdominal contortions (writhing) 34
SOP 10: Abdominal squashing 36
SOP 11: Thermal gradient 38
SOP 12: Nesting 40
SOP 13: Burrowing 42
SOP 14: Home-cage analysis (HCA) 44
SOP 15: Open field 47
SOP 16: Exploratory behaviour 49
SOP 17: Elevated plus maze 50
SOP 18: Elevated zero maze 52

Standardisation of pain-associated behavioural measurements in rodent models of endometriosis

Statement of Purpose

The purpose of this standard operating procedure (SOP) is to describe and recommend protocols for the most common tests used as surrogate measures for pain associated with endometriosis in rodents. EPHect's purpose in the publication of these procedures is the standardisation (wherever possible, without limiting scientific creativity and discovery) of endometriosis experimentation across institutions to improve comparability of data, experimental integrity, and provide the mechanisms that lead to "best fit models" aiming to recapitulate human disease aetiology.

Recommendations

We have categorised key variables and recommendations as follows:

1. Training: Individuals **must** have been trained by an experienced investigator to be considered competent in performing the procedures described herein. The validity of results obtained from behavioural phenotyping is largely dependent on training and methods of animal husbandry. It is imperative that individuals following these procedures are skilled and aware of the animal's welfare.
2. Pain measurements: Pain measurements for endometriosis can be categorized into stimulus-evoked/reflexive (e.g., von Frey, Hargreaves), stimulus-independent/spontaneous (e.g., abdominal contortions, direct abdominal licking), or those that align with ethological behaviours (e.g., nesting, burrowing). The former two types of behavioural measurements can be directly correlated with pain status while the latter may reflect the overall wellbeing of the animal. A combination of several different methods is recommended for robust testing and clinical relevance.
3. Anatomical region for stimulation (evoked measures only): von Frey (filament or its electronic version) can be applied to the abdomen (i.e., pelvic pain) or hind paw (i.e., referred pain). Hargreaves is often applied to the hind paw.
4. Strain: C57BL/6 mice (8-12 weeks at the start of the experiment) are the most commonly used mouse strain, with most transgenic mice available on this background, although other strains are used for behaviour testing e.g., BALB/c due to their more amenable nature. Sprague-Dawley rats (2 months old, 180-200g) are the most commonly used rat strain.
5. Experimental design: It is **essential** that ways to minimize bias are employed when carrying out the behavioural testing. Experimenters measuring behavioural responses should be blinded to experimental status of the animals. Other good practices include randomization of experimental groups, blinded allocation to treatment groups when such experiments are being conducted, and appropriately powered sample sizes should be used where the variance is known. Where it is not possible to use these procedures, it should be clearly stated in the manuscript or record book.

The following SOPs are included in the document:

- SOP 1: General setup, habituation, and post-test procedures

Stimulus-evoked (reflexive) tests

Mechanical hyperalgesia

- SOP 2: von Frey filament test (abdomen and hind paw)
- SOP 3A: Electronic version of the von Frey filaments (abdomen)
- SOP 3B: Electronic von Frey (hind paws)
- SOP 4: Visceromotor reflex (VMR) to vaginal distension

- SOP 5: Escape response to vaginal distension

Thermal hyperalgesia

- SOP 6: Hargreaves test
- SOP 7: Hot plate test

Stimulus-independent (spontaneous) tests

- SOP 8: Direct abdominal licking
- SOP 9: Abdominal contortions (writhing)
- SOP 10: Abdominal squashing

Ethological behaviour tests

- SOP 11: Thermal Gradient
- SOP 12: Nest building
- SOP 13: Burrowing
- SOP 14: Home cage analysis (HCA)
- SOP 15: Open field
- SOP 16: Exploratory behaviour
- SOP 17: Elevated plus maze
- SOP 18: Elevated zero maze

SOP 1: General setup, habituation, and post-test procedures

Supplies and equipment

- Non-toxic marker for tail marking identification
- Paper towels
- Disinfectant or 70% ethanol to sanitize equipment
- Acrylic chambers (where applicable)
- Wire grid floors (where applicable – e.g., von Frey testing)
- Other apparatus (e.g., heated glass plate)

PROTOCOL

A. Setup

1. Tail-mark each animal with a non-toxic marker. It is critical that rodents are measured in the same chamber as they were habituated (where possible), throughout the entire experiment.
2. Upon any assessment of welfare concerns, rodents may be excluded from testing.
3. Before behavioural testing, the investigator should carefully handle all the animals to ensure acclimatization to the investigator.
4. Acrylic cages, wire grid floor, heated glass plate (where applicable) etc., and table must be cleaned with 70% ethanol before and after experiments.
5. Place paper towel under wire floor grid (where applicable) etc. to facilitate cleaning (i.e., faeces and urine).

B. Habituation

1. In a quiet, temperature-controlled room, gently place animals in individual acrylic cages with wire grid floors (where applicable) etc. Mice are often habituated for 4 consecutive days. See specific SOPs and Minimum Standard Documentation for recommended duration of habituation.
2. After the habituation time, the investigator may return to the test room and sit silently for 5 minutes to assist in the habituation process, to ensure all animals are acclimatized to the investigator.
3. It is recommended that all phenotyping experimentation is conducted at approximately the same time of day as physiological and biochemical parameters can change over time.

C. Post-testing

1. Once behavioural testing is completed, gently return animals to their home cages.
2. Ensure all equipment is clean (i.e., wiped down with disinfectant and/or 70% ethanol).

SOP 2: von Frey filament test (abdomen and hind paw)

Purpose

Pain intensity to a mechanical stimulus (mechanical hyperalgesia) in the abdominal or paw regions can be measured using von Frey (monofilament) filaments. The tip of the von Frey fibre is pressed against the target body part at a right angle. As the filament tip is pressed into the target, the force of application increases until the fibre bends. After the fibre bends, continued pressure on the target area creates more bend in the filament, but not more force of application. This principle makes it possible for the investigator using a handheld probe to apply a reproducible force, within a wide tolerance, to the target area. A withdrawal to the filament is considered as a "positive" pain-associated response. The procedure here is described by Dorning *et al*., 2021, Fattori *et al*., 2020, and Greaves *et al*., 2017 and is routinely used in rodent models of endometriosis.

Relevance to humans

The von Frey filament test is the most common test used by researchers in the pain field. Surgical and non-surgical induction of endometriosis in rodents reduces the mechanical threshold in both abdominal and paw (extra-pelvic) regions, indicating pain due to mechanical stimuli. Von Frey filaments are also used in humans to assess sensitivity to mechanical stimuli. The Quantitative Sensory Testing (QST) is often applied in clinical therapeutic trials to measure sensory thresholds for pain, touch, and vibration upon von Frey filament stimulation. Like rodents, increased mechanical thresholds correlate to lower pain levels whereas reduced thresholds correlate to allodynia and/or hyperalgesia. In patients with endometriosis, studies using QST report significantly altered pain thresholds in pelvic and extra-pelvic locations (Coxon *et al*., 2023). Therefore, given the similarities between human and rodent responses to the filaments and the quantifiable nature of data that is produced, as well as the ability to compare results obtained amongst groups, the von Frey test remains as one the gold standards to assess mechanical pain.

Supplies and equipment

The following equipment list is specific for manual von Frey in <u>mice</u>. Only equipment directly used for carrying out von Frey testing are included. Additional items considered to be standard laboratory equipment and personal protection equipment (PPE) are not listed.

- von Frey filaments in the range of 0.02g-4g
  Note*: The final thicker filament required depends on body part targeted (abdomen/paw)*
- Acrylic chambers with wire grid floors

<u>Optional equipment</u>

- Up-Down Reader software (Gonzalez-Cano *et al*., 2018)
  Note: *Software varies accordingly to the User's operational system (Mac/Windows)*
- Recording templates for animal responses to stimulation
  Link to download abdominal von Frey template:
  https://sourceforge.net/projects/updownreader/files/updownreader_v2.0/data_sheets/mouse_visceral_template.pdf/download
  Link to download hind paw von Frey template:
  https://sourceforge.net/projects/updownreader/files/updownreader_v2.0/data_sheets/mouse_plantar_template.pdf/download

PROTOCOL

A. Set up, habituation, and overall procedure (Days -6, -5, -4, and -3)

1. SOP 1: General setup, habituation, and post-test procedures.
2. Set up the von Frey platform, such that it is elevated above the eye level of a seated person. The platform should be placed in an area that allows the person to move around all sides of the platform without impeded access (Figure 1).
3. Mice are habituated in 120-minute sessions during 4 consecutive days prior to the behavioural testing.
4. Investigator may stimulate the mice during the first habituations with "non-noxious" filaments 0.4g (abdomen) or 0.6g (hind paw). It is expected that by the end of the second habituation, mice will stop responding to "non-noxious" filaments.
4. During the first habituations, it is expected that some mice will respond to those filaments. Such responses are typically due to an unknown stimulus and are not pain related.
5. Improper habituation, animal handling, or excessive application of the filaments (either number of times or interval between applications) may result in exaggerated response resulting in false positive readings or difficulties at applying the fibres due to excessive animal locomotion/activity.
6. It is critical to avoid any damage to the filaments. In case of damage due to mouse biting or any other issue, a new filament must be obtained.
7. Do not, under any circumstances, cut the filament in an attempt to "fix" it.

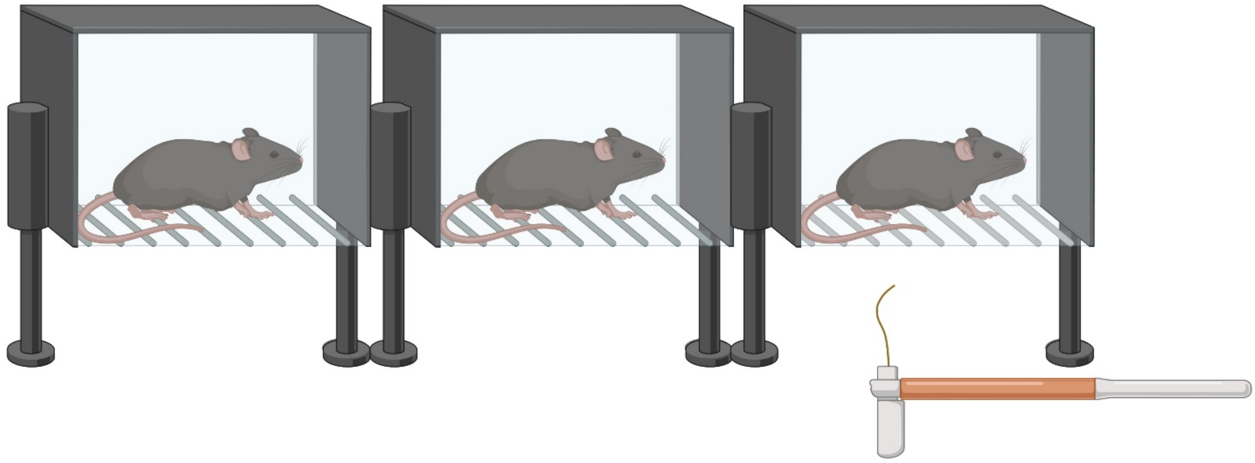

Figure 1. Example of von Frey testing set up. *Observation area with individual mice in acrylic cages placed on top of a wire floor grid. A filament is passed though the spaces on the bottom of the grid floor to stimulate the plantar surface of the hind paw. Image generated using BioRender.com.*

B. Baseline (Days -2 and -1) and post-endometriosis induction measurements

1. Starting filament and stimulation place will vary accordingly to targeted body part. To avoid exaggerated response to the filaments, they should be applied to the targeted body part with at least a two-minute interval between the different fibres.
2. Investigator needs to apply the fibre until it bends and hold it for around 1-2 seconds before withdrawal.
3. After testing is complete, return animals to home cages.

C. **Abdominal von Frey measurements**

1. It is critical that the von Frey filament is placed gently on the target body region before pressing into the region. The filament must not be "jabbed" or "poked" into the region.
2. Measurements should start with the 0.4g filament. Stimulation should be performed in the bladder region of the mouse (Figure 2). Maximum filament thickness is 2g.
3. Baseline measurements (Day 0) should be conducted (preferably) the two consecutive days before endometriosis induction, with the final value being the mean of the two-day measurements.
4. It is critical that activity levels are low during the assessment of this test. If after the acclimatization period mice are still active, acclimatization should be extended for an additional 30-60 minutes. Alternatively, another round of habituation is suggested.
5. Before stimulation is performed, mice need to have all four paws on the grid (i.e., mice must be still with no walking or rearing).
6. Care must be taken to avoid stimulation of genital organs (Figure 2).
7. It is not recommended to test more than one body part at a single day. If the investigator needs to measure both abdominal and hind paw mechanical threshold, one should do so on different days.

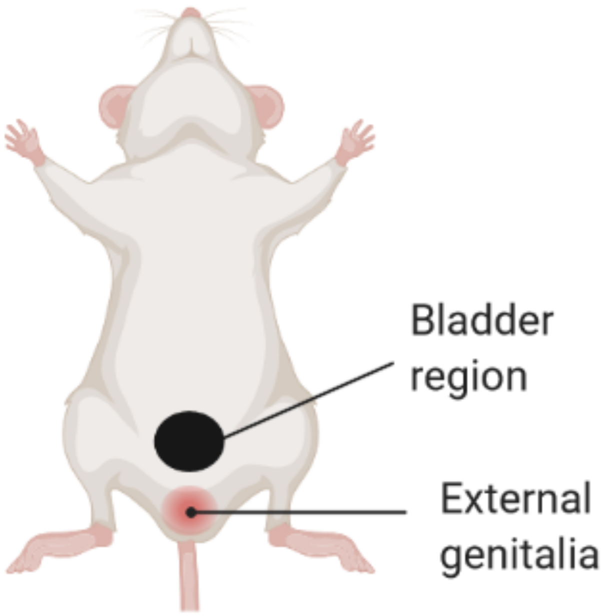

Figure 2. Mouse ventral view. *This figure shows the point of abdominal stimulation for von Frey fibres (black spot) over the bladder, and the external genitalia area (red spot) as the region to avoid. Image generated using BioRender.com.*

8. A positive response to the von Frey filament consists of a jump, paw flinch, or abdominal licking (at the site of stimulation).
9. When a positive response is observed, mark the response template with an "X" in the first box of the corresponding row of the table for that mouse, then move to testing the next thinner filament.
10. In the absence of response, an "O" is marked, then move to testing the next thicker filament.

__Note:__ Each mouse must be tested with the starting filament before proceeding with the subsequent thinner or thicker filament.

11. If using the Up-Down Reader software, the investigator needs to fill in the visceral template according to (Gonzalez-Cano *et al*., 2018).
12. Ensure all equipment is clean (i.e., wiped down with disinfectant and/or 70% ethanol) before testing another round of animals.

D. Hind paw von Frey measurements

1. Measurements should start with the 0.6g filament. Stimulation should be performed in the centre of the paw (Figure 3). Maximum filament thickness is 4g.
2. Follow the habituation and stimulation requirements described for abdominal von Frey testing, above.
3. If using the Up-Down Reader software, the investigator needs to fill in the paw template according to (Gonzalez-Cano *et al*., 2018).

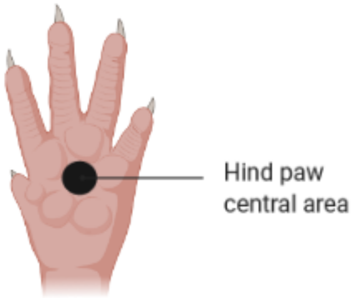

***Figure 3. Mouse hind paw view**. This figure shows the appropriate point of stimulation (black spot) for von Frey fibres to the paw. Image generated using BioRender.com.*

E. Post-testing

1. See SOP1: General setup, habituation, and post-test procedures.

F. Data plotting (if using the Up-Down Reader software)

<u>Determination of mechanical threshold</u>

1. Download the Up-Down Reader software (Gonzalez-Cano *et al*., 2018).
2. Scan the completed von Frey response sheets and upload them into the software.
3. Save the excel sheet generated by the software with the calculated mechanical thresholds (in grams). Of note, check for accuracy, as the software may misread some of the columns.
4. Alternatively, the 50% response threshold is interpolated using the formula: 50% g threshold = $10^{[X_f+\kappa\delta]}$ where:
   X_f = logarithmic value of the grams associated to the last von Frey filaments used during testing. In the example depicted in Figure 4, ***X_f=log 0.07*** (i.e., value annotated in row 5).
   k= value resulting from the tabular annotation of marks ("X" and "O") obtained. Values can be found as an Appendix of Chaplan et al., 1994 (Chaplan et al. 1994). In the example depicted in Figure 4, the tabular value for the XXOXXOO pattern sequence is =0.14.
   δ= A coefficient resulting from the calculation of the average mean difference (in log units) between different stimuli in the von Frey series. In the case of filaments employed for mice testing ***δ***= 0.24
   In the example below, the 50% response= $10^{[X_f+\kappa\delta}$ = $10^{(LOG(0.07)+0.144*0.24)}$ = 0.08g

	0,02	0,04	0,07	0,16	0,4	0,6	1	1,4	2
1			O	X	X				
1				X					
1			X						
1		O							
1			O						

Figure 4. Example Up-Down Reader software template following abdominal von Frey testing. *This mouse initially responded to a 0.4g filament, then stopped responding once the filament thickness reached 0.04-0.07g. A mechanical threshold of 0.08g was subsequently calculated.*

5. Alternatively, other methods for mechanical threshold determination such as "simplified up-down" (SUDO), "percent response", or "ascending stimulus" can be used.
6. For SUDO, differently of "Up and Down" method, a maximum of five von Frey filament presentations per test is used (Bonin et al., 2014). Similar to the "Up and Down", absence of response, an "O" is marked, then move to testing the next thicker filament. When a positive response is observed, mark the response template with an "X" in the first box of the corresponding row of the table for that mouse, then move to testing the next thinner filament (Bonin *et al*., 2014).
7. For the "percent response" method, filaments of ascending order of force are applied an equal number of times (usually 5–10 applications) regardless of response, and the number of positive responses to each filament is converted to a percent response (Ho Kim and Mo Chung, 1992).
8. For the "ascending stimulus" method, filaments ascending order of force are applied and the lowest pressure filament that induced a response in five out of ten applications in five out of ten applications is recorded as the mechanical threshold (Scholz *et al*., 2005).

REFERENCES

Bonin RP, Bories C, De Koninck Y. A simplified up-down method (SUDO) for measuring mechanical nociception in rodents using von Frey filaments. *Mol Pain*. *2014*; 10: 26.

Chaplan SR, Bach FW, Pogrel JW, Chung JM, Yaksh TL. Quantitative assessment of tactile allodynia in the rat paw. *J Neurosci Methods. 1994.* Jul;53(1):55-63.

Coxon L, Vollert J, Perro D, Lunde CE, Ferreira-Gomes J, Charrua A, Abreu-Mendes P, Krassowski M, Birch J, Meijlink J et al. Comprehensive quantitative sensory testing shows altered sensory function in women with chronic pelvic pain: results from the Translational Research in Pelvic Pain (TRiPP) Study. *Pain*. *2023*; 164(11): 2528-2539.

Dorning A, Dhami P, Panir K, Hogg C, Park E, Ferguson GD, Hargrove D, Karras J, Horne AW, Greaves E. Bioluminescent imaging in induced mouse models of endometriosis reveals differences in four model variations. *Dis Model Mech*. *2021*; 14(8): dmm049070

Fattori V, Franklin NS, Gonzalez-Cano R, Peterse D, Ghalali A, Madrian E, Verri WA Jr, Andrews N, Woolf CJ, Rogers MS. Nonsurgical mouse model of endometriosis-associated pain that responds to clinically active drugs. *Pain*. *2020*; 161(6): 1321-1331.

Greaves E, Horne AW, Jerina H, Mikolajczak M, Hilferty L, Mitchell R, Fleetwood-Walker SM, Saunders PTK. EP2 receptor antagonism reduces peripheral and central hyperalgesia in a preclinical mouse model of endometriosis. *Sci Rep*. *2017*; 7: 44169.

Gonzalez-Cano R, Boivin B, Bullock D, Cornelissen L, Andrews N, Costigan M. Up-Down Reader: An Open Source Program for Efficiently Processing 50% von Frey Thresholds. *Front Pharmacol*. *2018*; 9: 433.

Ho Kim S, Mo Chung J. An experimental model for peripheral neuropathy produced by segmental spinal nerve ligation in the rat. *Pain*. *1992*; 50(3): 355-363.

Scholz J, Broom DC, Youn DH, Mills CD, Kohno T, Suter MR, Moore KA, Decosterd I, Coggeshall RE, Woolf CJ. Blocking caspase activity prevents transsynaptic neuronal apoptosis and the loss of inhibition in lamina II of the dorsal horn after peripheral nerve injury. *J Neurosci*. *2005*; 25(32): 7317-7323.

SOP 3a: Electronic version of the von Frey filaments (abdomen)

Purpose
Similar to its manual counterpart, the electronic version of the von Frey filaments can be used to measure pain intensity in the abdomen or paw regions to a mechanical stimulus. It involves a human-applied electronic probe. This procedure is described by Colombo *et al*., 2018.

Relevance to humans: See SOP2.

Supplies and equipment

The following equipment list is specific for electronic version of the von Frey in mice. Only equipment directly used for carrying out von Frey testing are included. Additional items considered to be standard laboratory equipment and PPE are not listed.

- Electronic version of the von Frey filaments (e.g., cat #EFF 301; Insight instruments; Ribeirao Preto, SP, Brazil).
- von Frey polypropylene tip
  Note: *For abdominal stimulation, the tip should be 0.7 mm^2*
- Acrylic chambers with wire grid floors
- 70% ethanol to sanitize handheld force transducer and its polypropylene tip
  Note: *Handheld force transducer should be gently cleaned given its sensitivity*
- A tilted mirror below the wire grid floor may be used to provide a clear view of the animal's hind paw
- von Frey test template

PROTOCOL

A. Set up, habituation, and overall procedure (Days -6, -5, -4, and -3)

1. See SOP1: General setup, habituation, and post-test procedures.
2. Mice are habituated in 120-minute sessions during 4 consecutive days prior to the behavioural testing.
3. Paper towel may be removed if investigator uses the tilted mirror for measurements.
4. Improper habituation, animal handling, or excessive application of the filaments (either number of times or interval between applications) may result in exaggerate response resulting in false positive readings.

B. Baseline (Day -2 and -1) and post-endometriosis induction measurements

Note: The test consists of evoking a withdrawal response via a handheld force transducer adapted with a polypropylene tip. The investigator needs to apply the tip perpendicularly to the bladder region with a gradual increase in pressure.

1. See SOP2: Manual version of von Frey filaments (abdomen and hind paw) (C. Abdominal von Frey measurements) for acclimatization and response requirements prior to behavioural testing.
2. Using the 0.7 mm^2 tip, the investigator needs to apply an increased perpendicular force to the bladder region of the mouse.
   Note: *The upper limit pressure must be 15 g to ensure that the response will be related to withdrawal to a noxious stimulus.*
3. A positive response is characterized by one of the following behaviours: sharp retraction of the abdomen, immediate licking or scratching of the tip application spot, jump, and flinches.

4. After abdominal withdrawal, the intensity of the pressure is automatically recorded in the electronic aesthesiometer. The final recorded response value is obtained by averaging three measurements.
5. Ensure all equipment is clean (i.e., wiped down with disinfectant and/or 70% ethanol) before testing another round of animals.

C. Post-testing

1. See SOP1: General setup, habituation, and post-test procedures.

D. Data plotting

1. The results are expressed by delta (Δ) withdrawal threshold (in grams), which is calculated by subtracting the mean measurements post-stimulus from the baseline measurements.

REFERENCE

Colombo BB, Fattori V, Guazelli CFS, Zaninelli TH, Carvalho TT, Ferraz CR, Bussmann AJC, Ruiz-Miyazawa KW, Baracat MM, Casagrande R et al. Vinpocetine Ameliorates Acetic Acid-Induced Colitis by Inhibiting NF-κB Activation in Mice. *Inflammation*. *2018*; 41(4): 1276-1289.

SOP 3b: Electronic von Frey test (hind paw; non-bending filament)

Purpose
Measure precise pain intensity in the hind paws of rodents to a mechanical stimulus. It involves an automated electronic probe. The procedure has been used and described by Ge *et al*., 2019; Liu *et al*., 2018; Martinov *et al*., 2013; and Vivancos *et al*., 2004.

Relevance to humans
See SOP2.

Supplies and equipment
The following equipment list is specific for electronic von Frey in rats.

- Electronic von Frey (e.g., cat# 38450; Ugo Basile; Gemonio, VA, Italy)
- Touch Stimulator Transducer with glass prism (metal non-bending filament, e.g., cat# 38450-004; Ugo Basile; Gemonio, VA, Italy)
- Elevated base (e.g., cat# 37450-278) with perforated metal sheet (e.g., cat# 37450-005; Ugo Basile; Gemonio, VA, Italy).
- Modular enclosures (e.g., cat# 37000-006; Ugo Basile; Gemonio, VA, Italy)
- 70% ethanol to sanitize the chambers, perforated sheet, and base before and after testing session
- Laboratory notebook with von Frey template

PROTOCOL

A. Set up and habituation

1. Set base with perforated sheet and modular enclosures. Clean well with 70% ethanol and set paper towel or absorbent pads at the base to collect any excretion from animal.
2. Ensure all equipment is properly connected before testing.
3. Rats should be brought 45 minutes before testing. It takes about 30 minutes for rats to have low activity as evidenced by the animal resting or curling up to sleep (since testing is usually carried out during their wind down to sleeping). After animal activity is low, tester should enter the room quietly and stay there 15 minutes before starting the test.

B. Baseline and post endometriosis induction measurements.

1. Using the metal tip attached to the transducer, the tester needs to apply increased perpendicular force in a steady rate in the plantar surface of the hind paw.
   Note: *Never apply force to animal standing on only hind paws since the weight of animal will not be properly distributed and will affect greatly the results. The animal must be on all paws.*
2. Paw withdrawal or licking will be considered a positive response to the stimulus.
3. Take 3 measurements from each hind paw with a 15 second interval between each measurement.

C. Post testing

1. See SOP1: General setup, habituation, and post-test procedures.

D. Data Plotting

1. If using the von Frey template, tabulate data in excel and average measurements for each rat.
2. If data was transferred from eVF to computer using the Data Collection Application Software, export it to Microsoft Excel and organize data per animal for proper analyses.

REFERENCES

Ge P, Ren J, Harrington AM, Grundy L, Castro J, Brierley SM, Hannig G. Linaclotide treatment reduces endometriosis-associated vaginal hyperalgesia and mechanical allodynia through viscerovisceral crosstalk. *Pain*. *2019*; 160(11): 2566-2579.

Liu Z, Chen S, Qiu C, Sun Y, Li W, Jiang J, Zhang J-M. Fractalkine/CX3CR1 Contributes to Endometriosis-Induced Neuropathic Pain and Mechanical Hypersensitivity in Rats. *Front Cell Neurosci*. *2018*; 12: 495.

Martinov T, Mack M, Sykes A, Chatterjea D. Measuring changes in tactile sensitivity in the hind paw of mice using an electronic von Frey apparatus. *J Vis Exp*. *2013*; (82): e51212.

Vivancos GG, Verri WA Jr, Cunha TM, Schivo IR, Parada CA, Cunha FQ, Ferreira SH. An electronic pressure-meter nociception paw test for rats. *Braz J Med Biol Res*. *2004*; 37(3): 391-399.

SOP 4: Visceromotor response (VMR) to vaginal distension

Purpose

Measurement of the visceromotor response (VMR) to distention of a hollow pelvic organ is commonly used to provide a quantifiable and objective measure of relative visceral sensitivity in rodents. Descriptions are provided for carrying out VMR during vaginal distension with chronically or acutely implanted electrode wires. This procedure was developed in the laboratory of Dr. Julie Christianson by adapting the methodology used to measure VMR during colorectal distention. Other groups have developed similar techniques (Castro *et al*., 2021; Maddern *et al*., 2022).

Relevance to humans

Pelvic organ distention, either via balloon (colon and vagina) or instillation of liquid (bladder), is used clinically to determine pain thresholds in patients with chronic pelvic pain syndromes (Simrén *et al*., 2018; Tu *et al*., 2017). Patients with provoked vestibulodynia report vaginal allodynia in response to non-noxious and noxious distention of the vagina (Farmer *et al*., 2013). While recording the VMR to known distention pressures is not equivalent to stated pain scores in humans, it does provide an objective measure of physiological response to a known stimulus and is comparable across groups. It is also the closest measure for visceral pain.

Supplies and equipment

The following equipment list is specific for vaginal balloon distension (VBD) in mice. Only equipment directly used for carrying out VBD and measuring VMR are included. Additional items considered to be standard laboratory equipment and PPE are not listed.

- PE-160 tubing
- 18G blunt needles
- Luer valve assortment kit
- Non-lubricated latex condoms
- Silk thread
- Water-based lubricant
- Broome style restraint
- Compressed nitrogen tank
- Pressure monitor and valve interface or rodent barostat
- Small animal non-rebreathing anaesthesia system
- Compressed oxygen tank with regulator
- Teflon-coated electrode wire (0.003” diameter)
- 25G x 1-inch sterile hypodermic needles
- Tygon silicone tubing
- Micrograbber test clips
- Alligator clips
- Miniature siliflex lead wire
- BNC cables
- Manifold
- Differential AC Amplifier
- Analog/digital interface
- Computer

- EMG analysis software (e.g., Spike2)

Additional equipment for chronic electrode implantation

- Hollow plastic tube (e.g., coffee stirrer or thin drinking straw)
- Hair clippers
- 7-0 Prolene sutures
- 4-0 Silk sutures

PROTOCOL

A. Balloon construction

1. Cut a ~10-12cm piece of PE-160 tubing and attach one end to a blunt-end 18G needle.
2. Insert the other end into the tip of a non-lubricated latex condom and unroll the condom down approximately 3cm.
3. Pull the end of the tubing ~1-2mm away from the tip of the condom to allow for air flow. Tightly wrap silk thread around the condom, 1cm from the tip, and tie a tight knot.
   ***Note**: Balloons of 0.3cm length have also been similarly constructed and used (Maddern et al., 2022). Examples of completed balloons are shown in Figure 5.*
4. Test the balloon with a 15ml syringe filled with room air.
5. Trim the thread and excess condom. Store balloon in water-based lubricant to prevent degradation.
   ***Note**: A schematic diagram for connecting the entire system can be found in Figure 6 and in (Christianson and Gebhart, 2007).*

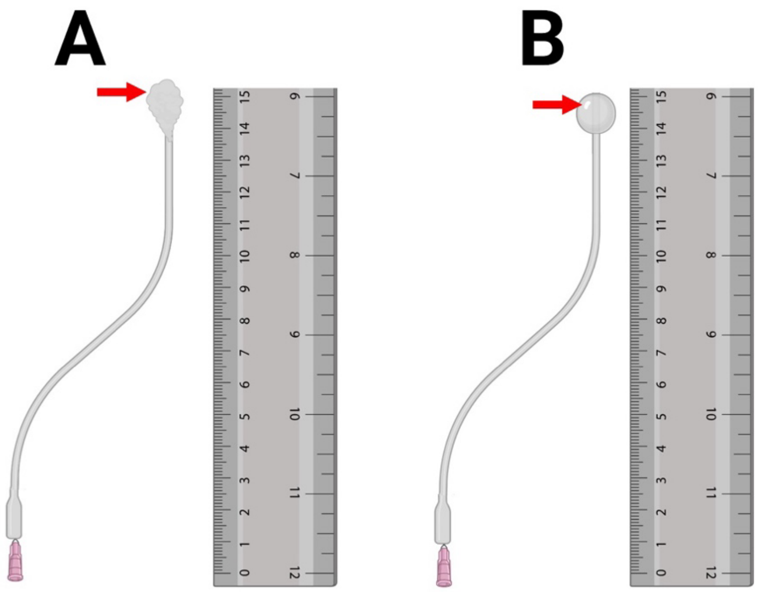

***Figure 5. Construction of intravaginal balloon for carrying out vaginal balloon distention.** A) The balloon is constructed of the end of a latex condom tie tightly onto PE-160 tubing. The red arrow points to the end of the tubing, which is 1-2mm from the end of the balloon to allow for air flow and inflation of the balloon. B) The balloon is filled with room air via a 15ml syringe. The red arrow points to the end of the tubing. Also schematized in (Christianson and Gebhart, 2007). Image generated using BioRender.com.*

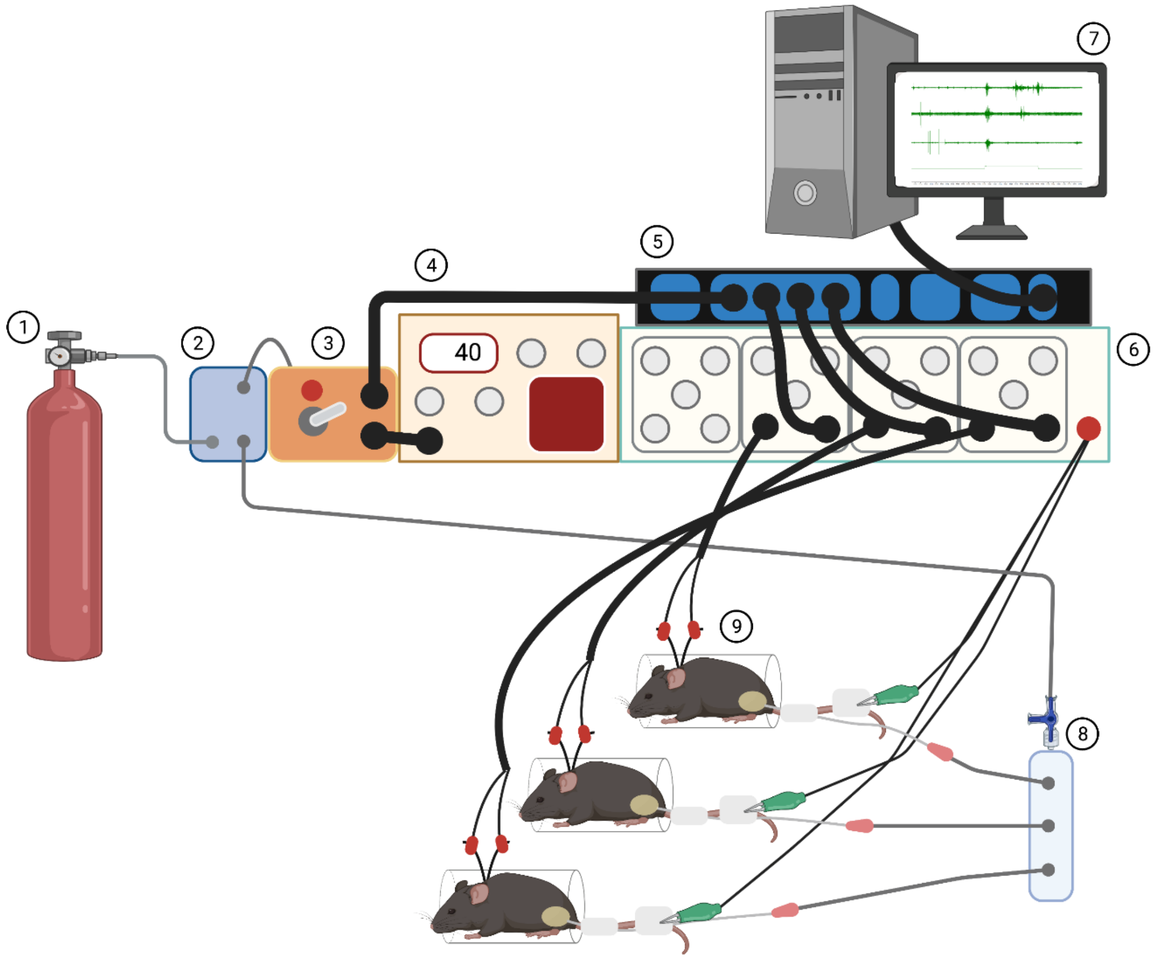

Figure 6. A schematic diagram of the VBD set up. *A compressed nitrogen tank (1) is connected to the valve box (2) that sends connections to the valve interface (3) and manifold (8) that releases air to the balloons. The valve interface (3) is connected to the pressure monitor (4) to allow for precise pressure measurements, and to the A-D converter (5). The A-D converter receives input from the differential amplifier (6) that is connected to the electrode wire implanted in the mice (9). The output from the A-D converter (5) ultimately is received by the workstation (7) that records the electromyographic activity from the electrode wires (9). Also schematized in (Christianson and Gebhart, 2007). Image generated using BioRender.com.*

B. Electrode implantation

<u>Chronic implantation</u>

1. Prepare a dedicated surgical area for aseptic surgery according to approved animal care and use guidelines.
2. Anesthetize one mouse with inhaled 4% isoflurane and reduce to 1-2% to maintain an appropriate depth of anaesthesia.
3. Use clipper to remove hair on the right lower abdomen and at the back of the neck.
4. Wipe away excess hair and disinfect the area with Betadine or other approved disinfectant.
5. Place the mouse in the supine position. Make a small (1cm) lateral incision in the skin and fascia of the right abdomen, just rostral to the inguinal ligament. Using small scissors or forceps, separate the muscle from the overlying skin around the incision.

6. Place the mouse in the prone position and make a small (1cm) incision in the skin overlying the back of the neck. Using small scissors or forceps, separate the muscle from the overlying skin, in the direction towards the abdominal incision.
7. Insert the sterilized plastic tube into the incision at the neck and pass it subcutaneously until it emerges through the abdominal incision. Remove any fascia that may be blocking the emergent end of the tube.
8. Thread two 12-15cm-long pieces of sterilized electrode wire through the tube, from the neck towards the abdomen. Hold on to the abdominal ends of the electrode wires while removing the plastic tube through the incision in the neck. Use a piece of tape or other approved means to stabilize the electrode wires at the neck incision to prevent them from being pulled through while implanting them.
9. Place the mouse in the supine position. Strip approximately 0.5cm Teflon coating from the abdominal ends of both electrode wires. Bend the tip of one wire (~0.2cm) to approximate a fishhook and insert the bent end into the tip of a 25G needle. Hold the wire taut against the needle and insert it into the abdominal muscle, in the cranial direction, so that it runs parallel with the muscle fibres and the depth exceeds the length of the bent wire (~0.3cm past the bevel of the needle). Hold the wire stable while removing the needle. The implanted wire should now be embedded in the muscle tissue.
10. Repeat the previous step with the remaining electrode wire, placing it approximately 0.5cm away from the first wire to avoid contact.
11. Place a single suture (recommended 7-0 Prolene or 5-0 silk) in the muscle, around each electrode wire, below the insertion point. Pull the free ends of the wires, at the back of the neck, gently to remove any excess slack, while allowing the wires to form a bend towards the tail. Insert another suture into the muscle, around both wires at the point where they bend back towards the neck, to anchor them in place.
12. Suture the abdominal incision closed.
13. Place the mouse in the prone position. Secure each electrode wire so that they exit on opposite ends of the incision at the neck. Suture the incision closed. Adjust the mouse so that the electrode wires are fully extended internally and trim the exteriorized leads to ~2-3cm in length.
14. Treat the mouse with approved postoperative analgesia.
15. House mice separately to avoid damage to the exteriorized electrode wire. Allow for at least 4 days of recovery prior to performing VBD.

Acute implantation

1. Anesthetize one mouse with inhaled 4% isoflurane and reduce to 1-2% to maintain an appropriate depth of anaesthesia.
2. Obtain two electrode wires, approximately 15cm in length and strip ~0.5cm of Teflon coating from a single end of both wires. Bend the tip of an exposed wire (~0.2cm) to approximate a fishhook and insert the bent end into the tip of a 25G needle.
3. With the mouse in the supine position, lift the right abdominal musculature and overlying skin and insert the needle into the muscle in the cranial direction, past the depth of the bend in the wire. Hold the wire and muscle in place and remove the needle. Gently tug on the wire to ensure that it is implanted into the muscle. If it appears to be too superficial or evacuates the mouse, repeat the procedure.
4. Implant the second wire, using the same procedure, into the left abdominal musculature. This ensures that the ends will not touch.

C. VBD protocol

1. Turn on all electrical equipment and the software program being used to capture EMG activity.
2. If electrodes have been chronically implanted, expose one mouse to 4% isoflurane and once fully anesthetised reduce to 2% isoflurane.
   Note: *Mice with acutely implanted electrodes will already be anesthetised.*

3. Remove the balloon from the water-based lubricant and use a tissue to remove all lubricant from the PE tubing.
4. Place a 15ml syringe into the needle and fully collapse the balloon for easier insertion.
5. Insert the balloon into the vagina until the tied end is no longer visible. Use blunt-end forceps to gently insert any exposed parts of the balloon, if necessary. Secure the tubing to the tail using a piece of masking tape. An additional piece of surgical tape can be used to further secure the balloon.
6. Gently place the mouse into a Broome-style restraint, ensuring that the electrodes are easily accessible (through the top for chronically implanted and out by the tail for acutely implanted), all limbs are secured inside, and that the mouse has room to easily breathe, but not twist or escape.
7. Strip the Teflon coating from a portion of the exteriorized electrode wires and connect to the differential amplifier using micrograbber clips. Ground the mouse by attaching an alligator clip to a gauze pad soaked in 0.9% saline wrapped around the tail.
8. Repeat balloon implantation, restraint positioning, and electrode attachment for up to 3 additional mice, if desired.
9. Connect the balloons to the outlet manifold and ensure that the output to the balloons is closed.
10. Allow mice to recover from anaesthesia for at least 30 minutes prior to testing.
11. While mice recover, ensure that the EMG recordings are stable with relatively low noise. Set the balloon pressure to the initial pressure using the regulator on the nitrogen tank and the read-out from the pressure monitor.
12. Begin recording the EMG activity and open the air flow to the balloons.
13. For each pressure being tested, distend the balloon for 20-30 seconds. Allow the mice to recover for 4 minutes before the next distention.

    Note: *Different pressure series and applications have been used to measure VMR during VBD. In general, naïve mice do not respond to pressures below 40mmHg (Maddern et al., 2022; Pierce et al., 2014; Pierce et al., 2015). Generally, pressure series will increase by 10-20mmHg, with maximum pressures reaching 80-120mmHg. Pressures can be applied once or in triplicate.*
14. Once finished, remove the balloon from the manifold, detach the micrograbbers and ground clip, and place the mouse in the restraint into a chamber filled with 4% isoflurane until anesthetized. Remove the balloon (and gently tug out the electrodes if implanted acutely) and return the mouse to its home cage.
15. The VBD procedure can be repeated on the same mouse for longitudinal or pre-/post-treatment experiments.

REFERENCES

Castro J, Maddern J, Grundy L, Manavis J, Harrington AM, Schober G, Brierley SM. A mouse model of endometriosis that displays vaginal, colon, cutaneous, and bladder sensory comorbidities. *FASEB J*. *2021*; 35(4): e21430.

Christianson JA, Gebhart GF. Assessment of colon sensitivity by luminal distension in mice. *Nat Protoc*. *2007*; 2(10): 2624-2631.

Farmer MA, Maykut CA, Huberman JS, Huang L, Khalifé S, Binik YM, Apkarian VA, Schweinhardt P. Psychophysical properties of female genital sensation. *Pain*. *2013*; 154(11): 2277-2286.

Maddern J, Grundy L, Harrington A, Schober G, Castro J, Brierley SM. A syngeneic inoculation mouse model of endometriosis that develops multiple comorbid visceral and cutaneous pain like behaviours. *Pain*. *2022*; 163(8): 1622-1635.

Pierce AN, Ryals JM, Wang R, Christianson JA. Vaginal hypersensitivity and hypothalamic-pituitary-adrenal axis dysfunction as a result of neonatal maternal separation in female mice. *Neuroscience*. *2014*; 263: 216-230.

Pierce AN, Zhang Z, Fuentes IM, Wang R, Ryals JM, Christianson JA. Neonatal vaginal irritation results in long-term visceral and somatic hypersensitivity and increased hypothalamic-pituitary-adrenal axis output in female mice. *Pain*. *2015*; 156(10): 2021-2031.

Simrén M, Törnblom H, Palsson OS, van Tilburg MAL, Van Oudenhove L, Tack J, Whitehead WE. Visceral hypersensitivity is associated with GI symptom severity in functional GI disorders: consistent findings from five different patient cohorts. *Gut*. *2018*; 67(2): 255-262.

Tu FF, Kane JN, Hellman KM. Non-invasive experimental bladder pain assessment in painful bladder syndrome. *BJOG*. *2017*; 124(2): 283-291.

SOP 5: Escape response to vaginal distension

Purpose

Animals are trained to perform an escape response to terminate vaginal distention produced by an inflatable latex balloon. Once animals are trained, testing sessions or "runs" consist of eight different distention volumes delivered three times each in random order at ~60 second intervals. The overall outcome measure is the animal's percent escape response to vaginal distention. These behavioural training and testing procedures for the assessment of vaginal nociception in the rat were developed by Dr. Karen Berkley and Dr. Stacy McAllister (Berkley *et al*., 2007; McAllister et al., 2012; McAllister *et al*., 2016; McAllister *et al*., 2009).

Relevance to humans

Escape response to vaginal distention has been successfully used to advance the understanding of mechanisms underlying pain, particularly peripheral and central contributors to endometriosis-associated pain (reviewed in Stratton and Berkley, 2011). Like patients, rats with endometriosis exhibit abnormal sensory signalling associated with the pelvic region, such as vaginal hyperalgesia (dyspareunia in women; McAllister *et al*., 2009). As in patients with endometriosis, hyperalgesic severity does not correlate with disease burden and there are individual differences in hyperalgesic severity between rats (McAllister *et al.*, 2016). The major limitations of vaginal escape response to assess for changes in vaginal nociception is that it requires specialized skills, is labour intensive, and is time consuming.

Materials

The following equipment list is specific for vaginal escape testing in <u>rats</u>. Only equipment directly used for carrying out this procedure are included. Additional items considered to be standard laboratory equipment and PPE are not listed.

<u>Behavioural apparatus</u>

The training and testing chamber is a small rectangular, grill-floored Plexiglass® chamber designed to contain the rat just enough to prevent her from turning around (see Figure 7A). A hollow tube containing light-emitting diodes and a photosensor extend from the front of the testing chamber (see Figure 7B). If the rat extends her nose into this tube and performs an "escape response" the light beam breaks and the stimulus is terminated. An opening in the rear of the chamber allows the catheter (attached to the vaginal stimulator) to be connected to the computer-controlled and automated stimulus-delivery device.

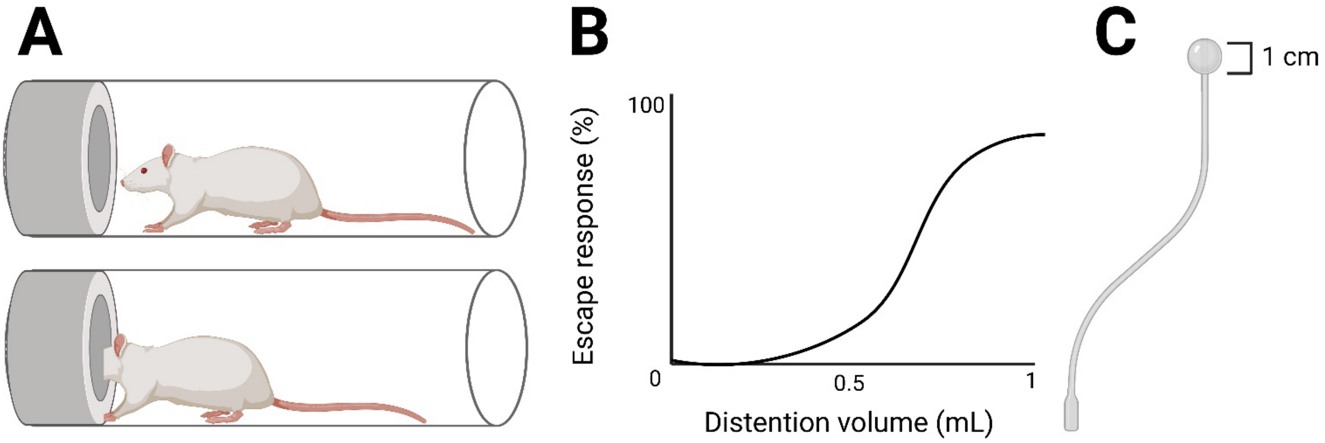

Figure 7. Assessment of vaginal nociception. *(A, top) Rat resting in testing chamber. (A, bottom) Rat escaping from vaginal distention (nose in tube). (B) Percentage of escape response graphed as a function of vaginal distention volume*

(mL) from a typical baseline testing session. (C) Schematic of the latex balloon vaginal distention device. Image generated using BioRender.com.

Supplies and equipment

Vaginal balloon

- 4-0 Silk suture
- Abbott butterfly winged infusion set 25G x 1.9cm (3/4”) 30 cm (12”) catheter tubing
- TROJAN brand non-lubricated latex condom
- KY jelly
- Saran plastic wrap
- 1cc Syringe
- Deionized water
- Plastic ruler
- Dissecting scissors
- Handling forceps

PROTOCOL

A. Balloon construction

1. Cut off the butterfly (needle) end of the tubing including the curved portion of the tubing.
2. Mark 6mm from the end of the tubing where it was recently cut.
3. Place the latex condom on the end of the tube that was recently cut and marked off.
4. Lightly fasten the condom onto the tubing using silk suture (4.0) and a clove hitch at the 6mm mark. This will leave 4 mm of the condom “hanging” off the end of the tubing.
5. Pull suture ends on the clove hitch to tightly fasten the condom securely around the tubing but without crimping the tubing.
6. Attach a 1 cc syringe filled with deionized water to the luer connector end of the tubing. Slowly push the syringe plunger to fill the condom with deionized water and check for leaking and crimping of the tubing.
7. Use the syringe to remove any air bubbles in the condom (balloon) and tubing.
8. Attach the balloon to the testing apparatus via the luer connector. Use the Pelstim program to run an “In Air Pressure” test and confirm the balloon is suitable (end pressures between 70 and 80 mmHg).
9. *If the balloon is suitable:* carefully cut off the excess latex condom below the suture using dissecting scissors, label the balloon with the appropriate animal number, and wrap the balloon in saran wrap with KY jelly.
10. *If the balloon is not suitable:* adjust the balloon using handling forceps. If end pressures are too high, loosen the clove hitch and make the balloon larger in size and then re-test. If end pressures are too low, loosen the clove hitch and make the balloon smaller in size and then re-test.

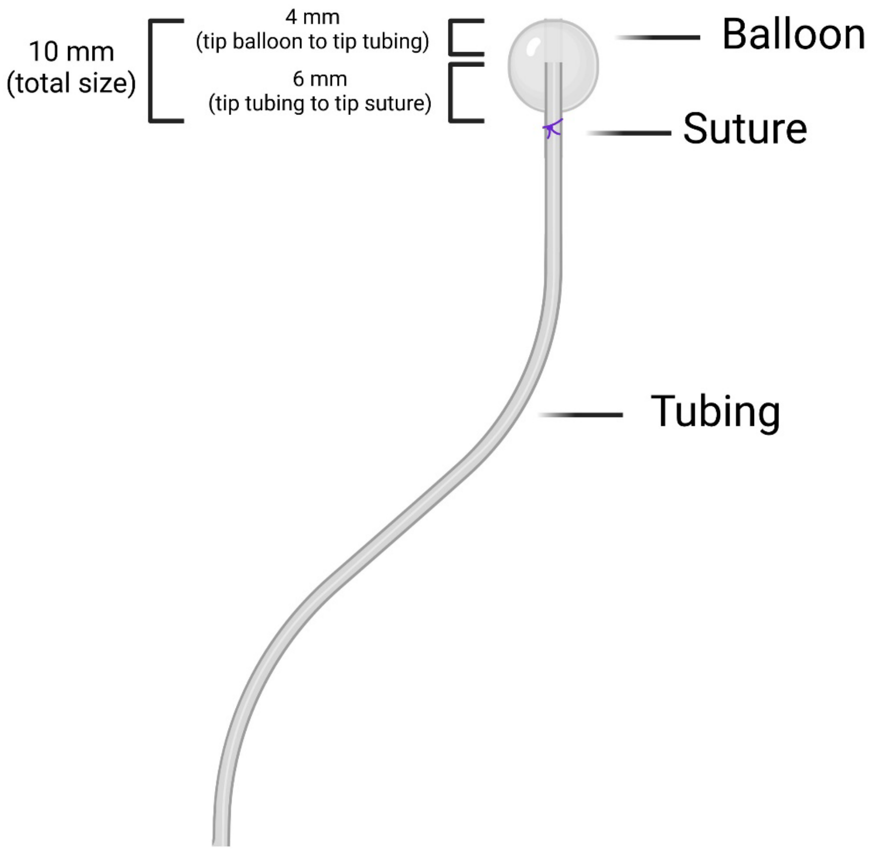

***Figure 8. Construction of vaginal balloon for carrying out vaginal balloon distention**. The balloon is constructed of the end of a latex condom tied tightly onto catheter tubing with silk suture. The end of the tubing is ~4mm from the end of the balloon to allow for water flow and balloon inflation. Water delivery to the balloon is computer controlled. Image generated using BioRender.com.*

B. Behavioural training

Training consists of 3 stages as follows: box training, tail pinch training, and vaginal training.

<u>Box training</u>

The goal of box training is to acclimatize the animal to the testing chamber and have her facing forward without trying to turn around. This stage of training can be done on consecutive days and is usually completed in ~3-4 sessions of 10 minutes each.

1. Clean the testing chamber.
2. Record the animal number, smear stage, and user information.
3. Retrieve the rat.
4. Record the start time.
5. Place the rat in the testing chamber facing forward.
6. If the animal tries to turn, lightly pull her tail until she turns back to the forward position.
   ***Note:** The tail should be pulled near the base of the tail and NEVER near the tip.*
7. Record detailed notes about the animal's behaviour.
8. Remove the rat from the testing chamber after ~10 minutes.
9. Record the training completion time.

10. Give the rat a tiny dab of peanut butter on a wooden stick.
11. Return the rat to colony.
12. Clean the testing chamber.

Tail pinch training

The goal of tail pinch training is to shape a required "escape response" which is the rat extending or "poking" her head into a tube to interrupt an infrared light beam. Training sessions consist of 10 tail pinches delivered at ~1min intervals. This stage of training is done on non-consecutive days and is usually completed (>80% escape behaviour) in ~4-8 sessions.

13. Repeat the first 5 steps described in "*Box training"*, above.
14. Lightly pinch the rat's tail with padded forceps until she "breaks the beam" or a maximum of 10 seconds has elapsed.
    Note: *Pinch the tail in a different spot each time and never on the TIP!*
15. Repeat "pinch" at ~1 min intervals.
    Note: *Increase the number of pinches with the number of training sessions. For example, Day 1: 3 to 5 pinches, Day 2: 5 to 7 pinches, Day 3: 7 to 10 pinches.*
16. Record detailed notes about the animal's behaviour after each tail pinch.
17. After a *maximum of 10* tail pinches, remove the rat from the testing chamber.
18. Repeat the last 4 steps described in "*Box training"*, above.

Vaginal training

The goal of vaginal training is for the rat to make an identical "escape response" (as above) to terminate vaginal distention stimuli (i.e., the balloon). Training sessions consist of 10 large distention volumes (0.80 to 1.0 mL, inflation rate 1 mL/s) delivered at ~1min intervals for a maximum of 15 sec. All rats show some behavioural response to these stimuli, which allows for balloon deflation to shape the rat's escape response. This stage of training is done on non-consecutive days and is usually completed in ~3-5 sessions. Training is complete when the rat successfully performs an "escape response" to terminate all 10 delivered stimuli. An individual vaginal training file is set up for each rat using the Pelstim computer program with a maximum distention volume initially set at 0.80 ml. The computer delivers the volumes per the parameters above.

19. Clean the testing chamber.
20. Open the Pelstim program.
21. Select and load the appropriate rat identification number vaginal training file.
22. Enter date, smear stage, and user information.
23. Attach the individual rat's balloon to the testing apparatus.
24. Go to the Utilities menu and select "Zero Balloon". This calibrates the pressure inside the balloon to zero.
25. Go to Utilities menu and run "Before in Air Pressure". This tests the pressures of the balloon at increasing volumes.
26. Retrieve the rat.
27. Place the rat in the testing chamber.
28. Insert the rat's deflated balloon into her mid-vaginal canal (a little KY jelly may be used).
29. Tail pinch the rat 3 times and record observations.
30. Go to the Trials menu and select RUN.
31. When the trials begin the balloon will inflate and the rat will:
    - Perform the required "escape response" to terminate the stimulus (deflate the balloon). In this case, proceed with the vaginal training trials with no additional interventions.

  - *Not perform* the required "escape response." In this case, pinch the rat's tail to encourage the previously learned "escape response." As trials progress and the rat starts performing the "escape response" to terminate vaginal distention, stop the tail pinch. Adjust training as needed for each individual rat.

32. Enter detailed notes on the animal's behaviour after each trial.
33. Confirm the balloon is in position after every trial (via mirror underneath testing chamber).
34. A menu prompt will appear after all trials are complete, select "ok".
35. Remove the vaginal balloon from rat.
36. Remove the rat from the testing chamber.
37. Give the rat a tiny dab of peanut butter on a wooden stick.
38. Go to the Utilities menu and run "After in Air Pressure."
39. Save the vaginal training session.
40. Return the rat to the colony.
41. Store the balloon (in KY jelly/saran wrap and labelled w/animal number).
42. Clean the testing chamber.

At the end of the training session, adjust the rat's max volume if needed. For example, if the rat does not perform an "escape response" to all 10 trials, increase the max volume (e.g., from 0.80 ml to 0.90 mL) in preparation for the next training session.

C. Behavioural testing

Once animals are trained, testing sessions begin to assess vaginal nociception. Testing sessions consist of a series of 24 computer-controlled escape trials run at ~1min intervals (range 50 – 70 s). Each trial consists of a rapid inflation of the balloon (1 mL/s) to a fixed volume until the rat makes an "escape response" or 15 sec has elapses, when the balloon rapidly deflates (0.5 mL/s). Eight different distention volumes, including a control volume (0.01 mL) and the max volume determined in vaginal training, are delivered 3 times each in random order. The maximum latency of 15 s is considered to be no response. Testing is done on non-consecutive days 3-4 times/week. A minimum of 3 runs/oestrous stage are collected per testing period (e.g., baseline or post endometriosis induction). An individual testing file is set up for each rat using the Pelstim computer program. The computer delivers the volumes per the parameters above. The experimenter is blinded to the volumes delivered to the rat. Results from a typical baseline testing session are shown in Figure 7D.

1. Repeat all steps in *Vaginal training*, above.
   Note: The Pelstim program file will be saved as a test rather than trial file.
   Note: No intervention should be needed during the distension protocol.
2. Clean the testing chamber.

REFERENCES

Berkley KJ, McAllister SL, Accius BE, Winnard KP. Endometriosis-induced vaginal hyperalgesia in the rat: effect of estropause, ovariectomy, and estradiol replacement. *Pain*. *2007*; 132(Suppl 1): S150-159.

McAllister SL, Dmitrieva N, Berkley KJ. Sprouted innervation into uterine transplants contributes to the development of hyperalgesia in a rat model of endometriosis. *PLoS One*. *2012*; 7(2): e31758.

McAllister SL, Giourgas BK, Faircloth EK, Leishman E, Bradshaw HB, Gross ER. Prostaglandin levels, vaginal innervation, and cyst innervation as peripheral contributors to endometriosis-associated vaginal hyperalgesia in rodents. *Mol Cell Endocrinol*. *2016*; 437: 120-129.

McAllister SL, McGinty KA, Resuehr D, Berkley KJ. Endometriosis-induced vaginal hyperalgesia in the rat: role of the ectopic growths and their innervation. *Pain*. *2009*; 147(1-3): 255-264.

Stratton P, Berkley KJ. Chronic pelvic pain and endometriosis: translational evidence of the relationship and implications. Hum Reprod Update. 2011 May-Jun;17(3):327-46.

SOP 6: Hargreaves test (hind paw)

Purpose

Pain intensity to a radiant heat thermal stimulus (heat hyperalgesia) directed to the hind paw regions can be measured using the Hargreaves test, sometimes called the plantar test or radiant heat test. A radiant heat source is used to stimulate the paw by gradually increasing the temperature of the plantar surface and a withdrawal response to the stimulus (e.g., flinching or licking) is considered as a “positive” response. The concept for establishing an appropriate baseline for the Hargreaves test is that control (or pre-endometriotic) animals should have a response latency to the radiant heat stimulus of around 10-15 seconds, the reason being that this is not too long to expect a mouse to remain still but will allow for a good response window to be established between the controls and the mice showing thermal hyperalgesia, the latter of which would be expected to be have a response latency of around 4-7 seconds. This procedure is described by Fattori *et al*., 2020 and Yan *et al*., 2019.

Relevance to humans

An evoked response to heat stimulus is frequently used as a surrogate of heat thermal sensitivity in rodent models of endometriosis. This is a relatively easy test to perform and produce reproducible data. However, sensitivity to heat stimuli is rarely reported by individuals with endometriosis (Coxon *et al*., 2021). In addition, a lack of heat sensitivity in a non-surgical model (Fattori *et al*., 2020) might indicate that surgery could be a factor influencing heat thermal hyperalgesia in rodent models of endometriosis. Therefore, these might be limiting factors that may reduce clinical relevance for this test.

Supplies and equipment

The following equipment list is specific for Hargreaves testing in <u>mice</u>. Only equipment directly used for carrying out the Hargreaves method are included. Additional items considered to be standard laboratory equipment and PPE are not listed.

- Hargreaves apparatus (e.g., cat# 390G; IITC Life Science Inc., USA; cat# 37570; Ugo Basile SRL, Italy)

PROTOCOL

A. Set up, habituation, and overall procedure (Days -6, -5, -4, and -3)

1. See SOP1: General setup, habituation, and post-test procedures.
2. If any, turn on heated transparent glass plate and set temperature to 29°C.
3. Place a paper towel or an absorbent pad under the infrared generator. This helps blocking the noise that moving the infrared generator might produce.
4. Set laser intensity to desired potency in the infrared generator.
5. Mice need to be habituated in at least 60-minute sessions prior to the behavioural testing. Mice will urinate during the habituation, especially in the first 1 or 2 days and it is recommended to wipe up the urine during the habituation period as the mice cannot avoid the urine on the glass and it is stressful to them as they are clean animals when given the chance. Over the days of habituation, the urination will decrease.
6. Improper habituation, animal handling, or excessive application of stimulus (either number of times or interval between filaments) may result in exaggerate response to heat stimulus resulting in false positive readings.
7. Urine must be cleaned before testing as wet plantar surface might result less sensitivity to heat, resulting in false negative readings.

8. The distance between the glass and the halogen light source is critical as changing this distance will change the time at which each temperature is reached. Alternatively, use a sensitive digital thermometer to check what temperature is reached at 5, 10, 15, 20 and 25 seconds, and ensure this is the same for each area across the glass (Figure 9).

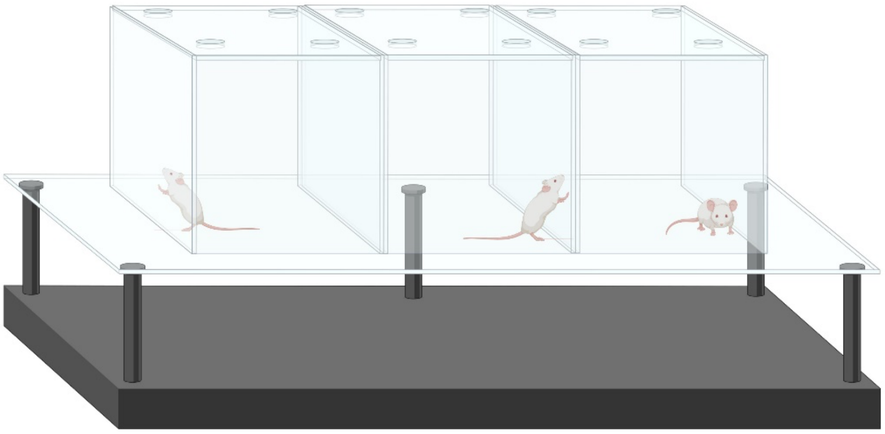

Figure 9. Example of Hargreaves test setup*. Observation area with individual mice in acrylic cages placed on top of a temperature-controlled glass plate. A radiant heat source is placed under the glass plate to stimulate the plantar surface of the hind paw. Image generated using BioRender.com.*

B. Baseline (Days -2 and -1), post-endometriosis induction measurements, and data plotting

A radiant heat source is used to stimulate the paw by gradually increasing the temperature of the plantar surface. A two-minute interval must be taken between measurements. See Figure 3 for the appropriate stimulation point in a mouse paw.

1. Follow the habituation and stimulation requirements described in SOP 2 (Abdominal von Frey measurements), above. If there is excessive urine on the glass this should be wiped up before testing begins and the mice left to settle down once more.
2. The investigator should carefully move the infrared generator and place it right below the mouse paw.
   Note: *Depending on the Hargreaves infrared generator used, paw withdrawal is detected a soon as the mouse withdraws its paw from the stimulus (i.e., the time is automatically recorded).*
3. A cut-off of 15 (high potency set lasers) to 60 (low potency set lasers) seconds is usually recommended to avoid tissue damage.
4. If no response is observed after the cut-off time is reached, cut-off time is recorded.
5. A positive response consists of either clear paw flinching or licking the stimulated area.
6. The threshold of pain is determined as the mean average of 3 latency (in seconds) to evoke a response. Record this number in a Hargreaves test template.

C. Post-testing

1. See SOP1: General setup, habituation, and post-test procedures

REFERENCES

Coxon L, Wiech K, Vincent K. Is There a Neuropathic-Like Component to Endometriosis-Associated Pain? Results From a Large Cohort Questionnaire Study. *Front Pain Res (Lausanne)*. *2021*; 2: 743812.

Fattori V, Franklin NS, Gonzalez-Cano R, Peterse D, Ghalali A, Madrian E, Verri WA Jr, Andrews N, Woolf CJ, Rogers MS. Nonsurgical mouse model of endometriosis-associated pain that responds to clinically active drugs. *Pain*. *2020*; 161(6): 1321-1331.

Yan D, Liu X, Guo S-W. The establishment of a mouse model of deep endometriosis. *Hum Reprod*. *2019*; 34(2): 235-247.

SOP 7: Hot plate test (hind paw)

Purpose

This test consists of introducing a rodent into an open-ended square space with a metallic plate floor at desired temperature, to assess thermal (heat) hyperalgesia in rodents. The endpoint is characterized by the removal of the paw followed by clear hind paw flinching or licking movements. It differs from the Hargreaves test in that all paws are in contact with the heat stimulus. Typically, the forepaws respond first to the heat and the hind paws second and it is typically the first hind paw response that is recorded, since it is sometimes difficult to differentiate between nocifensive and normal behavioural actions of the forepaws. The hot plate test was first used to evaluate endometriosis-associated pain by Lu *et al*. (2010) and has been routinely used in rodent models of endometriosis due to its ease of set-up and evaluation.

Relevance to humans

Like the Hargreaves test, the hot plate test is frequently used in rodent models of endometriosis to assess heat sensitivity and is relatively easy to perform. Nonetheless, sensitivity to heat stimuli is rarely reported by individuals with endometriosis (Coxon *et al*., 2021). Another major limitation of the hot plate is the learning component upon repeat testing. This generates a reduced latency time to produce one of the pain behaviours. While this can be easily overcome by performing single measurements, a lack of sensitivity is another limitation for the hot plate test. Therefore, these might be limiting factors that may reduce clinical relevance for this test.

Supplies and equipment

The following equipment list is specific for hot plate testing in <u>mice</u>. Only equipment directly used for carrying out the hot plate method are included. Additional items considered to be standard laboratory equipment and PPE are not listed.

- Hot plate apparatus (e.g., cat# BIO-CHP; Bioseb; cat# 76-0113; Harvard Instruments)
- Mirrors to be placed around the hot plate for visualizing withdrawal responses
- Chronometer for timing the duration spent on the hot plate

PROTOCOL

A. Set up and overall procedure (Day -3)

1. See SOP 1: General setup, habituation, and post-test procedures.
2. Due to the learning component of this test (Le Bars *et al*., 2001), the hot plate test should be performed up to once a day or preferably on alternate days for chronic models.
3. Improper animal handling or repeated testing may result in faster escape response due to stress or learning behaviour, respectively. Use of higher than 55 °C may result in tissue injury (Figure 10).
4. Habituation to room recommended for 30 minutes prior to testing is recommended but not essential.

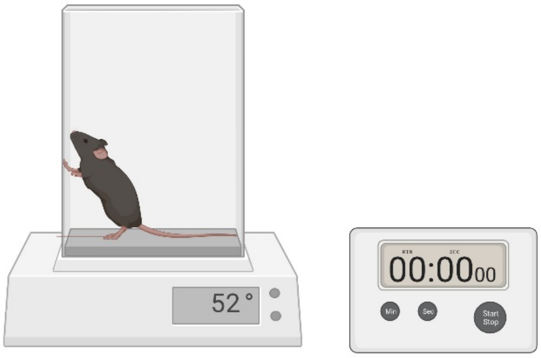

Figure 10. Example of hot plate setting up. *Hot plate apparatus set at 52 °C with an individual mouse in placed on it. A chronometer is used to determine the latency reaction as soon as the mouse touches the metallic plate. Image generated using BioRender.com.*

B. Baseline (Days -2 and -1), post-endometriosis induction measurements, and data plotting

1. Set hot plate to 52 °C ± 1ºC (temperature may vary from 50 ºC to 55 ºC ± 1ºC. It is not recommended exceeding 55 ºC for this test).
2. Wait at least 15 min after set temperature is reached to ensure that it is stable.
3. Gently place mouse on the hot plate. As soon as the mouse touches the metallic plate, the investigator should start the chronometer.
4. Tests are conducted with one mouse at the time.
5. Positive responses are characterized by the removal of the paw followed by clear hind paw flinching or licking movements or jumping.
6. At the baseline measurement, mice are expected to produce any of the above behaviours between 12 to 20 seconds.
7. If a mouse does not produce any of the behaviours within 20 to 30 seconds, this value is recorded, and the mouse is immediately removed from the hot plate.
8. The reaction time is registered in a hot plate template when any of the above responses are observed.
9. A cut-off of 12 to 20 seconds for all temperatures from 50 °C and higher should be set to avoid tissue damage.
10. The results are expressed by withdrawal latency (in seconds). Alternatively, results may be plotted as the delta (Δ) withdrawal threshold (in seconds), which is calculated by subtracting the mean measurements post-stimulus from the baseline measurements.
11. After cleaning with 70% ethanol in between animals, the investigator must wait until the plate reaches the set temperature again.

C. Post-testing

1. See SOP1: General setup, habituation, and post-test procedures.

REFERENCES

Coxon L, Wiech K, Vincent K. Is There a Neuropathic-Like Component to Endometriosis-Associated Pain? Results From a Large Cohort Questionnaire Study. *Front Pain Res (Lausanne)*. *2021*; 2: 743812.

Lu Y, Nie J, Liu X, Zheng Y, Guo S-W. Trichostatin A, a histone deacetylase inhibitor, reduces lesion growth and hyperalgesia in experimentally induced endometriosis in mice. *Hum Reprod*. *2010*; 25(4): 1014-1025.

SOP 8: Direct abdominal licking

Purpose

Rodents experiencing abdominal pain exhibit increased abdominal licking, which can be distinguished from other grooming behaviour by the absence of attention to other body areas. This behaviour can be quantified using clear observation chambers with an open floor to be placed on a wire floor grid, such that access to the rodent abdominal area and plantar surface is allowed or by using bottom-up video recording of these behaviours. Direct abdominal licking is defined as number of times, or the time spent attending the abdominal region without diverting any other region before or after the behaviours (i.e., if the mouse grooms any part of its body prior to or immediately after licking its abdomen it is not considered a spontaneous pain behaviour and rather is considered grooming). This procedure is described by Dorning *et al*., 2021, Fattori *et al*., 2020, and Greaves *et al*., 2017.

Relevance to humans

Grooming is a selfcare ethological behaviour produced by rodents. During abdominal pain, on the other hand, rodents may produce spontaneous pain behaviours, including direct abdominal licking. This behaviour is associated with the affective and motivational component of pain (Kimmey *et al*., 2022). C-low-threshold mechanoreceptive (C-LTMR) nociceptors perceive somatosensory inputs during self-grooming/licking and are not involved in direct pain response in non-pathological conditions (Seal *et al*., 2009; Vrontou *et al*., 2013). Therefore, similar to humans in which tactile-induced analgesia mildly contributes to pain relief (Mancini *et al*., 2015; Mancini *et al*., 2014), increases in direct abdominal licking behaviours might be related to an attempt of modelling pain relief via touch-induced analgesia (Lu *et al*., 2022). In fact, similar behaviour is observed in mice during self-generated whisking (self-generated sensory signals). This behaviour activates tactile nociceptors to significantly reduce facial sensitivity to heat and mechanical stimuli (Lu *et al*., 2022). Since spontaneous abdominal pain is the main complaint of patients with endometriosis, quantification of direct licking is likely to have higher clinical relevance for patients.

Materials

The following equipment list is specific for abdominal licking in <u>mice</u>. Only equipment directly used for carrying out this method are included. Additional items considered to be standard laboratory equipment and personal protection equipment (PPE) are not listed.

- Inferred Behaviour Observation Box (iBob; Crimson Scientific) or open floor observation arenas with individual chambers and wire grid floors
- If using the iBob, SD card and SD card reader

PROTOCOL

A. Set up, habituation, and overall procedure (Days -4, -3, -2, and -1)

1. See SOP1: General setup, habituation, and post-test.
2. *If using bottom-up video recording such as with iBob*, at least 4 rounds of 60-minute habituations are required (days -4 to -1).
3. *If using clear observation chambers with an open floor*, set up the platform such that it is elevated above the eye level of a seated technician. The platform should be placed in an area that allows the person to move around all sides of the platform without impeded access. Mice are placed in enclosure and allowed to acclimatize to the environment for a minimum of 30 minutes.

4. Improper habituation or animal handling may result in exaggerate grooming behaviour due to stress resulting in false positive scoring.

B. Post-endometriosis induction measurements and data plotting

1. No baseline is required.
2. If using bottom-up video recording such as with iBob, turn the iBob on and turn the bottom-up camera on. Then insert the SD card into the slot below the screen for the bottom-up camera.
3. Place each mouse into the previously cleaned chambers and put the top on the observation arena.
4. Close the iBob and start recording with this or another video recording device.
5. Return to the procedure room after 1 hour and stop the recording.
6. The recording for each group of mice will be saved on the iBob SD card as two 30-minute videos.
7. Return the mice to their home cages and then clean the iBob/clear chambers.
   Note: *Make sure there is enough space on the SD card for two 2.7GB videos prior to starting the next recording.*
8. Repeat for each group of mice.
9. After all the videos are collected and saved, they can be viewed for scoring.
10. Two 5-minute increments are scored from the videos for each group of mice recorded. The first segment scored starts on minute 2 of the first video and ends at minute 7. The second segment scored starts at the very beginning (0-min) of the second video and ends at minute 5.
11. Count each bout of abdominal licking for each mouse in each video segment and sum the licking bouts in both videos.
12. *If using clear observation chambers with an open floor*, periods of abdominally directed licking are recorded for 5 minutes, twice, during a 30-minute window. An average duration is generated as final scoring.

C. Post-testing

1. See SOP1: General setup, habituation, and post-test procedures.

REFERENCES

Dorning A, Dhami P, Panir K, Hogg C, Park E, Ferguson GD, Hargrove D, Karras J, Horne AW, Greaves E. Bioluminescent imaging in induced mouse models of endometriosis reveals differences in four model variations. *Dis Model Mech*. *2021*; 14(8): dmm049070.

Fattori V, Franklin NS, Gonzalez-Cano R, Peterse D, Ghalali A, Madrian E, Verri WA Jr, Andrews N, Woolf CJ, Rogers MS. Nonsurgical mouse model of endometriosis-associated pain that responds to clinically active drugs. *Pain*. *2020*; 161(6): 1321-1331.

Greaves E, Horne AW, Jerina H, Mikolajczak M, Hilferty L, Mitchell R, Fleetwood-Walker SM, Saunders PTK. EP2 receptor antagonism reduces peripheral and central hyperalgesia in a preclinical mouse model of endometriosis. *Sci Rep*. *2017*; 7: 44169.

Kimmey BA, McCall NM, Wooldridge LM, Satterthwaite TD, Corder G. Engaging endogenous opioid circuits in pain affective processes. *J Neurosci Res*. *2022*; 100(1): 66-98.

Lu J, Chen B, Levy M, Xu P, Han B-X, Takatoh J, Thompson PM, He Z, Prevosto V, Wang F. Somatosensory cortical signature of facial nociception and vibrotactile touch–induced analgesia. *Sci Adv*. *2022*; 8(46): eabn6530.

Mancini F, Nash T, Iannetti GD, Haggard P. Pain relief by touch: a quantitative approach. *Pain*. *2014*; 155(3): 635-642.

Mancini F, Beaumont AL, Hu L, Haggard P, Iannetti GDD. Touch inhibits subcortical and cortical nociceptive responses. *Pain*. *2015*; 156(10): 1936-1944.

Seal RP, Wang X, Guan Y, Raja SN, Woodbury CJ, Basbaum AI, Edwards RH. Injury-induced mechanical hypersensitivity requires C-low threshold mechanoreceptors. *Nature*. *2009*; 462(7273): 651-655.

Vrontou S, Wong AM, Rau KK, Koerber HR, Anderson DJ. Genetic identification of C fibres that detect massage-like stroking of hairy skin in vivo. *Nature*. *2013*; 493(7434): 669-673.

SOP 9: Abdominal contortions (writhing)

Purpose

Abdominal contortions are stereotypical spontaneous pain behaviours exhibited by rodents experiencing abdominal pain. It is classically characterized as a contraction of the abdominal muscle together with stretching of hind limbs. However, it can be also characterized as a contraction of the left oblique musculature with inward moving of the ipsilateral hindlimb. This procedure is described by Fattori *et al*., 2020.

Relevance to humans

To induce abdominal contortions, noxious agents are commonly injected intraperitoneally in rodents. Nonetheless, this behaviour is reported in a mouse model of endometriosis in the absence of a second stimulus, indicating that endometriosis induce these behaviours in mice. Importantly, abdominal contortions are not observed in healthy or pain-free rodents, indicating that is specifically seen during endometriosis or abdominal pain. Since spontaneous abdominal pain is the main symptom of endometriosis patients and because abdominal contortion test is one the commonly employed tests to screen drugs to abdominal pain (Collier *et al.,* 1968), its quantification (in the absence of a second stimulus) is likely to have higher clinical relevance for endometriosis patients.

Supplies and equipment

The following equipment list is specific for abdominal contortions in <u>mice</u>. Only equipment directly used for carrying out this method are included. Additional items considered to be standard laboratory equipment and PPE are not listed.

- Temperature controlled glass plate (e.g., as used for Hargreaves apparatus) OR heated glass plate
- Clear open floor observation arenas with individual chambers

PROTOCOL

A. Set up, habituation, and overall procedure (Days -6, -5, -4, and -3)

1. See SOP 1 – General setup, habituation, and post-test procedures.
2. Turn on temperature controlled/heated glass plate and set temperature to 29 °C.
3. Place each mouse in an individual chamber and leave the procedure room.
4. Allow the mice to acclimate to the chambers/procedure rooms for at least 60 minutes.
5. Improper habituation or animal handling may result in reduced abdominal contortions due to stress resulting in false negative scoring.

B. Post-endometriosis induction measurements and data plotting

1. No baseline is required.
2. Place each mouse in their individual chamber and allow them to acclimatize for at least 1 hour.
3. After the acclimatization time, return to the procedure room and sit silently for 5 minutes. If the mice are still displaying exploratory behaviours, allow them to acclimatize for another 30 minutes. If after those 30 minutes the mice are still displaying exploratory behaviours, allow them to acclimatize for yet another 30 minutes. If after two hours of acclimatization the mice are still displaying exploratory behaviours, then further habituation is required.
4. If the mice are no longer displaying exploratory behaviours, then observe them for 10 to 20 minutes counting the number of abdominal contortions that each mouse does.

13. A positive response for the abdominal contortions consists of stretching of the torso accompanied by a contraction of the abdominal muscle hyperextension of the hindlimbs and concave arching of the back. The absolute key motion that dictates the abdominal contortion are the hind legs being spread out wide or back with the front paws on the glass plate, in combination with pressing the abdomen against the glass plate. The concave arching of the back is not always obvious and sometimes is seen as the mouse lifting its head up while doing the previously stated motions. This behaviour may be also quantified using bottom-up video recording.

C. Post-testing

1. See SOP1: General setup, habituation, and post-test procedures.

REFERENCE

Collier HO, Dinneen LC, Johnson CA, Schneider C. The abdominal constriction response and its suppression by analgesic drugs in the mouse. *Br J Pharmacol Chemother*. *1968*; 32(2): 295-310.

SOP 10: Abdominal squashing

Purpose

Abdominal squashing is another stereotypical spontaneous pain behaviour exhibited by rodents experiencing abdominal pain. This is defined as the number of times the animal presses the lower abdominal region against the floor. The procedure is described by Fattori *et al*., 2020.

Relevance to humans

Similar to abdominal licking, increase in abdominal squashing might be related an attempt to produce touch-induced analgesia (Mancini *et al*., 2015; Mancini *et al*., 2014) in a mechanism that is similar to tactile-induced analgesia in humans (Lu *et al*., 2022) As mentioned previously, spontaneous abdominal pain is the main complaint of patients with endometriosis and differently from direct abdominal licking, abdominal squashing is not reported by healthy or pain-free mice. This indicates that this behaviour might be produced by rodents only during endometriosis or abdominal pain. Therefore, quantification of abdominal squashing is likely to have higher clinical relevance for endometriosis patients.

Supplies and equipment

The following equipment list is specific for abdominal squashing in mice. Only equipment directly used for carrying out this method are included. Additional items considered to be standard laboratory equipment and PPE are not listed.

- Clear open floor observation arenas with individual chambers

PROTOCOL

A. Set up, habituation, and overall procedure (Days -6, -5, -4, and -3)

1. See SOP 1: General setup, habituation, and post-test procedures.
2. Mice need to be habituated in 120-minute sessions prior to the behavioural testing.
3. Improper habituation or animal handling may result in reduced abdominal squashing due to stress resulting in false negative scoring.

B. Post-endometriosis induction measurements and data plotting

1. No baseline is required. Habituation and scoring may be performed in the same chambers as used for the von Frey test.
2. Allow the animals to acclimatize for 2 hours.
3. Return to the procedure room after acclimatization and sit silently for 5 minutes. Afterwards determine the number of mice that can be visualized easily at the same time and observe each group of mice for 10 minutes.
4. Count each time a mouse squashes its lower abdomen against the floor during 10 to 20 minutes.
5. This behaviour may be also quantified using bottom-up video recording. If measuring from the videos recorded from the iBob (see SOP 8*)* no further preparation is required. Count the number of times that each mouse squashes its lower abdomen against the floor in the same two 5-minute video segments that are used to quantify abdominal licking. As with the abdominal licking, sum the bouts of abdominal squashing from the two video segments.

C. Post-testing

1. See SOP1: General setup, habituation, and post-test procedures.

REFERENCES

Fattori V, Franklin NS, Gonzalez-Cano R, Peterse D, Ghalali A, Madrian E, Verri WA Jr, Andrews N, Woolf CJ, Rogers MS. Nonsurgical mouse model of endometriosis-associated pain that responds to clinically active drugs. *Pain*. *2020*; 161(6): 1321-1331.

Lu J, Chen B, Levy M, Xu P, Han B-X, Takatoh J, Thompson PM, He Z, Prevosto V, Wang F. Somatosensory cortical signature of facial nociception and vibrotactile touch–induced analgesia. *Sci Adv*. *2022*; 8(46): eabn6530.

Mancini F, Nash T, Iannetti GD, Haggard P. Pain relief by touch: a quantitative approach. *Pain*. *2014*; 155(3): 635-642.

Mancini F, Beaumont AL, Hu L, Haggard P, Iannetti GDD. Touch inhibits subcortical and cortical nociceptive responses. *Pain*. *2015*; 156(10): 1936-1944.

SOP 11: Thermal gradient

Purpose

By recording freely moving mice without the presence of an investigator, this assay measures the mouse own's determination of general discomfort. During endometriosis, mice are likely to display changes in thermal selection behaviour that indicate discomfort. A continuous temperature gradient is established along with a metallic base plate where the mice are gently placed. Mice then walk freely while being video recorded from above and after an exploration period individual mice show a distinct preference, indicating the most comfortable temperature range. This procedure is described by Fattori *et al*., 2020.

Relevance to humans

In general, healthy mice display higher preference for temperatures within the thermoneutral zone (30-34 °C) to produce resting/sleeping behaviours (Fischer et al., 2018). Disturbances in this set temperatures might indicate overall discomfort or difficulties at presenting resting/sleeping behaviours. Similarly, in a mouse model of endometriosis, sham mice (e.g., non-lesion bearing) prefer temperatures 27 to 36°C with a higher preference for 34°C, whereas mice with endometriosis exhibited a more dispersed pattern from 21 to 36°C with no single preferred temperature (Fattori *et al*., 2020). Therefore, this assay might correlate with presence of insomnia or sleep quality that patients with EAP experience (Facchin *et al*., 2021; Leone Roberti Maggiore *et al*., 2017; Youseflu *et al*., 2020). Nonetheless, in the current format, the investigators are not able to determine whether sleeping behaviours are being performed or impaired with the thermal gradient assay.

Supplies and equipment

The following equipment list is specific for thermal gradient in <u>mice</u>. Only equipment directly used for carrying out this method are included. Additional items considered to be standard laboratory equipment and PPE are not listed.

- Clear open floor observation arenas with individual chambers
- Thermal Gradient Test (e.g., Bioseb, which includes: 2 single plates BIO-CHP, aluminium alloy base plate, black PPC walls)

PROTOCOL

A. Set up and overall procedure (Day -1)

1. See SOP 1: General setup, habituation, and post-test procedures
2. Assemble the thermal gradient platform and create a new experiment in the software.
3. The room lighting is approximately 30-50 lux.
4. In the parameters for the experiment, set the two arenas and 20 zones to be measured.
5. Set the trial length to 90 minutes with a 30-minute habituation/exploration time during the trial.
6. Turn on the two single plates setting one hot and the other to cold. The goal is to have the base plate directly on top of the two plates to be 50 °C for the end on the hot plate and 4°C for the end on the cold plate. Increase/decrease the temperature of the plate until the base plate directly on top of the plates reaches the desired temperature (Figure 11).
7. Run a trial experiment to ensure the software is being able to correctly recognize each mouse in its own arena.
8. Improper habituation, animal handling, or room lighting may result in reduced animal activity/movement resulting in false negative results.

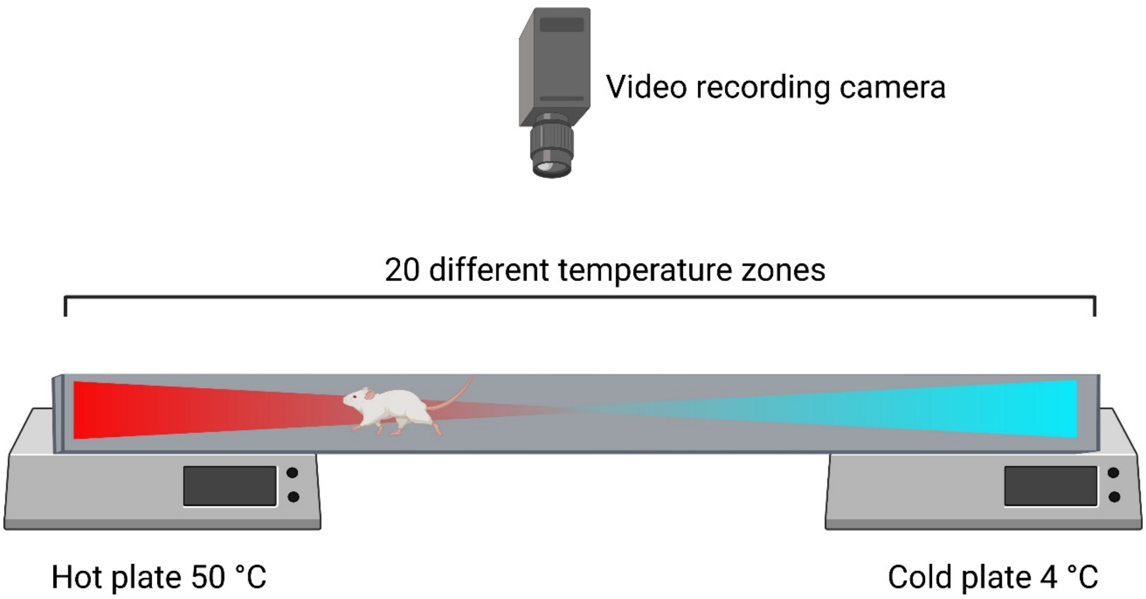

Figure 11. Example of thermal gradient setup*. Hot and cold plate are set so that the highest temperature is 50 °C and the coldest 4 °C generating a gradient of 20 different temperature zones in a metallic base plate. Mice are gently placed their designated arena for a 90-minute trial. A camera located directly above the apparatus feeds to video recording software that measures the amount of time spent and number of entries in each of the temperature zone as well as the average speed and acceleration. Image generated using BioRender.com.*

B. Post-endometriosis induction measurements and data plotting

1. No habituation prior to the trial day or baseline measurement are required.
2. Gently place the mice in their designated arena and put the cover on top.
3. Looking at the live feed from the camera confirm that both mice are highlighted while moving affirming that they are being properly tracked by the camera.
4. Start the trial and quietly leave the room.
5. After the 90-minute trial return the procedure room and return the mice to their home cages.
6. Export the statistics.
7. Take the mean velocity of all the mice during the 30-minute exploration time, this gives us the maximum velocity. The data from the 30-minute exploration period is no longer required after calculating the maximum velocity.
8. Exclude data from all-time segments for each mouse where the mouse moved at a velocity greater than the maximum velocity during the hour after the exploration period. This data is excluded because the high velocity the mouse was traveling at implies that it was not showing a preference for the zones it passed through and rather was moving in an exploratory fashion.
9. For each mouse sum the cumulative time spent in each zone. This data can be used to map the time spent in each temperature zone for each mouse.
10. To get the mean time spent in each temperature zone for each group take the average of the previously calculated sums of the mice in the specified group. This data can then be used to create a heatmap of the mean time spent in each temperature zone.

C. Post-testing

1. See SOP1: General setup, habituation, and post-test procedures.

SOP 12: Nesting

Purpose

Nesting is a natural behaviour of mice in that creating nests is evaluated as an estimation of their well-being. The assay requires to set up testing cages with nesting material in advance, to determine the ability/capacity of mice to "build" quality nests. Nest quality is defined as the extent/degree to which the nesting material (i.e., cotton) has been processed/elaborated/manipulated by the mouse to create the nest. This procedure is described by Tejada e*t al*., 2022.

Relevance to humans

Nesting is an innate / ethological behaviour observed in rodents and is used in the study of neurological disorders characterised by diminished well-being and impaired activities of daily living (Neely *et al.,* 2019). The Time to Incorporate to Nest Test (TINT) is also used as a method for identifying moderate to severe distress and pain in mice (Gallo *et al*., 2020), with clear outcomes. Nest building often failed after treatment with buprenorphine, so this should be considered in the design of assays. This test may serve as a proxy measure for wellbeing and quality of life, that we know are reduced in patients with endometriosis.

Supplies and equipment

The following equipment list is specific for nest building in <u>mice</u>. Only equipment directly used for carrying out this method are included. Additional items considered to be standard laboratory equipment and PPE are not listed.

- Clean home cage
- Sterile cotton
- Camera

PROTOCOL

A. Set up and overall procedure (Day -1)

1. See SOP1: General setup, habituation, and post-test procedures.

B. Post endometriosis induction measurements and data plotting

1. No habituation or baseline measurement are required.
2. Start by preparing cages (just with bedding in the bottom, no enrichment items) by placing six compressed cotton pieces in each of the spaces resulting from virtually dividing the cage in 6 equivalent squares (Figure 12A).
3. Place animals individually in each of the cages prepared as described above and allow the animals to settle for 3 hours in their designated cage.
4. After that period gently return the animal to the home cage and take a photo of the nesting cage.
5. Nesting (i.e., the ability/ capacity of mice to "build" nests) is evaluated by the investigator attending to two parameters: a) number of empty areas and b) quality of the nest.
6. Number of empty areas: Area is considered empty if it is clear of cotton material (Figure 12B).
7. A nest quality score is assigned to each cage depending on the degree of breakdown/breakage of the cotton ball as follows: 1 = cotton ball untouched/not broken, 2 = cotton ball partially broken, 3 = cotton is totally broken (Figure 12C).

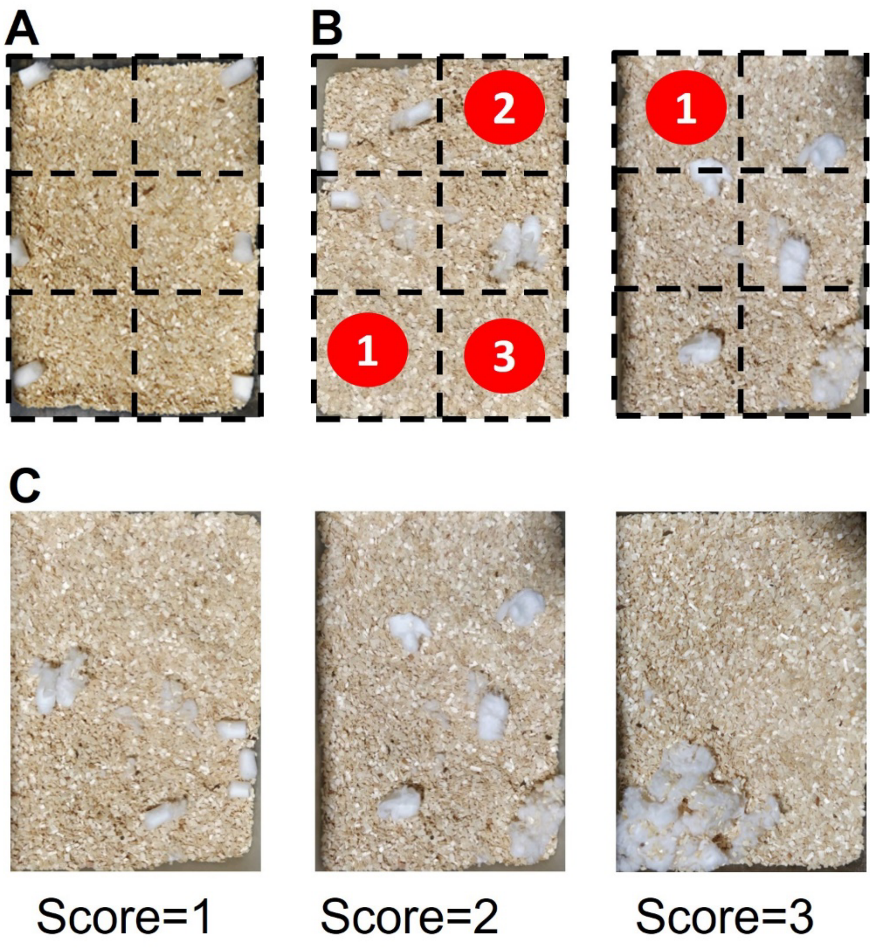

Figure 12. Illustrative images of set up of nesting and results obtained: *A) Set up of nesting test cages showing a cage virtually divided into 6 equivalent areas and a piece of cotton placed in each of them. B) Illustrative images of empty spaces after 3 h. Numbers into red circles denote the areas found empty in each case. C) Illustrative examples of different nest quality scores: 1 = cotton balls not broken, 2 = cotton balls partially broken, 3 = cotton balls broken completely.*

C. Post-testing

1. See SOP1: General setup, habituation, and post-test procedures.

REFERENCES

Gallo MS, Karas AZ, Pritchett-Corning K, Garner Guy Mulder JP, Gaskill BN. Tell-tale TINT: Does the Time to Incorporate into Nest Test Evaluate Postsurgical Pain or Welfare in Mice? *J Am Assoc Lab Anim Sci*. *2020*; 59(1): 37-45.

Neely CLC, Pedemonte KA, Boggs KN, Flinn JM. Nest Building Behavior as an Early Indicator of Behavioural Deficits in Mice. *J Vis Exp*. *2019*; (152).

Tejada MA, Santos-Llamas AI, Escriva L, Tarin JJ, Cano A, Fernández-Ramírez MJ, Nunez-Badinez P, De Leo B, Saunders PTK, Vidal V et al. Identification of Altered Evoked and Non-Evoked Responses in a Heterologous Mouse Model of Endometriosis-Associated Pain. *Biomedicines*. *2022*; 10(2): 501.

SOP 13: Burrowing

Purpose

Burrowing is an ethologically relevant behaviour in rodents, as it is a social conduct important for building underground habitats and nests and is highly conserved in laboratory animals. Suppressed burrowing, quantified by a reduced amount of displaced substrate from an artificial burrow, is indicative of behavioural dysfunction and may be used to assess the global impact of pain on animals. This protocol was adapted from (Wodarski *et al*., 2016). This assay appears to be more effective in rats than mice.

Relevance to humans

Similar to nest building, this assay is a proxy measure for wellbeing and quality of life that are disrupted in those with endometriosis.

Supplies and equipment

The following equipment list is specific for burrowing in mice and rats. Only equipment directly used for carrying out this method are included. Additional items considered to be standard laboratory equipment and PPE are not listed.

Burrowing material

- Rat: Gravel, 5-10mm pea shingle, 2.5kg per burrow (preferably autoclaved)
- Mouse: Sand, 0.3mm, 0.5kg per burrow (sifted and re-used; replaced as needed)

Burrowing tube

- Rat: Plastic, 320mm (length) x 100mm (diameter), raised 60mm from cage floor
- Mouse: Plastic, 200mm (length) x 75mm (diameter), raised 30mm from cage floor
  Note: *Burrowing tubes should be cleaned between test sessions*

Test cage

- For both rodent species, standard home cages that are changed after each test session and contain only paper towel/absorbent sheet at the base.

PROTOCOL

A. Set up and training (Day -1)

1. See SOP 1: General setup, habituation, and post-test procedures.
2. Ensure procedure room lighting is approximately 50 lux.
3. No habituation is needed for rats.
4. For mice, place pairs in test cage with an empty burrow tube for 1-2 hours, then return to home cage.

Rats

5. Rats should undergo behavioural testing during the light phase between 7am – 1pm.
6. Acclimatise rats in pairs for 30 minutes in their home cage in the procedure room, then 30 minutes in an empty test cage, with food and water removed.
7. Add burrowing tube filled with 2.5 kg gravel into test cage for 1 hour, then return rats to home cage.
8. If a pair of rats did not burrow during the first session one in the pair will be swapped with a rat from a burrowing pairing. This is called social facilitation. Repeat the training protocol on a consecutive day.

9. Rats that did burrow and are not needed for social facilitation also undergo the second training session so that every rat undergoes the same number of training sessions. Single rats that did not burrow may be excluded at this stage as a small percentage of rats simply do not engage in the behaviour.

Mice

10. Mice should undergo behavioural testing during the 12-hour light phase.
11. Unlike rats, mice dislike the burrow tube being added to the test cage, so pairs of mice are placed in the test cage with the burrow tube filled with 0.5 kg sand in place at start of 2-hour test session.
12. Following this 2-hour period, return mice to home cage.
13. Repeat final two steps of the rat burrow training protocol, above.

B. Behavioural testing

1. Repeat experimental conditions for both species as described in Step 1, above.

Rats

2. Each rat is assigned a test cage position on the cage rack (1 animal per cage), which remains unchanged throughout the study.
3. The same burrowing protocol is followed as described in Step 1, above, except that individual rats are present in each test cage.
4. At the end of the 2-hour test period, the weight of the gravel removed from the burrowing tube is recorded.
5. Two baseline measurements should be recorded per rat prior to endometriosis induction.
6. Animals that burrow less than 2 SD of total mean burrowed at any of the baseline sessions are excluded from the study.

Mice

1. Each mouse is assigned a test cage position on the cage rack and a burrowing tube (numbered), which remains unchanged throughout the study.
2. The same burrowing protocol is followed as described in Step 1, above, except that individual mice are present in each test cage.
3. At the end of the 2-hour test period, the weight of the sand removed from the burrowing tube is recorded.
4. Final two steps of the rat burrow test protocol, above.

REFERENCE

Wodarski R, Delaney A, Ultenius C, Morland R, Andrews N, Baastrup C, Bryden LA, Caspani O, Christoph T, Gardiner NJ et al. Cross-centre replication of suppressed burrowing behaviour as an ethologically relevant pain outcome measure in the rat: a prospective multicentre study. *Pain*. *2016*; 157(10): 2350-2365.

SOP 14: Home-cage analysis (HCA)

Purpose

The home cage analyser device (https://www.actualanalytics.com/) is intended to automatically report behavioural parameters occurring "in-cage". This can be applied as a wellbeing measure performed free of operator handling, in an automated manner, and into a more natural environment (i.e., the home cage) so social interactions between animals can be studied. The specialised cage which contains a base plate that detects the RIFD chips allows non-invasive assessment 24 hours a day, 7 days a week. The cage is mounted in a specialized rack with an associated video recorder for additional outputs (see https://www.actualanalytics.com/actualhca-mouse-desktop).

Relevance to humans

Home cage monitoring platforms offer unique insights into the structure of, and alterations in spontaneous ethological behaviours in rodents, over extended periods without human interference (Mingrone *et al.*, 2020). This method of longitudinal monitoring may have more relevance to humans because the prolonged period of testing (compared to an overreliance on traditional evoked tests that are brief) mimics a clinical trial setting and may lead to improved translation from preclinical to clinical settings.

Supplies and equipment

The following equipment list is specific for HCA in mice. Only equipment directly used for carrying out this method are included. Additional items considered to be standard laboratory equipment and PPE are not listed.

- Actual HCA Capture Program and Analyzer™ (Actual Analytics Ltd; Edinburgh, UK)
- Standard ISO 2 × 12 mm pre-packed and sterile RIFD tag microchip (PeddyMark; Elsenham, UK).
- Isoflurane

PROTOCOL

A. Set up and overall procedure (Day -1)

1. See SOP1: General setup, habituation, and post-test procedures.

Microchip implantation

2. Anesthetize the animal with isoflurane.
3. With the help of tweezers, lift the skin near the groin (Figure 13) to be able to insert the needle containing the microchip.
4. Application can be performed with a commercially available gun device consisting of a plunger engaged to a needle through which the microchip is pushed to ease insertion. If no plunger is available, proceed by creating a small pocket with the tweezers and firmly push the chip until insertion is complete.
5. If chip insertion is adequate, it should get firmly trapped subcutaneously and no stitch should be required. If not sure that placement was adequate, use a little biological glue or suture to close the pocket gap.
6. Microchip needs to be placed in the ventral part of the animal, as it is closer to the base (where the antennas are located).
7. It is critical that special care is taken during the application to ensure the microchip will not fall into the intraperitoneal cavity as this will cause discomfort and interfere with subsequent measurements.
8. Once microchips are placed, gently place animals back to their cage and allow them to rest for a few days to allow their behaviour return to normal activity.

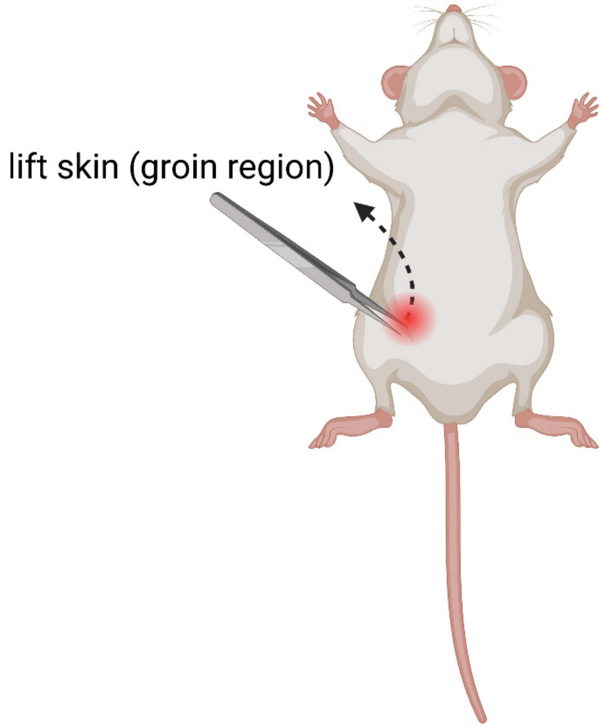

***Figure 13. Microchip implantation**: Under isoflurane anaesthesia and with the help of tweezers, lift the skin near the groin to be able to insert the needle containing the microchip (red spot). Care should be taken to inject not it into the intraperitoneal cavity. Image generated using BioRender.com.*

<u>Monitoring activity</u>

9. To monitor activity, place the animal into the desired cage and place it in the HCA system.
10. Adjust the position of the cage to the boundaries of the grid in the bottom of the device containing the antennas.
11. Width and length of the grid are made to fit 1285L cages of the blue line, and therefore, one needs to use that specific type or cages similar in dimensions.
12. Once the cage is ready, click the HCA icon to open associated software for capture (*Actual HCA capture program)*, label the experiment by typing a name or code as desired, indicate the whole duration of recording and the periods for automatic savings of data and click “start recording”.
13. The program allows to set up duration of the recording as desired from 1 minute to 7 days. Data can be saved in periods of 15, 30, 45, or 60 minutes.

B. Post endometriosis induction measurements and data plotting

1. No prior habituation is required but baseline measurement of behaviour is required because this can vary between cages.
2. At the end of the study, Actual HCA Analyser™ software is used to automatically generate a report in a *.csv file format, which may be opened with Microsoft Excel software.
3. Raw data appear shown in different columns identifying the study (date, time, subject) and the parameters analysed (distance, time drinking, climbing, thigmotactic, isolated or in centre zones, among others).
4. Rows are filled with data obtained per saved period. At every time point (i.e., periods of 30-60 minutes), data are collected for each of the mice (identified by their specific microchip code).

5. Care must be taken to avoid errors so as not to mix data from different animals and thus appropriately extract data from each animal during the time course.
6. Label rows in different colours for cage group might help visualization. Figure 14 shows an illustrative example of aligned data after data extraction.

date	time	subject	distance (mm)
21/09/2021	09:30:00	900200000785037	13516,08201
21/09/2021	10:00:00	900200000785037	9870,698106
21/09/2021	10:30:00	900200000785037	3456,783396
21/09/2021	11:00:00	900200000785037	170,7106807
21/09/2021	09:30:00	900200000780681	13406,00319
21/09/2021	10:00:00	900200000780681	15705,57495
21/09/2021	10:30:00	900200000780681	3543,276304
21/09/2021	11:00:00	900200000780681	128,5238247
21/09/2021	09:30:00	900200000780675	12161,82815
21/09/2021	10:00:00	900200000780675	6759,393569
21/09/2021	10:30:00	900200000780675	617,316084
21/09/2021	11:00:00	900200000780675	0

Figure 14. Raw data exported. *Illustrative example of aligned data in an excel file after extraction from a raw csv data HCA report. Subject column refers to the microchip identifying data recorded (i.e., data for each specific mouse). Rows are coloured to identify time course for each mouse.*

REFERENCE

Mingrone A, Kaffman A, Kaffman A. The Promise of Automated Home-Cage Monitoring in Improving Translational Utility of Psychiatric Research in Rodents. *Front Neurosci*. *2020*; 14: 618593.

SOP 15: Open field

Purpose

The test is used to evaluate exploratory activity of rodents in a novel or non-novel arena as a measure of anxiety if arena is novel and voluntary locomotor activity if arena non-novel. During the test, a rodent is placed in the arena and left to explore for a specified length of time. During the exploration, a rodent will typically remain close to the side of the arena (thigmotaxis) and avoid the centre as the centre is considered an area of high risk due to openness. Reduced time in the centre relative to healthy control rodents indicates increased anxiety levels.

Relevance to humans

The open field test allows for the assessment of spontaneous rodent behaviour and general locomotor activity in a novel environment and as a tool to assess anxiety-like behaviour. Since animals are tested alone and are placed in an arena with bright/dark areas this causes anxiety which has been used to test therapeutics (Prut and Belzung, 2003). To what extent this anxiety-like defensive response truly reflects the same physiological responses found in human anxiety is still unclear. However, studies comparing thigmotaxis in both rodents and humans indicated a strong similarity in behaviour across species for anxiety-related disorders (Gromer *et al*., 2021).

Supplies and equipment

The following equipment list is specific for open field in rodents. Only equipment directly used for carrying out this method are included. Additional items considered to be standard laboratory equipment and PPE are not listed.

- Open field apparatus with associated infrared beams and software for automated analysis (e.g., Med Associates, USA, cat number MED-OFA-017 / -022) or an arena with a camera located above the arena associated with video tracking software (e.g., Ethovision XT; Noldus BV, Netherlands; ANY-Maze; Stoelting Inc, USA).

PROTOCOL

A. Set up and overall procedure (Day -1)

1. See SOP 1: General setup, habituation, and post-test procedures.
2. The room should be lit by indirect lighting, approximately 30 lux.
3. Set up the software and run a diagnostic to ensure the infrared beams are all working.

B. Post-endometriosis induction measurements and data plotting

1. Bring animals to procedure room for 30 minutes prior to testing to acclimatise, or transport individually to the testing room in their home cages.
2. Gently place one rodent against a wall in the periphery of each arena and allow time to explore.
3. Record exploration for approximately 5 minutes.
4. After the exploration time finishes, remove rodent from the arena and return to their home cage.
5. Ensure all equipment is clean (i.e., wiped down with disinfectant and/or 70% ethanol) before testing another round of animals.
6. Multiple measures of activity and zone exploration can be calculated such as total distance travelled, time spent in the centre of the arena, time spent near the walls of the arena (periphery), number of rearings, number of entries to the centre of the arena, number of entries to the periphery of the arena, and mean speed.

C. Post-testing

1. See SOP1: General setup, habituation, and post-test procedures.

REFERENCES

Gromer D, Kiser DP, Pauli P. Thigmotaxis in a virtual human open field test. *Sci Rep*. *2021*; 11: 6670.

Prut L, Belzung C. The open field as a paradigm to measure the effects of drugs on anxiety-like behaviours: a review. *Eur J Pharmacol*. *2003*; 463(1-3): 3-33.

SOP 16: Exploratory behaviour

Purpose

Paradigms of altered affective states in rodent pain models include reduced exploratory behaviour. Exploratory activity here is recorded in a modification of the open field setting, similar to the thigmotaxis assay which is an example of an ethological behavioural outcome. A/Prof. Erin Greaves' team developed this protocol and performed exploratory behavioural tests starting 21 days after endometriosis induction (mouse) on 2-3 consecutive days (Greaves *et al*., 2017).

Relevance to humans

See SOP15.

Supplies and equipment

The following equipment list is specific for exploratory behaviour in mice. Only equipment directly used for carrying out this method are included. Additional items considered to be standard laboratory equipment and PPE are not listed.

- Rodent home cage and new cardboard tunnel
- Stopwatch

PROTOCOL

A. Setup

1. See SOP1: General setup, habituation, and post-test procedures.
2. This test should not be conducted on the same day after a cage/bedding change, within 2 days of establishing new group housing setups, or during the recovery phase following a procedure/extensive handling.
3. This test takes place in the home cage but with a fresh cardboard tunnel placed in the centre of the cage. Mice are moved to the room where testing will take place and allowed to settle from the move for 30 minutes prior to the cage lid being removed and a fresh cardboard tunnel being placed.

B. Exploratory behaviour testing

1. Open field tunnel entries are manually recorded for each mouse by two independent investigators (blinded to experimental group).
2. This is performed for 2 x 5 minutes (within a 30-minute window; 5 minutes after removal of cage lid and placement of tunnel and 20 minutes after cage lid removal) and an average is generated.

C. Post-test

1. See SOP1: General setup, habituation, and post-test procedures.
2. Testing should be repeated over 2-3 consecutive days, and an average result taken to minimize random errors/behavioural discrepancies.

REFERENCE

Greaves E, Horne AW, Jerina H, Mikolajczak M, Hilferty L, Mitchell R, Fleetwood-Walker SM, Saunders PTK. EP2 receptor antagonism reduces peripheral and central hyperalgesia in a preclinical mouse model of endometriosis. *Sci Rep*. *2017*; 7: 44169.

SOP 17: Elevated plus maze

Purpose

The elevated plus maze is used to evaluate exploratory activity of rodents in a novel arena where there is an aversive area (the open arms) and a safe area (the closed arms). The application of the task is therefore to measure anxiety in both mice and rats. During the test, a rodent is placed in the central square of the maze facing a closed arm and allowed 5 minutes to explore. During the exploration, a rodent will typically remain in the closed arms, showing risk assessment behaviours, such as stretching out onto the open arm while remaining in the closed area to assess the value of venturing onto the open arm.

Relevance to humans

The elevated plus maze utilizes spontaneous rodent approach versus avoidance behaviour. This assay utilises rodents' preferences for dark enclosed spaces to escape predators, and aversion to bright lit areas to assess "anxiety-like" behaviour in the animals, which can resemble anxiety responses in humans. Although the assay has been useful to demonstrate the effectiveness of some anxiolytics, caution must be exercised in interpretation as the mechanisms behind this fear-like response in the animals may not truly reflect anxiety in humans (Lezak *et al*., 2017).

Supplies and equipment

The following equipment list is specific for elevated plus maze in <u>mice</u>. Only equipment directly used for carrying out this method are included. Additional items considered to be standard laboratory equipment and PPE are not listed.

- Elevated plus maze apparatus
- ANY-Maze software (Stoelting; Wood Dale, IL, USA) for video tracking of the animal
- Computer
- Web cam

PROTOCOL

A. Measuring activity in the maze

1. Bring mice to room for 30 minutes prior to testing to acclimatize.
2. The room should be lit by indirect lighting, approximately 30 lux.
3. Set up the plus maze on the tabletop under a camera and set up Any-Maze software.
4. Before starting a test, clean equipment with bleach solution and then with 70% ethanol.
5. A mouse is placed in the central square where the open and closed arms intersect (the "decision zone") facing a closed arm and allowed 5 minutes to explore all arms of the maze (Figure 15).
6. After 5 minutes, remove mouse from the maze and return to home cage.

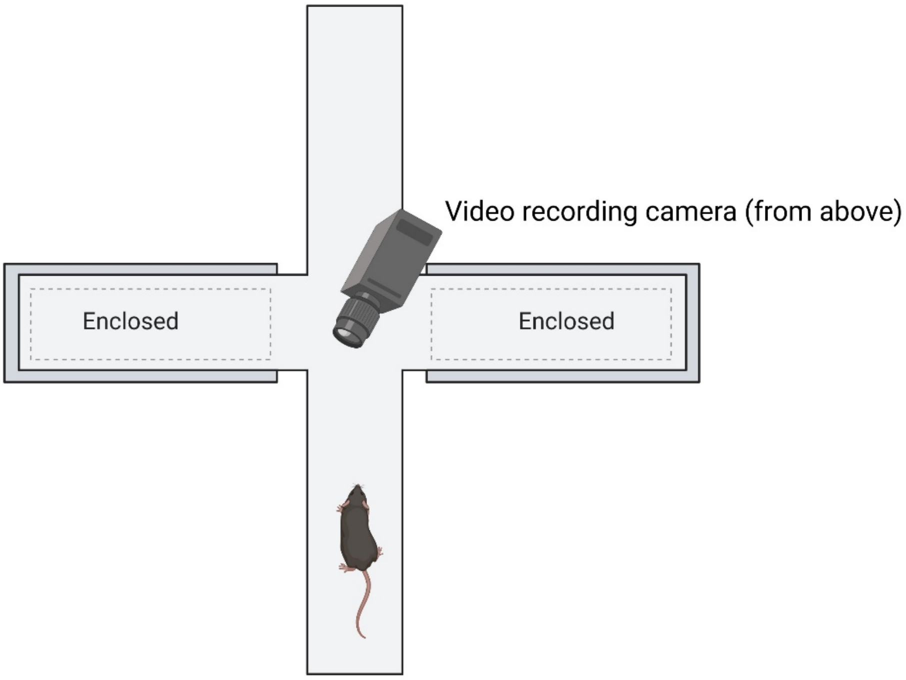

Figure 15. Example elevated plus maze*. A rodent is placed in the central square area formed at the intersection between the closed and open arms (the "decision zone"), facing one of the closed arms of the maze and allowed to explore. A camera located directly above the apparatus feeds to video recording software that measures the amount of time spent in the open, closed, and central sections of the maze. Image generated using BioRender.com.*

B. Post-test

1. See SOP1: General setup, habituation, and post-test procedures.

C. Data analysis

Time spent and number of entries into both the open and closed arms are extracted, and the % time in the open arms and % number of open arm entries are calculated (time in open arms divided by (time in open + time in closed) *100). The number of closed arm entries (or distance travelled in the closed arms) is considered the true measure of locomotor activity as it is less affected by open arm activity. A change in open arm activity in the absence of changes in closed arm distance travelled or entries is considered an effect of anxiety.

REFERENCE

Lezak KR, Missig G, Carlezon WA Jr. Behavioural methods to study anxiety in rodents. *Dialogues Clin Neurosci*. *2017*; 19(2): 181-191.

SOP 18: Elevated zero maze

Purpose: The elevated zero maze is used to assess anxiety and exploratory-like behaviour in rats. The time that rats spend in the open arms is considered to represent having less anxiety. This maze is similar to that of the elevated plus maze but has no central area so allows for uninterrupted continuous exploration (Braun *et al*., 2011). The protocol has been implemented by Dr Caroline Appleyard to assess the impact of stress interventions on anxiety in rats with endometriosis (Appleyard *et al*., 2021; Torres-Reverón *et al*., 2018).

Relevance to humans

See SOP17.

Supplies and equipment

The following equipment list is specific for elevated zero maze in rats. Only equipment directly used for carrying out this method are included. Additional items considered to be standard laboratory equipment and PPE are not listed.

- Red-light bulb
- Elevated Zero Maze apparatus (circle with 100 cm inner diameter, 10 cm lane width, and 50 cm leg height) in which two sections of the circle are enclosed by 30 cm high walls and two are open (no walls) (see Figure 16)
- ANY-Maze software (Stoelting; Wood Dale, IL, USA) for video tracking of the animal
- Computer
- Web cam

PROTOCOL

A. Setup

1. Bring animals to room for 30 minutes prior to testing to acclimatize.
2. Set up the zero-maze protocol in the ANY-maze software following the step-by-step guided process provided by the software (see HELP section).
3. Adjust the camera so the entire apparatus can be observed in the monitor.
4. Turn off the white lights in the room and turn on a red-light bulb.
5. Wipe and clean the apparatus with 70% ethanol and towel paper then allow time to dry.

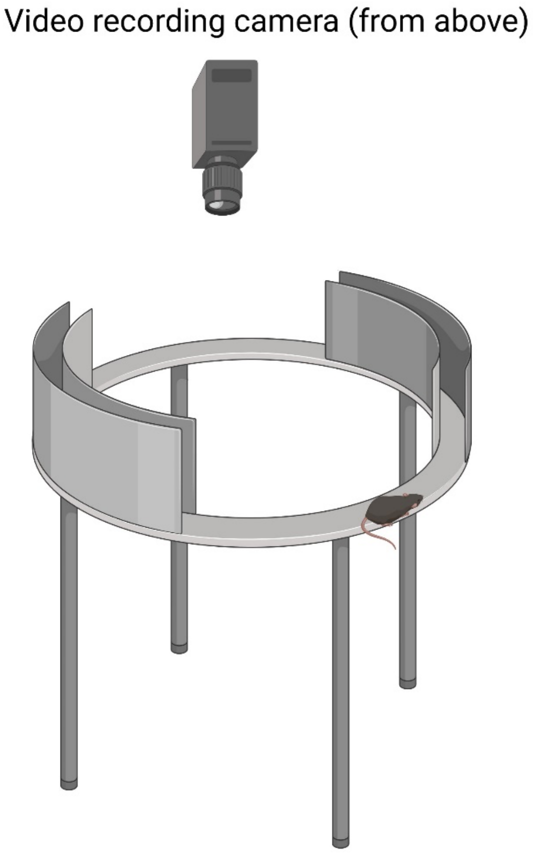

Figure 16. Example elevated zero maze. *A rodent is placed in the open section of the maze and allowed to explore. A camera located directly above the apparatus feeds to video recording software that measures the amount of time spent in the open, closed, and central sections of the maze. Image generated using BioRender.com.*

B. Recording

1. Place the rats one at a time facing a closed arm and opposite to the experimenter at the start of each trial.
2. Allow the rats to explore the apparatus for 5 minutes while video recording the session for later analysis.
3. Select the following parameters for analysis by the ANY-Maze program: total distance travelled, time spent in the open/closed sections and number of entries made by the rats onto the open/closed sections.
4. At the end of the 5-minute session, stop the recording, remove the animals and place back into their home cage.
5. Note the number of faecal pellets expelled (if any) during the session.
6. Discard the faecal pellets in biohazard bags.
7. Ensure all equipment is clean (i.e., wiped down with disinfectant and/or 70% ethanol) before testing next animal.

C. Post-test

1. See SOP 1: General setup, habituation, and post-test procedures.

D. Data analysis

Time spent and number of entries into both the open and closed sections are extracted and the % time in the open sections and % number of open section entries are calculated (time in open sections divided by (time in open + time in closed) *100). The number of closed section entries (or distance travelled in the closed sections) is considered the true measure of locomotor activity as it is less affected by open activity. A change in open activity in the absence of changes in closed distance travelled or entries is considered an effect on anxiety. Can also measure speed and latency to first entry.

REFERENCES

Appleyard CB, Cruz ML, Velazquez-Cruz J, Rivera-Mendez RM, Jimenez-Garcia JG, Rivera LA, Del Mar Mendez-Casillas M, Flores I, Al-Nakkash L, Chompre G. Voluntary Wheel Running Reduces Vesicle Development in an Endometriosis Animal Model Through Modulation of Immune Parameters. *Front Reprod Health*. *2021*; 3: 826541

Braun AA, Skelton MR, Vorhees CV, Williams MT. Comparison of the elevated plus and elevated zero mazes in treated and untreated male Sprague-Dawley rats: effects of anxiolytic and anxiogenic agents. *Pharmacol Biochem Behav*. *2011*; 97(3): 406-415.

Torres-Reverón A, Rivera LL, Flores I, Appleyard CB. Environmental Manipulations as an Effective Alternative Treatment to Reduce Endometriosis Progression. *Reprod Sci*. *2018*; 25(9): 1336-1348.
